# Microstructural insights into fast ion transport in solid electrolytes via multiscale modeling

**DOI:** 10.1038/s41467-026-76216-w

**Published:** 2026-08-20

**Authors:** Yongliang Ou, Lena Scholz, Sanath Keshav, Yuji Ikeda, Marvin Kraft, Sergiy Divinski, Rafael Gómez-Bombarelli, Wolfgang G. Zeier, Felix Fritzen, Blazej Grabowski

**Affiliations:** 1 Institute for Materials Science, University of Stuttgart, Stuttgart, Germany; 2 Department of Materials Science and Engineering, Massachusetts Institute of Technology, Cambridge, MA USA; 3 Institute of Applied Mechanics, University of Stuttgart, Stuttgart, Germany; 4 Institute of Inorganic and Analytical Chemistry, University of Münster, Münster, Germany; 5 Institute of Energy Materials and Devices (IMD), IMD-4: Helmholtz-Institut Münster, Forschungszentrum Jülich, Münster, Germany; 6 Institute of Materials Physics, University of Münster, Münster, Germany

**Keywords:** Atomistic models, Molecular dynamics, Batteries

## Abstract

Improving solid electrolytes is critical for high-performance all-solid-state batteries, yet the microstructural features that enable fast ion transport remain poorly understood. Here, we use multiscale modeling to resolve polycrystalline ion transport from atomic-scale hopping at grain boundaries to continuum-scale percolation, thereby providing insights into realistic solid-electrolyte microstructures. Accurate lightweight machine-learning potentials—developed via closed-loop active learning for exemplar argyrodites Li_6_PS_5_*X*, *X* ∈ {Cl, Br, I}—are employed to integrate molecular dynamics with finite element simulations. We find that diffusion barriers of the anion-ordered bulk scale linearly with anion radius. Grain boundaries exert opposite effects depending on the bulk: enhancing ion diffusion in low-diffusivity phases but suppressing it in fast-diffusing ones. Li_6_PS_5_I exhibits non-Arrhenius transport behavior consistent with experimental observations. Our results clarify the pivotal role of grain boundaries in ion transport and guide a priori microstructural design of advanced solid electrolytes.

## Abstract (web summary)

Here, the authors combine atomistic and continuum simulations to uncover how microstructure affects lithium-ion transport in solid electrolytes. The findings help guide the design of safer, higher-performance all-solid-state batteries.

## Introduction

The rising demand for all-solid-state batteries has placed solid-state electrolytes at the forefront of material research, with most efforts historically focused on optimizing superionic bulk conductivity^1–3^. However, practical solid electrolytes are polycrystalline, exhibiting microstructures with dense grain-boundary (GB) networks^4^. Macroscopic conductivity arises from long-range ion transport that inevitably involves both grain interiors and extensive GB pathways^5^. Microstructure thus represents a critical—yet often underutilized—lever for tailoring ion transport. Although GB effects in oxide electrolytes have been partially elucidated^6–8^, the microstructural features that enable fast ion transport remain poorly understood. Microstructural insights are particularly limited in sulfide electrolytes, where the role of GBs is still unclear due to the experimental difficulty of characterizing their morphology and separating their contributions to conduction.

Computer simulations can provide insights into complex microstructures by quantitatively predicting the microstructure–conductivity relation. While prior electronic-structure modeling has yielded valuable insights into bulk ion mobility^1,3,9^, modeling ion transport in polycrystalline solid electrolytes is far more difficult. The transport process spans atomic-scale hopping and continuum-scale percolation, both influenced by structural and thermal variations^10^, highlighting the need for a multiscale modeling framework^11^. Molecular dynamics (MD) simulations are widely used to investigate atomic-scale diffusion mechanisms, but applying them to polycrystals poses several challenges. First, interatomic potentials must describe complex ionic interactions at GBs. Second, large simulation cells comprising millions of atoms are required to represent the structural disorder inherent to GBs in polycrystals. Third, long simulation times are needed to resolve the equilibrated GB structures (unknown a priori), and to obtain statistically converged diffusivities. Classical potentials generally lack the fidelity to describe intricate atomic interactions at GBs. While ab initio MD based on density-functional theory (DFT) provides high accuracy, its computational cost has restricted prior studies to idealized models with single GBs^12,13^. Recent machine-learning potentials offer a promising alternative, but parameterizing them to balance accuracy and computational cost is non-trivial^14–16^. Even with these potentials, MD simulations of solid electrolytes remain limited to nanometer length scales and nanosecond timescales^16,17^. Finite element (FE) simulations provide a rigorous way for evaluating macroscopic properties of heterogeneous microstructures at the continuum scale. Nevertheless, resolving thin and anisotropic GBs in polycrystals and maintaining their applicability across the wide range of diffusion kinetics in solid electrolytes is difficult^18^. Finally, robust schemes for bridging simulations across scales remain scarce, with most previous studies addressing each scale independently (Supplementary Table 3; Refs. ^10,18^).

**Fig. 1:**
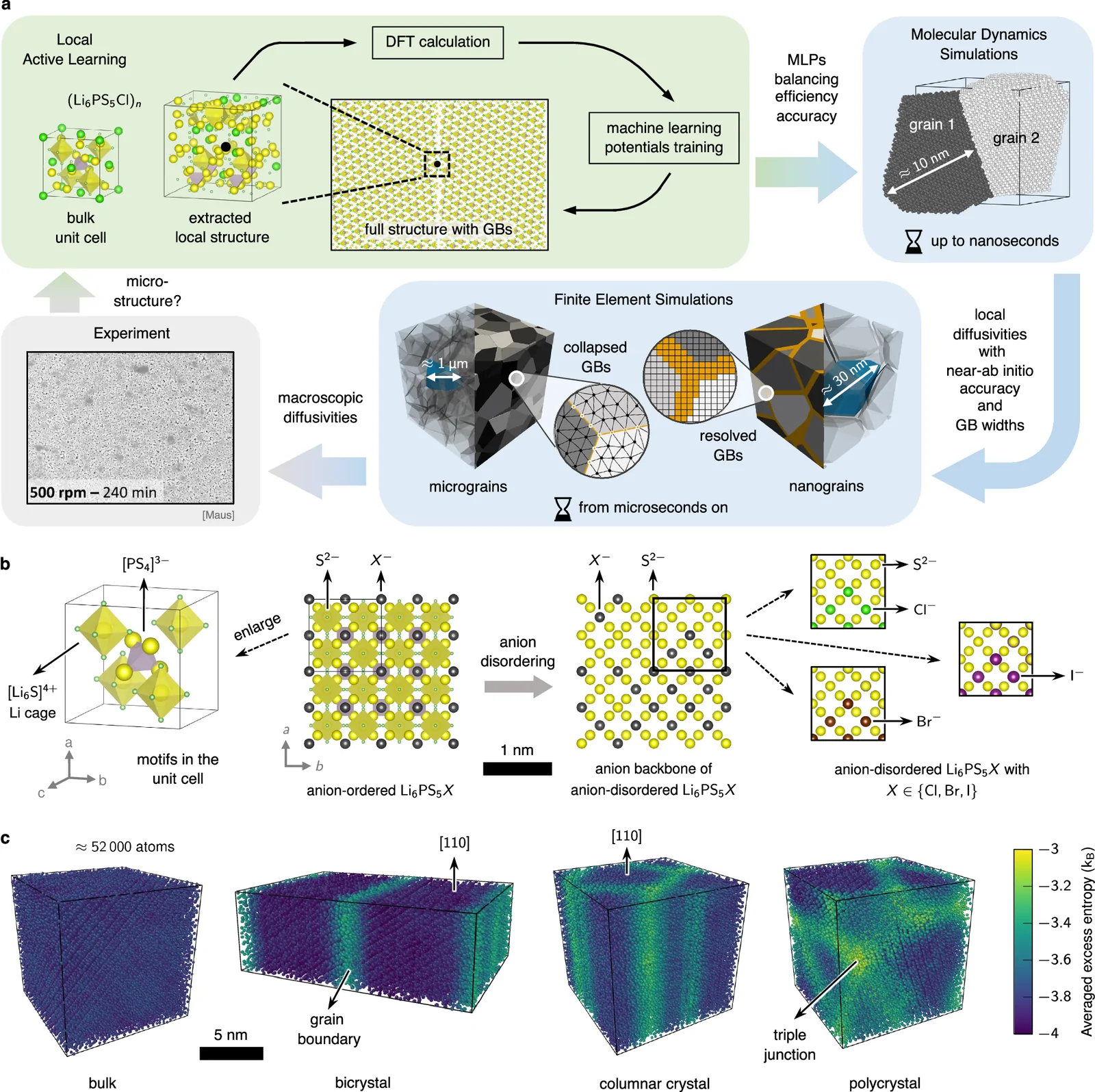
Multiscale modeling framework to resolve Li-ion transport and atomic and microstructural complexity of argyrodite solid electrolytes Li_6_PS_5_*X* with *X* ∈ {Cl, Br, I}. **a** Lightweight machine-learning potentials (MLPs) balancing accuracy and efficiency are developed with closed-loop active learning, based on density-functional theory (DFT) calculations of local structures extracted from full microstructures with grain boundaries (GBs). These potentials are applied in molecular dynamics (MD) simulations to obtain local Li-ion diffusivities. Finite element (FE) simulations, parameterized with MD-derived diffusivities and GB widths, are then performed on micrograins with collapsed GBs and on nanograins with resolved GBs using Fourier-accelerated nodal solvers, yielding macroscopic diffusivities. The resulting predictions can be quantitatively compared with experimental measurements, and, when available, experimental microstructure data can inform end-to-end experiment–simulation studies. A scanning electron micrograph of cathode composites containing processed solid electrolytes Li_5.5_PS_4.5_Cl_1.5_ adapted from Maus et al.^29^ is shown as an example, in which GBs are poorly resolved. **b** Atomic structures of bulk argyrodites featuring Li cages and ortho-thiophosphate ions. Structural and chemical variations include anion disorder and halide substitution. **c** Atomistic models of microstructures including bulk phases, bicrystals with single-type GBs, columnar crystals, and polycrystals with random GBs. The rotation axis is [110] for both bicrystals and columnar crystals. Triple junctions occur in columnar crystals and polycrystals, and both GBs and triple junctions exhibit increased excess entropy, indicating enhanced structural disorder.

Here, we propose a computational strategy to address the challenge of modeling ion transport in polycrystalline solid electrolytes (Fig. 1a). We first develop a closed-loop active learning scheme based on DFT calculations of local atomic environments for parameterizing lightweight machine-learning potentials, specifically moment tensor potentials^19^. These potentials are applied in large-scale MD simulations of a surrogate GB model with random GBs, capturing diffusion kinetics while accounting for thermodynamics and GB heterogeneity. Then, we deploy the MD-derived self-diffusivities in FE simulations, modeling micrograins with collapsed GBs^18^ and nanograins with resolved GBs using Fourier-accelerated nodal solvers^20^. This approach provides a hierarchical resolution of Li-ion transport from the electronic-structure level to the device-relevant behavior, delivering quantitative predictions comparable with experimental measurements and enabling end-to-end experiment–simulation studies.

We apply the proposed strategy to exemplar sulfide solid electrolytes, Li_6_PS_5_*X* argyrodites with *X* ∈ {Cl, Br, I}, which exhibit some of the highest known ionic conductivities^21^ and are promising for applications. The morphologies and energetics of diverse GBs, including low-angle types, are systematically investigated. We evaluate GB impact on Li-ion transport in anion-disordered Li_6_PS_5_Cl and examine the effect of substituting Cl with Br or I. Integrating the resulting diffusivities into continuum-scale simulations quantifies the bulk-dependent grain-size effects in argyrodites. Overall, these findings provide insights for designing solid-electrolyte microstructures.

## Results

### Energetics and diffusivity of GBs in anion-ordered argyrodites

Argyrodite solid electrolytes exhibit a high degree of chemical complexity and compositional tunability. In fully ordered Li_6_PS_5_*X*, the bulk structure consists of Li-coordinated cages (Li_6_S^4+^) and ${{{\rm{PS}}}}_{4}^{3-}$ tetrahedra (Fig. 1b). The translationally immobile anion sublattice forms a backbone for Li^+^ interstitial diffusion, and partial occupancy of S^2−^ and *X*^−^ introduces chemical disorder.

Microstructure adds an extra layer of complexity in argyrodites. In GBs, structural and chemical disorder are coupled. To capture these effects, we investigate GBs embedded in three representative atomistic models (Fig. 1c). Bicrystal models contain a single GB type, enabling isolated analysis of specific GB structures. Columnar crystals feature multiple GBs intersecting along a common rotation axis, whereas polycrystals form random GB networks. Both columnar and polycrystalline models include triple junctions in addition to GBs.

Experimental characterization of microstructure in sulfides remains challenging, leaving GBs in synthesized samples poorly resolved (Fig. 1a). In simulations, this implies that the type, distribution, and atomic structure of GBs are unknown a priori and must be determined in silico. We construct bicrystal models of anion-ordered Li_6_PS_5_Cl with single-type GBs to probe GB morphology. GBs are labeled by the coincidence-site lattice index Σ, defined during model construction, indicating structural complexity. Equilibrated GB structures, incorporating Li-ion disorder from diffusion and anion-sublattice distortions induced by GBs, are obtained using an MD-based annealing-and-quenching protocol^22^ (see “Methods”).

**Fig. 2:**
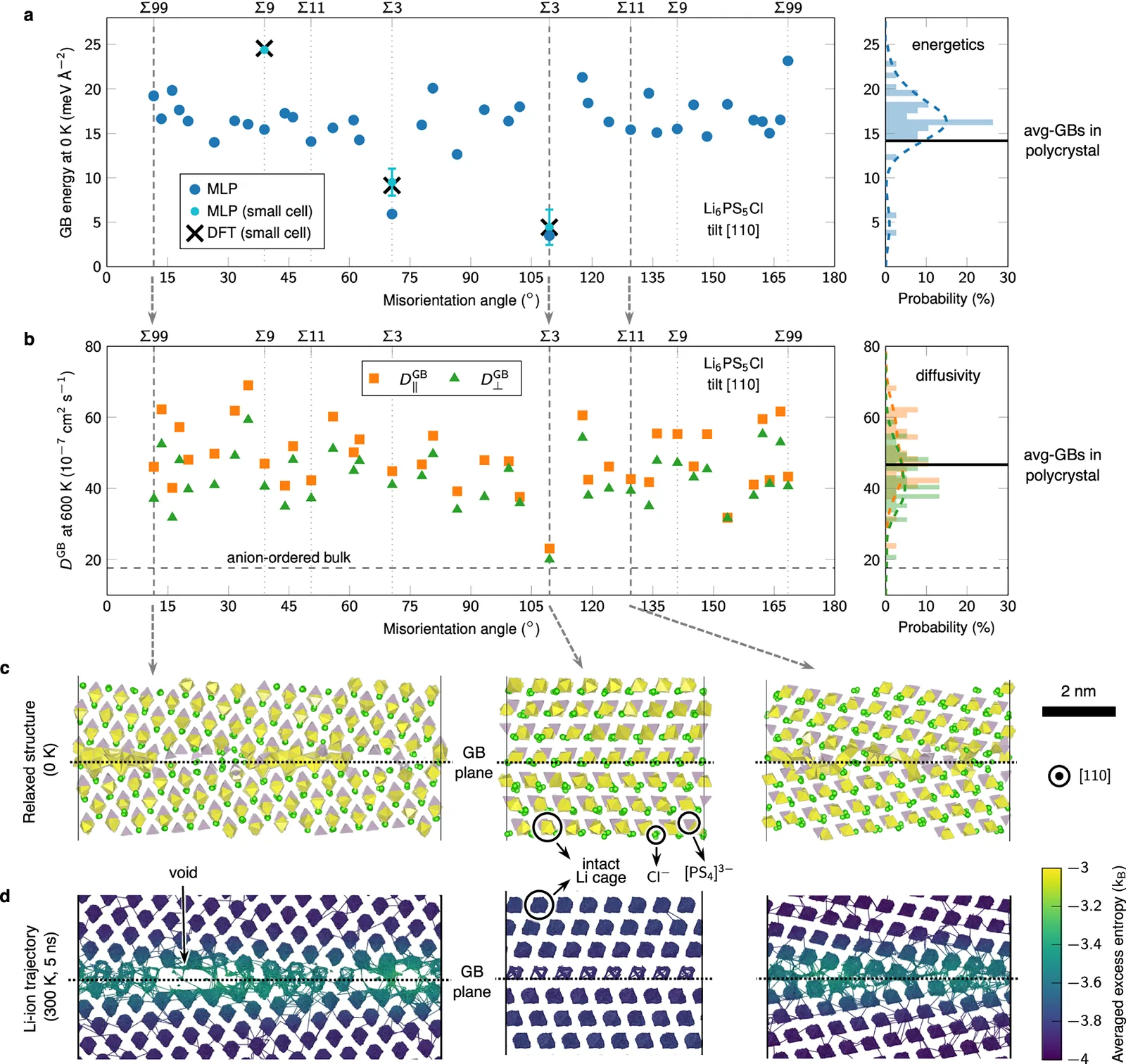
GB impacts in anion-ordered Li_6_PS_5_Cl. **a** Predicted GB formation energy at 0 K. GB energies of Σ3 and Σ9 GBs in small simulation cells were calculated by density-functional theory (DFT) based on the structures relaxed by machine-learning potentials (MLPs), which validates the accuracy of MLPs. Error bars show the standard deviation across five independent potentials. **b** Predicted GB self-diffusivities *D*^GB^ at 600 K. Diffusion within and across the GB planes is represented by ${D}_{\parallel }^{{{{\rm{GB}}}}}$ and ${D}_{\perp }^{{{{\rm{GB}}}}}$, respectively. Li-ion diffusivity is lower in the anion-ordered bulk than in GBs, and ${D}_{\perp }^{{{{\rm{GB}}}}}\approx 0.87{D}_{\parallel }^{{{{\rm{GB}}}}}$. Single-type tilt GBs were constructed with a rotation axis [110]. Probability distributions of GB energy and diffusivity are shown, both exhibiting small variations in the GB configuration space, except for Σ3 GBs. GB energy and diffusivity averaged over all GBs within a polycrystalline model are consistent with those from single-type GBs. **c** Relaxed atomic structures of Σ99, Σ3, and Σ11 GBs at 0 K. Irregular yellow polygons at the Σ99 and Σ11 GBs highlight complex Li–S coordination. **d** Li-ion trajectories from MD simulations at 300 K over 5 ns for the three selected GBs. In Σ99 and Σ11, GBs alter Li–S coordination of Li cages, producing structural disorder evidenced by increased excess entropy. Sub-nanometer voids appear in some GBs, such as Σ99.

Figure 2a shows that the Σ3 GB at 109° has the lowest formation energy,  ~5 meV Å^−2^, whereas other GBs, including low-angle GBs, range from 12 to 24 meV Å^−2^. Differences in formation energy stem from GB morphology, with each type imposing different structural modifications on the lattice. In the Σ3 GB, Li-cage and ${{{\rm{PS}}}}_{4}^{3-}$ environments are largely preserved, with rearrangements of these units and the halide ions, evident in the relaxed structure (Fig. 2c). By contrast, general GBs heavily distort Li cages and ${{{\rm{PS}}}}_{4}^{3-}$ units, leading to complex Li–S coordination, anion-sublattice disorder, and lattice distortions. Forming GBs without altering Li-cage and ${{{\rm{PS}}}}_{4}^{3-}$ units is expected to be rare, as GBs are two-dimensional defects distributed throughout the bulk. These results underscore the importance of simulating realistic GBs beyond idealized cases such as Σ3 (cf. previous studies in Supplementary Table 3). The GB formation energies of Li_6_PS_5_Cl are low compared with oxide electrolytes (25–56 meV Å^−2^, Ref. ^23^), and sulfide-based argyrodites are mechanically soft, both factors likely promoting the formation of abundant and random GBs in synthesized samples.

We investigate diffusion in GBs using MD simulations of equilibrated bicrystal models, capturing Li-ion disorder and correlations, multiple diffusion pathways, GB-induced lattice distortions, and anion-sublattice vibrations. GB diffusivity and energetics follow a similar trend: The Σ3 GB at 109° has the lowest diffusivity, while others reach 3 × 10^−6^ to 7 × 10^−6^ cm^2^ s^−1^ (Fig. 2b). At 600 K, GB diffusivity is about threefold higher than in the bulk and slightly anisotropic, with faster Li-ion transport within the GB plane (Supplementary Fig. 16). Li-ion trajectories at 300 K (Fig. 2d) reveal that GB-induced lattice modifications create new low-barrier pathways, promoting inter-cage Li-ion hopping. Excess entropy (see “Methods”) quantifies the increased structural disorder at GBs arising from the anion sublattice and mobile Li ions. Subnanometer voids form at some GBs but have little effect on GB diffusivity.

Each bicrystal represents a single GB configuration, providing a simplified model without triple junctions. To probe general configurations, we construct two-grain polycrystals embedded with random GB networks, serving as surrogate GB models (see “Methods”). Their averaged energetics and Li-ion diffusivities fall within the range of single-type GBs (histograms in Fig. 2a, b). Columnar and polycrystalline models show comparable Li-ion diffusivity (Supplementary Fig. 17). These results indicate that triple junctions have a minor effect in polycrystalline Li_6_PS_5_Cl.

### GBs in argyrodites with anion disorder and substitution

Our analysis has so far focused on anion-ordered Li_6_PS_5_Cl. In argyrodite solid electrolytes, anion chemistry—particularly disorder and substitution (Fig. 1b)—critically influences Li-ion transport. In the bulk lattice, site exchange between S^2−^ and *X*^−^ markedly promotes inter-cage Li^+^ diffusion^24,25^. Still, it remains unclear how GBs are coupled with anion chemistry and how this coupling influences Li^+^ transport.

**Fig. 3:**
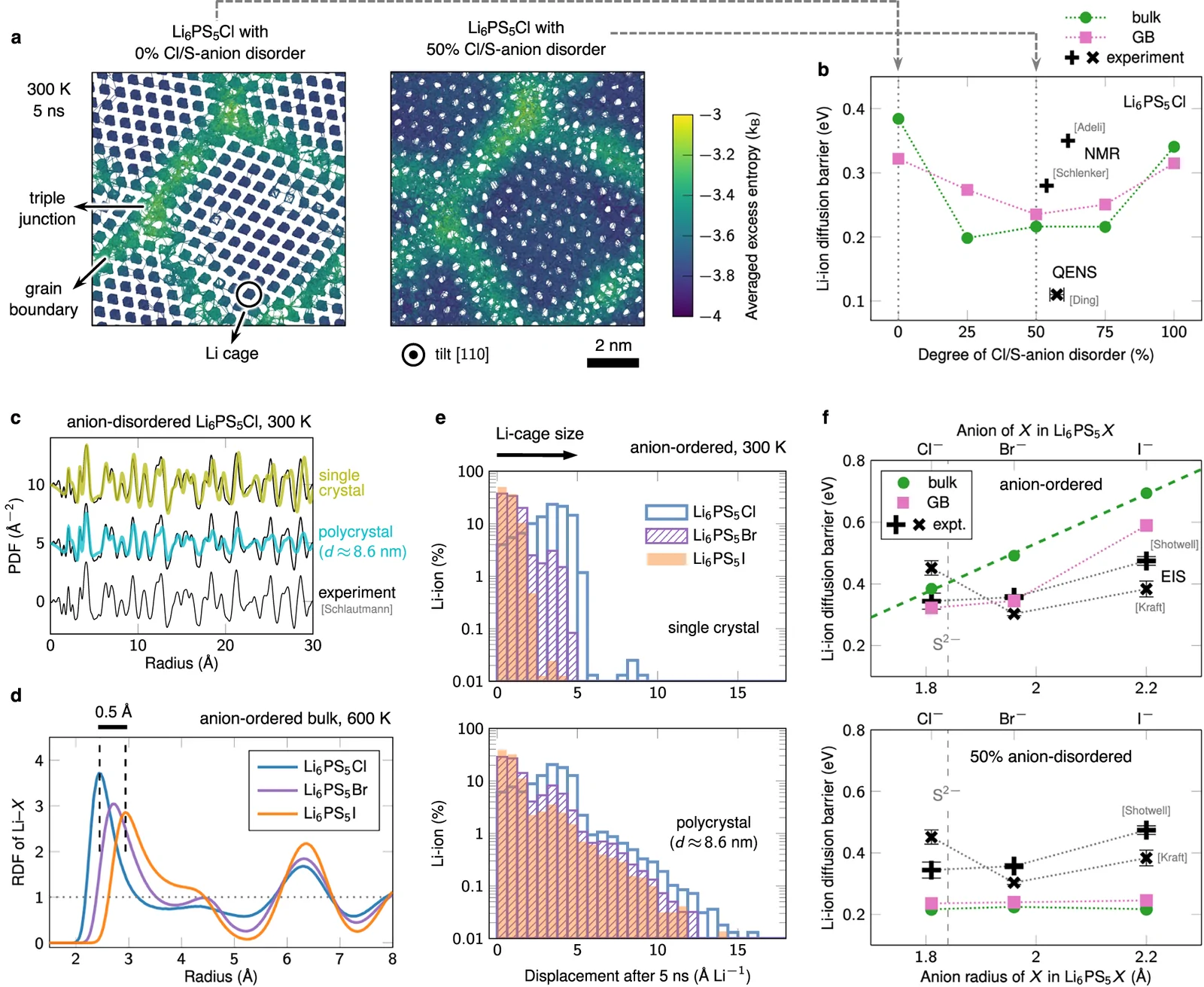
GB effects in argyrodite solid electrolytes with anion disorder and halide substitution. **a** Li-ion trajectories in the columnar polycrystal of Li_6_PS_5_Cl from MD at 300 K over 5 ns. GBs are characterized by increased excess entropy, indicating lattice distortions. **b** Li-ion diffusion barriers in bulk and GBs (averages of all GB structures in the polycrystals) in Li_6_PS_5_Cl. Measurements based on nuclear magnetic resonance (NMR; Adeli et al.^72^, Schlenker et al.^73^) and quasielastic neutron scattering (QENS; Ding et al.^69^) show higher and lower barriers, respectively. **c** Comparison of simulated and experimental pair distribution functions (PDFs) for Li_6_PS_5_Cl. Simulation results for single-crystalline (*y*-axis offset: +10 Å^−2^) and polycrystalline (grain size  ~8.6 nm; +5 Å^−2^) structures with 50% anion disorder equilibrated at 300 K are shown. Experimental data from Schlautmann et al.^74^ are shown with offsets for comparison. The simulated bulk PDF matches the experimental PDF, whereas the nanocrystalline PDF shows weaker peaks beyond 15 Å, reflecting reduced structural coherence. **d** Time-averaged Li^+^–halide radial distribution functions (RDFs) of bulk Li_6_PS_5_*X* obtained from MD at 600 K. The first Li^+^–halide coordination peak in Li_6_PS_5_I is attenuated and at a longer distance (~+0.5 Å) compared to that in Li_6_PS_5_Cl. Li_6_PS_5_Cl exhibits a more attenuated Li–*X* RDF than others beyond 4 Å. **e** Distribution of Li-ion displacements in anion-ordered Li_6_PS_5_*X* with *X* ∈ {Cl, Br, I} after MD at 300 K over 5 ns. Li_6_PS_5_Cl and Li_6_PS_5_Br exhibit intra-cage diffusion, whereas Li_6_PS_5_I shows primarily local hopping. GBs enhance Li-ion diffusion in all polycrystalline argyrodites, and the increase is more pronounced in Li_6_PS_5_Cl, where more Li ions show inter-cage diffusion. **f** Decoupling anion ordering, anion species, and GB effects on Li-ion diffusion barriers. For anion-ordered argyrodites, the diffusion barrier increases linearly with anion radius in the bulk and GBs generally enhance diffusion. For anion-disordered argyrodites, the diffusion barrier remains low and shows no clear dependence on anion radius. The predicted trends agree with the experimental values and errors of Shotwell et al.^30^, but differ from those of Kraft et al.^27^; both were obtained via electrochemical impedance spectroscopy (EIS).

To reveal GB effects in anion-disordered argyrodites, we employ Li_6_PS_5_Cl as the model system. Columnar and polycrystalline models are constructed from anion-disordered bulk, so their GB regions are influenced by the imposed disorder. Li-ion trajectories in the bulk from MD simulations at 300 K no longer exhibit distinct cages, indicating a homogeneous spatial distribution of diffusing Li ions (cf. Fig. 3a). Anion disorder lowers the bulk diffusion barrier by  ~0.17 eV (0% vs. 50%; Fig. 3b), consistent with previous studies^24,25^. GBs increase the excess entropy in both anion-ordered and anion-disordered bulk phases, yet their influence on Li-ion diffusion differs. With ordered anions, the diffusion barrier at GBs is reduced by  ~80 meV, whereas with 50% disorder, GBs increase the barrier by  ~10 meV relative to the bulk.

At 300 K, GBs in anion-disordered Li_6_PS_5_Cl reduce Li-ion self-diffusivity by  ~60% relative to the bulk yet retain high characteristic frequencies (~10^8^ Hz; Table 1), corroborating the measured low GB resistance^26^ and indistinguishable GB contributions in impedance spectra^27^. Predicted bulk and GB diffusion barriers (Fig. 3b;  ~0.23 eV) and room-temperature diffusivities (Supplementary Fig. 10;  ~10^−7 ^cm^2^ s^−1^) fall within the range of experimental values obtained by different methods (0.11 to 0.37 eV; 3 × 10^−8^ to 7 × 10^−7 ^cm^2^ s^−1^). This suggests that GBs are not the primary source of the large experimental variations, which may instead arise from other defects^28^ or spatially inhomogeneous anion disorder. Experimental pair distribution functions closely match predictions for bulk Li_6_PS_5_Cl (Fig. 3c), implying that the measured samples are dominated by micrograins with bulk-like local coordinations. Nanosizing argyrodites is predicted to weaken correlations beyond 15 Å, providing an atomistic explanation for the pair distribution decay observed in experiments^29^.

**Table 1:** Predicted Li-ion self-diffusivities and derived quantities of argyrodite solid electrolytes at 300 K

System	Li_6_PS_5_Cl					Li_6_PS_5_Br	Li_6_PS_5_I
Anion disorder (%)	0	25	50	75	100	50	0
*D*^bulk^ (10^−7^ cm^2^ s^−1^)	0.010	2.153	2.184	1.808	0.048	1.522	6.2 × 10^−7^
*D*^GB^ (10^−7^ cm^2^ s^−1^)	0.092	0.401	0.959	0.690	0.136	0.436	3.7 × 10^−5^
*D*^GB^/*D*^bulk^	8.79 *↑*	0.19 *↓*	0.44 *↓*	0.38 *↓*	2.85 *↑*	0.29 *↓*	59.25 *↑*
*f*^bulk^ (Hz)	8.61 × 10^6^	1.77 × 10^9^	1.80 × 10^9^	1.49 × 10^9^	3.92 × 10^7^	1.25 × 10^9^	5.05 × 10^2^
*f*^GB^ (Hz)	7.57 × 10^7^	3.30 × 10^8^	7.90 × 10^8^	5.68 × 10^8^	1.12 × 10^8^	3.57 × 10^8^	2.99 × 10^4^
*f*^experiment^ (Hz) _[Shotwell]_		2.50 × 10^7^				1.37 × 10^7^	1.27 × 10^5^

The anion disorder row indicates the degree of *X*/S-anion disorder. Diffusion coefficients *D*^GB^ are from MD simulations of polycrystals, representing averages over all GB structures within them. The GB-to-bulk ratio quantifies the impact of GBs on diffusion in the polycrystal with *↑* and *↓* indicating increase and decrease, respectively. The *f* rows show the predicted characteristic Li-ion hopping frequencies, which are consistent with experimental values from Shotwell et al.^30^ Experimental samples of Li_6_PS_5_Cl and Li_6_PS_5_Br exhibit about 23% and 15% anion disorder, respectively, whereas Li_6_PS_5_I shows less than 5% disorder.^30^ Characteristic frequencies of the bulk and GBs are comparable for anion-disordered Li_6_PS_5_Cl and Li_6_PS_5_Br, but differ by about two orders of magnitude for anion-ordered Li_6_PS_5_I.

We examine the effects of anions on Li-ion diffusion using polycrystalline models built from anion-ordered phases and varying the anions from Cl^−^ to Br^−^ or I^−^. Radial distribution functions of the bulk, obtained from MD at 600 K, are used to probe the coordination environment between Li-ion and anions (Fig. 3d). Compared with Li–Cl, the first peak of the Li–I radial distribution function is shifted outward by  ~0.5 Å and attenuated. The increase in Li–*X* bond length reflects the larger anion radius of I^−^, while the first-peak softening may result from weaker interactions of Li–I vs. Li–Cl. Apart from the first peak, other peaks in Li–I are sharper than in Li–Cl, indicating that the bulk lattice is more ordered in Li_6_PS_5_I than in Li_6_PS_5_Cl during MD simulations.

Li-ion displacements in single-crystalline anion-ordered phases after 5 ns of MD at 300 K are analyzed (Fig. 3e). In the Li_6_PS_5_Cl bulk, most Li-ion displacements match the Li-cage size (~5 Å), confirming intra-cage motion, whereas in Li_6_PS_5_I, displacements are mostly limited to  ~3 Å, reflecting local hopping between adjacent Li-ion sites. Simulations predict that substituting Cl with I in bulk argyrodites increases the diffusion barrier by  ~0.3 eV. Including Li_6_PS_5_Br reveals a clear linear scaling of Li-ion diffusion barrier with anion radius (Fig. 3f), with an increase of 0.1 Å leading to an  ~80 meV higher barrier. A linear trend also holds for GB formation energy (Supplementary Fig. 18). By contrast, this trend is not apparent in anion-disordered argyrodites.

Our systematic study enables decoupling the effects of anion ordering, anion species, and GBs. In anion-ordered phases, GBs disrupt Li-ion cages in all Li_6_PS_5_*X*, leading to Li-ion displacements exceeding 5 Å. These effects are anion-dependent. Diffusion is most enhanced at Br GBs, which reduce the diffusion barrier by nearly 0.2 eV, compared with reductions of less than 0.1 eV in the Cl and I systems. For anion-ordered Li_6_PS_5_I, experimental values are more than 0.1 eV lower than the predicted GB value, likely due to additional defects that further enhance Li-ion transport^28,30^. By contrast, in anion-disordered phases, GBs introduce a nearly uniform additional barrier of  ~0.02 eV.

### Non-Arrhenius transport and grain-size effects

**Fig. 4:**
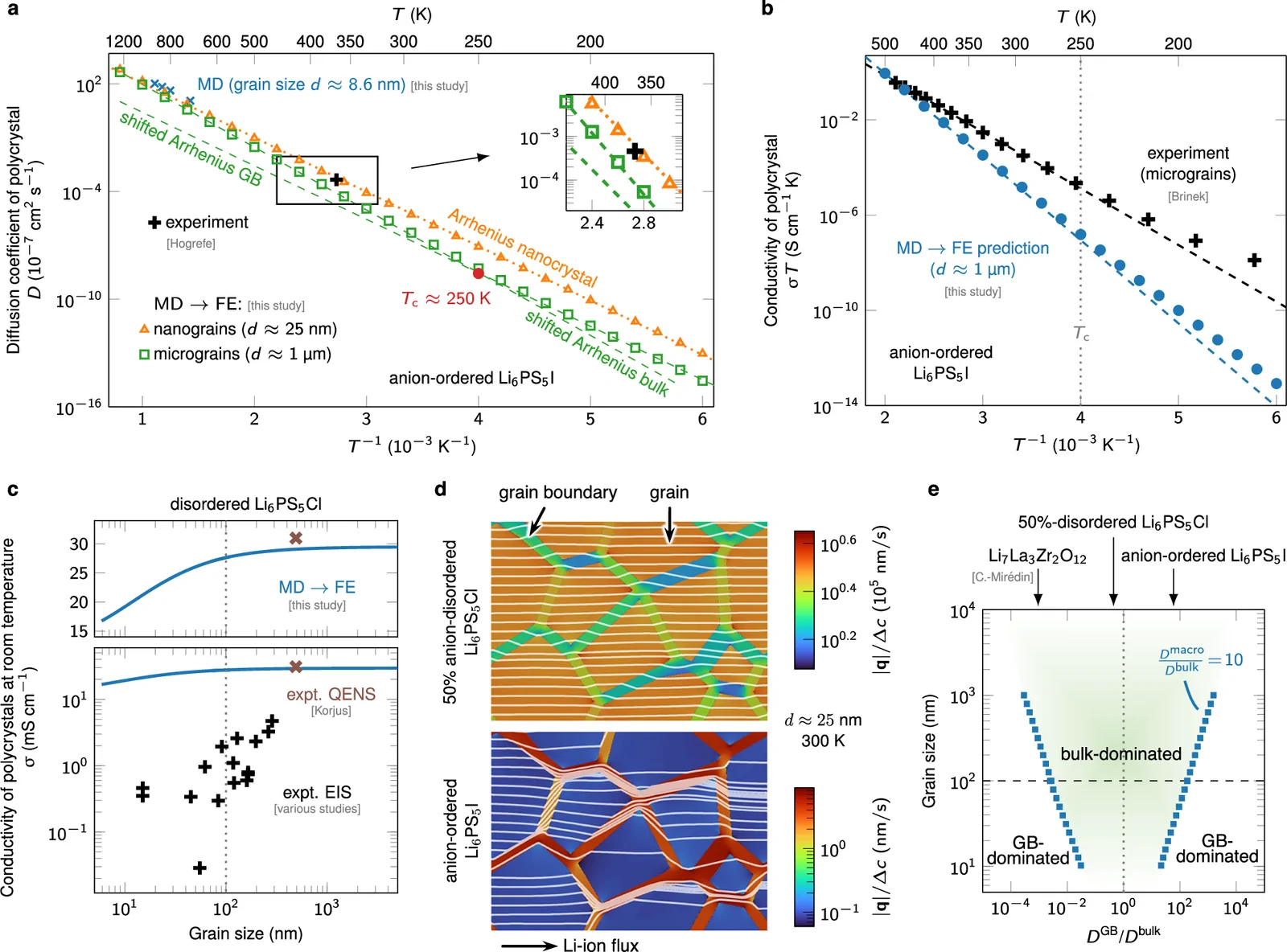
Li-ion transport from atomic-scale diffusion to continuum-scale percolation. **a** Comparison of predicted and experimental diffusivities of anion-ordered Li_6_PS_5_I. MD simulations are limited to small grain sizes and high temperatures, while the proposed approach (MD  → FE) provides accurate predictions for wide ranges of grain size and temperature. Two diffusion regimes are identified from shifted Arrhenius fits of the calculated bulk and GB diffusivities, which also determine the transition temperature. Predicted macroscopic diffusivities are in good agreement with nuclear magnetic resonance measurements (Hogrefe et al.^68^). **b** Non-Arrhenius transport behavior in anion-ordered polycrystalline Li_6_PS_5_I originating from GBs. Predicted and experimental transition temperatures (*T*_c_ ≈ 250 K; Brinek et al.^43^) and low-temperature activation energy changes (−0.10 vs. −0.12 eV) are in good agreement. **c** Grain-size effect on conductivities of anion-disordered Li_6_PS_5_Cl at room temperature. Predictions for Li_6_PS_5_Cl with 50% disorder show good agreement with quasielastic neutron scattering (QENS) measurements (Korjus et al.^67^), and exceed values from electrochemical impedance spectroscopy (EIS) measurements (various studies^75–79^). **d** Two-dimensional projections of the Li-ion flux **q** at 300 K for nanocrystalline (grain size  ~25 nm) Li_6_PS_5_Cl with 50% disorder (*D*^GB^ < *D*^bulk^) and ordered Li_6_PS_5_I (*D*^GB^ > *D*^bulk^). The fluxes are obtained from FE simulations under an applied horizontal concentration jump Δ*c*. **e** Conditions under which GB effects substantially influence the diffusivity in polycrystalline solid electrolytes. The cases where GBs alter the macroscopic diffusivity by more than an order of magnitude are referred to as GB-dominated, while bulk-dominated refers to the opposite. The GB-to-bulk diffusivity ratios of 50% anion-disordered Li_6_PS_5_Cl and anion-ordered Li_6_PS_5_I from the present study and of Li_7_La_3_Zr_2_O_12_ from C.-Mirédin et al.^6^ at 300 K are indicated.

Atomistic simulations provide mechanistic insights into Li-ion diffusion at GBs, but with limitations (cf. MD results in Fig. 4a). Simulations are limited to grain sizes of about 10 nm and only a few GBs, making macroscopic properties of real materials inaccessible. Time scales are also limited to nanoseconds, much shorter than in experiments. To predict the macroscopic properties of polycrystalline solid electrolytes with micrometer-sized grains while retaining near-ab initio accuracy, we utilize the MD-derived diffusivities and GB width (Supplementary Note 4) in continuum FE simulations for the three anion-ordered Li_6_PS_5_*X* with *X* ∈ {Cl, Br, I} and for Li_6_PS_5_Cl with varying ratios of anion disorder.

For Li_6_PS_5_I with micrograins (Fig. 4a), the macroscopic diffusivity exhibits non-Arrhenius behavior, with a transition temperature around 250 K. Diffusion is dominated by the bulk at high temperatures, with a barrier near 0.69 eV, and by GBs at low temperatures, with a barrier near 0.59 eV. The transition temperature and the degree of non-Arrhenius behavior are halogen-dependent (Supplementary Note 5). Reducing the grain size to the nanometer scale mitigates non-Arrhenius behavior. Simulation-predicted grain-size effects suggest that the experimentally measured diffusivity (4.6 × 10^−11 ^cm^2^ s^−1^) corresponds to a polycrystal with an effective grain size of 31 nm (Supplementary Fig. 20). We convert the diffusivities of a polycrystal with grain sizes about 1 μm into conductivities (see “Methods”; Fig. 4b). Predictions from simulations agree with the experimentally observed non-Arrhenius transport behavior. Although the predicted conductivities are underestimated—likely due to an overestimation of the diffusion barrier for Li_6_PS_5_I (Fig. 3f)—the transition temperature is well reproduced. This suggests that the non-Arrhenius behavior observed in experiments may originate from GBs in the samples.

Experimentally, the grain-size effect in argyrodites remains unclear, as grain size is difficult to control and conductivity varies with synthesis (cf. Fig. 4c). We predict the macroscopic conductivity of Li_6_PS_5_Cl with 50% anion disorder at 300 K. Conductivity approaches the bulk value as grain size increases, with the most significant changes occurring with nanograins. Good agreement is observed between predictions and quasielastic neutron scattering measurements. In contrast, the predicted values are roughly an order of magnitude higher than the highest experimental values measured by electrochemical impedance spectroscopy, which probes macroscopic transport. This discrepancy is partially mitigated by considering voids in solid electrolytes (Supplementary Fig. 22). Additional discrepancy may arise from differences in density, pellet processing, or electrode–sample contact^31^. Therefore, the predictions provide grain-size-dependent upper limits for the conductivities of Li_6_PS_5_Cl. To examine the impact of nanosized grains in solid electrolytes, Li-ion fluxes in disordered Li_6_PS_5_Cl and ordered Li_6_PS_5_I at 300 K are visualized using FE simulations (Fig. 4d). In Li_6_PS_5_Cl, GBs show little effect on flux magnitude or orientation, whereas in Li_6_PS_5_I, GBs act as connecting channels for Li-ion transport, with Li-ion preferentially flowing through them. These results reflect the impact of GBs on Li-ion transport: They reduce diffusivity by half relative to the bulk in Li_6_PS_5_Cl, but enhance diffusivity by up to 60-fold in Li_6_PS_5_I (Table 1).

The impact of GBs on macroscopic diffusivity or conductivity depends on both *D*^GB^ and grain size. We provide a precomputed map linking atomic-scale changes in diffusivity to continuum-scale effects based on FE simulations (Supplementary Fig. 21). Using the criterion that GBs alter the total diffusivity by more than an order of magnitude, the bulk-dominated and GB-dominated regimes can be quantitatively estimated (Fig. 4e). For solid electrolytes with micrograins (*d* > 100 nm), the GB effect becomes significant when GB diffusivity is either two orders of magnitude higher or three orders of magnitude lower than the bulk.

## Discussion

Our simulations reveal that GB voids are subnanometer in size and that GBs do not significantly impede Li^+^ diffusion in the Li_6_PS_5_Cl solid electrolyte. The lower-than-predicted experimental conductivities likely stem from limited interparticle contacts in powder samples, even after processing^31^. Grain refinement, such as wet milling before mechanical pressing^32^, which reduces porosity and introduces GBs, can thus effectively enhance intergranular contact in sulfide solid electrolytes while preserving their superionic conductivity.

GBs in argyrodites exert markedly different effects depending on the underlying anion sublattice. In materials with fast bulk diffusion pathways, GBs act as barriers, consistent with the reported GB resistance in oxide-based solid electrolytes^7,33^. Reducing GB resistance through modification or minimizing GB density is essential to improve such materials^7^. In contrast, in systems with intrinsically low bulk diffusivity, GBs facilitate Li^+^ transport, as exemplified by anion-ordered Li_6_PS_5_I. Similar GB-induced diffusion enhancement has also been predicted in sulfides such as Na_3_PS_4_^34^ and *β*-Li_3_PS_4_^17^, in the oxide LiZr_2_(PO_4_)_3_^35^, and in the halide Li_3_InCl_6_^13^. Consequently, nanosizing can offer a strategy to improve potential solid electrolytes lacking intrinsic superionic conductivity.

Because experimentally controlling anion type and anion disorder independently is difficult, the impact of incorporating different anions in argyrodites on their intrinsic transport properties has been unclear^30,36^. Our results reveal a linear dependence between diffusion barrier and the anion radius in ordered argyrodites. A large radius mismatch between S^2−^ and *X*^−^ obstructs Li^+^ pathways and increases the diffusion barrier, consistent with the observation for K_3_O*X*^37^. This trend contrasts with LISICON-type solid electrolytes, where increasing the cation radius lowers the diffusion barrier to as low as 0.1 eV^38^. Our finding indicates that lattice polarizability, tunable through halide substitution, may not be the dominant factor governing Li-ion diffusivity in argyrodites, contrary to earlier suggestions^27^.

Non-Arrhenius transport behavior has been reported for several solid electrolytes and attributed to local energy fluctuations within the bulk^39,40^. However, this explanation cannot hold for Li_6_PS_5_I, where the diffusion barrier decreases at low temperature^36^, with a transition at 250 K—well above the structural transition observed experimentally at 160 K^36,41^. The simulations show that GBs can give rise to non-Arrhenius behavior in Li_6_PS_5_I, thereby providing a possible atomic-scale mechanism for the experimental observations^36^. Our results for Li_6_PS_5_*X* reveal that both the transition temperature and the magnitude of non-Arrhenius behavior are halogen-dependent (Supplementary Note 5), consistent with the softened and ultimately absent anomaly observed for Li_6_PS_5_Br_0.5_I_0.5_ and Li_6_PS_5_Br, respectively^36^. In halide-rich argyrodites, GBs hinder Li^+^ transport, leading to a predicted increase in the low-temperature diffusion barrier, consistent with experiments^42^. Moreover, our results reveal that nanosizing suppresses the non-Arrhenius response of Li_6_PS_5_I, in line with experiments^43^. Because non-Arrhenius behavior near room temperature can destabilize battery performance, it can be practically mitigated by controlling grain size or tuning GB diffusivity.

In conclusion, multiscale modeling integrating molecular dynamics and finite element simulations is accurate and efficient for studying ion transport in polycrystalline solid-state electrolytes. This approach provides the atomic-to-macroscopic support for the experimentally observed non-Arrhenius Li^+^ transport in argyrodites and offers insights for designing solid electrolytes in all-solid-state batteries. Grain boundaries (GBs) can enhance or suppress diffusion depending on bulk diffusivity, implying that nanosizing may activate transport in systems lacking intrinsic superionic behavior. For Li_6_PS_5_Cl, grain refinement can improve intergranular contacts without compromising conductivity. Our results also highlight the anion radius as a key compositional parameter. Looking forward, systematic screening of GB diffusivities can establish the basis for extending GB engineering to solid electrolyte interfaces.

## Methods

### Multiscale modeling framework

Machine-learning potentials for large-scale atomistic simulations should balance accuracy with computational cost. To obtain such potentials for argyrodite solid electrolytes, a closed-loop active learning scheme combined with systematic parameterization of moment tensor potentials (MTPs) is developed.

First, given that structural and chemical complexities are concentrated in grain-boundary (GB) regions, local atomic environments are extracted from full structures in the active learning process (Supplementary Fig. 4a). The extraction of local structures substantially reduces the cost of density-functional theory (DFT) calculations. The preservation of stoichiometry of the extracted structure and the relaxation of atoms at the boundaries of the periodic cell are introduced to minimize artificial effects.

Second, a quality-level-based scheme is employed to efficiently sample the configurational space (Supplementary Fig. 4b). Initial potentials with a small number of fitting parameters are trained on short ab initio molecular dynamics (MD) trajectories and used to explore configuration space via active learning. The accuracy of the potential after active learning is evaluated, and if insufficient, a potential with more fitting parameters (more accurate and computationally intensive) is used for the next round of active learning. This iterative process continues with increasingly complex potentials, enabling systematic and data-efficient development of potentials for complex structures requiring ab initio accuracy.

The final machine-learning potentials are optimized for both accuracy (Supplementary Fig. 5) and efficiency (Supplementary Fig. 6), guided directly by the target material properties rather than fitting or validation error. We refer readers to Supplementary Note 1 for further details.

Finite element (FE) simulations are employed to extend the accessible timescales from nanoseconds to beyond microseconds and length scales from nanometers to polycrystalline systems with micrometer-sized grains (Supplementary Fig. 4c). The statistically converged diffusivities obtained from large-scale MD simulations accelerated by the trained MTP, including the GB width parameters, serve as input for the continuum-scale FE simulations.

Resolving GB layers in polycrystalline models presents computational challenges for mesh-based FE simulations. To address this, different approaches are implemented depending on grain size to balance the accuracy and computational efficiency. For nanocrystalline polycrystals, GBs are volume-resolved, and continuum diffusion equations are solved using fast Fourier transform (FFT)–based methods. For microcrystalline polycrystals, GB layers become relatively thin and are thus represented as thickness-collapsed finite elements. In both cases, diffusivity tensors of polycrystalline models are obtained through linear computational homogenization. Comparative tests reveal that the volume-resolved and thickness-collapsed GB representations complement each other, supporting their combined use as an effective strategy to address the challenges of length-scale separation and diverse kinetic regimes presented in polycrystalline solid electrolytes (Supplementary Fig. 11). We refer readers to Supplementary Note 3 for further details.

The proposed multiscale modeling framework enables prediction of macroscopic diffusivities in polycrystalline solid electrolytes up to device-relevant scales (Supplementary Fig. 4d). Its main strength lies in balancing accuracy and efficiency across the entire modeling process, through an optimized active learning scheme for machine-learning potentials, a data-efficient multiscale workflow, and tailored methods for resolving GBs in FE simulations. Moreover, our modeling framework can be readily extended to explicitly include additional microstructural features. For example, surface diffusivities obtained from atomistic simulations can be incorporated into continuum models to describe transport along pore surfaces, enabling multiscale modeling of porous polycrystalline solid electrolytes.

### Active learning

In the MTP formalism^44^, the total energy *E* of a structure is the sum of the atomic energies $V({{\mathfrak{n}}}_{i})$ of each atom in the structure,

1 $$E={\sum}_{i=1}^{N}V({{\mathfrak{n}}}_{i}),$$

where ${{\mathfrak{n}}}_{i}$ is the local atomic environment of atom *i*, and *N* is the number of atoms in the structure.

Since the atomic energy given by an MTP has a non-linear dependence on its fitting parameters, the generalized D-optimality criterion and the MaxVol algorithm^45,46^ based on the fitted MTPs are used for active learning (AL). In AL, the term global-AL refers to cases where uncertainty is quantified over the entire simulation model, whereas local-AL refers to cases where uncertainty is quantified individually for each atom in the model. For global-AL, variables *B*_*jk*_ are defined as the partial derivative of the total energy *E*_*j*_ of an atomic structure *j* predicted by the fitted MTP and the fitting parameters *θ*_*k*_ of the MTP:

2 $${B}_{jk}:=\frac{\partial {E}_{j}}{\partial {\theta }_{k}},\quad {{{\rm{for}}}}\,{{{\rm{global}}}}\,{{{\rm{AL}}}}.$$

In local-AL, variables *B*_*jk*_ are defined as the partial derivative of the atomic energy $V({{\mathfrak{n}}}_{j})$ of a local atomic environment ${{\mathfrak{n}}}_{j}$ predicted by the fitted MTP and the fitting parameters *θ*_*k*_ of the MTP:

3 $${B}_{jk}:=\frac{\partial V({{{\mathfrak{n}}}}_{j})}{\partial {\theta }_{k}},\quad {{{\rm{for}}}}\,{{{\rm{local}}}}\,{{{\rm{AL}}}}.$$

Suppose that an MTP has *m* fitting parameters and has been fitted to a training set, i.e., the fitting parameters are optimized or initialized. For the MTP, an active set with *m* elements (*m* structures for global-AL or *m* local atomic environments for local-AL) can be selected from the training set so that they maximize the absolute value of the determinant of the following square matrix:

4 $${{{\bf{A}}}}=\left[\begin{array}{cccc}{B}_{11}&{B}_{12}&\cdots \,&{B}_{1m}\\ {B}_{21}&{B}_{22}&\cdots \,&{B}_{2m}\\ \vdots &\vdots &\ddots &\vdots \\ {B}_{m1}&{B}_{m2}&\cdots \,&{B}_{mm}\\ \end{array}\right].$$

In the active set matrix **A**, each row corresponds to an element, and each column corresponds to the partial derivative of a fitting parameter.

For a specific structure or local atomic environment *x*, the structural descriptor vector of *x* can be obtained based on a fitted MTP,

5 $${{{{\bf{B}}}}}_{x}=\left({B}_{x1},{B}_{x2},\cdots \,,{B}_{xm}\right).$$

Together with the active set matrix **A** of the fitted MTP, the extrapolation grade^44^ of *x* can be calculated as

6 $${\gamma }_{x}=\max | {{{{\bf{B}}}}}_{x}{{{{\bf{A}}}}}^{-1}| .$$

It can be interpreted that, given the training set and the fitted MTP, *x* is evaluated as interpolative if *γ*_*x*_ < 1 and as extrapolative if *γ*_*x*_ > 1^47^.

In the present study, MTPs with eight radial basis functions were used. The minimum and maximum cutoffs were set to 1.5 and 5 Å, respectively. The fitting weights for the energies and atomic forces were set to ${N}_{{{{\rm{at}}}}}^{-1}$ and 0.001 Å^2^, respectively, where *N*_at_ is the number of atoms in the corresponding cell. The local extrapolation grade thresholds for selecting and breaking were 1.4 and 5, respectively. MTPs trained up to level 18 were used for all simulation models in the production runs. For the same material system, the training and validation root-mean-squared errors of all utilized MTPs are below 10 meV atom^−1^ in energy (Supplementary Fig. 5). Details of the parameters used in AL are provided in Supplementary Note 1 and Supplementary Figs. 1–3.

### Simulation models

The bicrystal GB models were constructed based on the ideal anion-ordered bulk of Li_6_PS_5_Cl and coincidence-site lattice theory^48^ following our previous study^22^. The geometry of a GB structure can be labeled by the integer index Σ, which is defined as the ratio between the coincidence unit cell volume and the rotated unit cell volume^49^. The rotation axis of the two grains in the bicrystal models was set along [110], while the rotation angle between them was varied to generate distinct GB configurations. Supercells were constructed to ensure that adjacent periodic GBs were separated by more than 50 Å. The grains were slightly shifted in the normal direction of the GB plane to ensure that atomic distances of all atom pairs are larger than 1.8 Å.

The atomistic columnar crystal and polycrystalline models consist of two grains in cubic cells and were constructed using ATOMSK^50^ with periodic boundary conditions applied. For the columnar crystal, the rotation axis is [110] and the misorientation angle is  ~61°. The position and crystallographic orientations of the two grains in the polycrystalline models were randomized, with the resulting misorientation angles  ~44°, ~6°, and ~8° for the rotation axes [100], [010], and [001], respectively. The resulting atomic models thus contain distinct, randomly oriented GBs, serving as representative surrogate GB models. For each grain in the columnar and polycrystalline models, the stoichiometry of elements was ensured by removing atoms at GB regions. To construct anion-disordered models, S atoms inside the Li cage and Cl atoms were randomly selected according to the disorder percentage and exchanged, following our previous study^22^. All simulation cells maintain the stoichiometry of Li_6_PS_5_*X*, ensuring formal charge neutrality. The local entropy of atomistic models was calculated based on the two-body excess entropy^51^ implemented in OVITO^52^. Atomistic models were visualized with OVITO and VESTA^53^. Pair distribution functions were calculated using DIFFPY-CMI^54^.

Polycrystalline microstructures in the continuum simulations were generated using three-dimensional periodic Voronoi tessellations^55^ within a unit-cube domain, providing a simplified representation of realistic GB networks^56^. This approach is appropriate for the present study, as the GB diffusivity averaged over all GBs in the surrogate atomic model was used as input for the continuum simulations. The approach remains justified even when distinct GB diffusivities are used in the FE simulations (Supplementary Fig. 20a), as the variation in diffusivities across different GB types is small. The same tessellations were employed for both volume-resolved and thickness-collapsed GB representations, ensuring that the two approaches remain directly comparable. The GB width was set to 2.5 nm, consistent with MD-resolved atomic structures (Supplementary Note 4).

In continuum simulations, different discretizations were applied for the two GB representations (Supplementary Fig. 4c). To resolve the GB, a structured voxel grid of prescribed resolution was generated using MSUTILS (see “Code availability”), and each voxel was assigned an MD-derived diffusivity according to its region in the polycrystalline model. In the collapsed approach, a GB-conforming second-order mesh was generated with NEPER^57,58^ and then enriched with the interface elements^18^. The GB thickness was treated as an implicit parameter of the constitutive model, without being explicitly represented in the discretization.

Continuum simulations in production runs were carried out on a nearly isotropic polycrystalline model containing 27 grains, considered a realistic representative volume element (RVE) for polycrystalline solid electrolytes. The consistency of the two GB representations was tested using an idealized two-grain polycrystalline model to reduce computational cost. Symmetrically placing the two tessellation seeds produces diamond-shaped grains, and the resulting model possesses isotropic transport properties. Voxel-based images of the resolved geometry and the corresponding collapsed crinkle-cut geometry for both the realistic and idealized models are shown in Supplementary Fig. 11a, b. The continuum simulation models were visualized using ParaView^59^. Schematic figures were created using Microsoft PowerPoint.

### Ab initio and atomistic simulations

Electronic-structure calculations were performed using DFT as implemented in VASP^60–62^. The projector augmented-wave method^63^ and the generalized gradient approximation in the Perdew–Burke–Ernzerhof (PBE) parametrization^64^ were used. Electrons in the atomic orbitals 1*s*^2^2*s*^1^, 3*s*^2^3*p*^3^, 3*s*^2^3*p*^4^, 3*s*^2^3*p*^5^, 4*s*^2^4*p*^5^, and 5*s*^2^5*p*^5^ were treated as valence electrons for Li, P, S, Cl, Br, and I, respectively. The plane-wave cutoff was set to 500 eV, and the energy was converged to less than 10^−3^ eV per simulation cell. Ab initio MD simulations were performed with the conventional unit cell of Li_6_PS_5_*X* with *X* ∈ {Cl, Br, I}, containing 52 atoms, at 1500 K using the Nosé–Hoover thermostat and the canonical ensemble implemented in VASP. The time step and the Nosé mass were set to 2 fs and 3 u Å^2^, respectively. The reciprocal space was sampled using the Gaussian smearing with a width of 0.03 eV and a Gamma-centered 2 × 2 × 2 **k**-point mesh (416 kp atom). For electronic-structure calculations of the extracted structures with about 200 atoms, the reciprocal space was sampled at the Gamma point.

Large-scale MD simulations were performed by LAMMPS^65^ with the fitted MTPs, with a time step of 2 fs. The energy of the relaxed structure was obtained by the MD-based annealing-and-quenching approach: The GB-containing atomic models were first equilibrated at 600 K, then cooled to near 0 K over 1 ns, and finally relaxed to 0 K^22^. The GB energy *Γ* was calculated by

7 $$\varGamma=\frac{{E}_{{{{\rm{GB}}}}}-{E}_{{{{\rm{bulk}}}}}}{{A}_{{{{\rm{tot}}}}}},$$

where *E*_GB_ and *E*_bulk_ are the energies of the relaxed models with GB and with only bulk, respectively, and *A*_tot_ is the total GB area in the simulation cell. For the MD simulations targeting diffusion properties, the structures were first equilibrated with the *NPT* ensemble at zero pressure for 0.2 ns. The self-diffusion coefficient *D* at temperature *T* was calculated by

8 $$D(T)=\frac{{\langle {u}^{2}\rangle }_{T}}{2dt},$$

where ${\langle {u}^{2}\rangle }_{T}$ is the mean square displacement of Li atoms in MD, and *d* and *t* are the number of dimensions and simulation time, respectively. The production runs were performed in the *NVE* ensemble for 5 ns, and the simulation window from 3 to 5 ns was used to calculate diffusion coefficients.

GB diffusion coefficients were extracted from the diffusion coefficients derived from the simulation model with GBs. A GB width of 2.5 nm was used, consistent with our previous study^22^. Within the normal diffusion regime, an Arrhenius relation for diffusion coefficients can be defined as

9 $$D(T)={D}_{0}\exp \left(-\frac{{E}_{{{{\rm{a}}}}}}{{{{{\rm{k}}}}}_{{{{\rm{B}}}}}T}\right),$$

where *D*_0_ is a constant, *E*_a_ is the diffusion barrier, and k_B_ is the Boltzmann constant.

Ionic conductivity *σ* is related to self-diffusivity *D* via a modified Nernst–Einstein equation^66^,

10 $$ \sigma={\varLambda} \frac{{(z{{{\rm{e}}}})}^{2}D}{{{{{\rm{k}}}}}_{{{{\rm{B}}}}}T},$$

where *z* is the valence of the mobile ion (*z* = 1 for Li^+^), e is the elementary charge, and *Λ* is a system-dependent coefficient related to the concentration of the mobile ions and the Haven ratio. For Li_6_PS_5_Cl, *Λ* ≈ 2.181 × 10^−2 ^Li Å^−3^ at 298 K was used, following our previous study^22^. The same *Λ* was used to convert the diffusion coefficients obtained from quasielastic neutron scattering measurements in ref. ^67^. For Li_6_PS_5_I, the conductivity from ref. ^43^ and the diffusivity at 366 K from ref. ^68^ were used, resulting in *Λ* ≈ 1.590 × 10^−1 ^Li Å^−3^.

The residence time τ of inter-cage Li-ion jumps was estimated as

11 $$\tau=\frac{{l}^{2}}{6D},$$

where *l* denotes the inter-cage jump distance. The corresponding characteristic frequency *f* was then obtained as

12 $$f=\frac{1}{\tau }.$$

The inter-cage jump distance of anion-disordered Li_6_PS_5_Cl was reported to be 2.70 Å^69^. Given the similar radial distribution functions, the same value (2.70 Å) was adopted for the anion-ordered phases and GBs. The relaxed lattice constants of the anion-ordered Li_6_PS_5_Cl, Li_6_PS_5_Br, and Li_6_PS_5_I structures (cubic, $F\bar{4}3m$ symmetry) were calculated as 10.28 Å, 10.31 Å, and 10.35 Å, respectively. Assuming linear scaling with lattice parameter, the corresponding jump distances for Li_6_PS_5_Br and Li_6_PS_5_I were approximated as 2.71 Å and 2.72 Å.

### Continuum simulations

On the continuum scale, we considered an ion-concentration field *c*(**x**) defined at every position **x** within the simulation domain. The relation between the ion-concentration field and the ion flux **q** was described by classical Fickian diffusion,

13 $${{{\bf{q}}}}({{{\bf{x}}}})=\left\{\begin{array}{ll}-{{{{\bf{D}}}}}^{{{{\rm{bulk}}}}}\nabla c({{{\bf{x}}}})\quad &\,{{\rm{in}}}\,\,\,{{\rm{bulk}}}\,,\\ -{{{{\bf{D}}}}}^{{{{\rm{GB}}}}}\nabla c({{{\bf{x}}}})\quad &\,{{\rm{in}}}\,\,\,{{\rm{GB}}}\,,\end{array}\right.$$

where **D**^bulk^ and **D**^GB^ are diffusion tensors of the bulk and GB, respectively. The bulk was assumed isotropic, i.e., **D**^bulk^ = *D*^bulk^**I**, with the scalar diffusivity *D*^bulk^ derived from atomistic simulations. GBs were defined as transversely isotropic: Diffusion within the GB plane was described by ${D}_{\parallel }^{{{{\rm{GB}}}}}$, whereas diffusion perpendicular to the plane was governed by ${D}_{\perp }^{{{{\rm{GB}}}}}$. Both ${D}_{\parallel }^{{{{\rm{GB}}}}}$ and ${D}_{\perp }^{{{{\rm{GB}}}}}$ were derived from atomistic simulations. When averaged GB diffusivities derived from atomic GB models were used, the continuum simulations assumed ${D}^{{{{\rm{GB}}}}}={D}_{\parallel }^{{{{\rm{GB}}}}}={D}_{\perp }^{{{{\rm{GB}}}}}$.

The concentration field satisfies the steady-state balance equation,

14 $$\begin{array}{r}\nabla \cdot {{{\bf{q}}}}({{{\bf{x}}}})=0,\end{array}$$

corresponding to the time-independent condition reached in the long-time limit. By imposing a concentration gradient and solving for the resulting concentration fluctuation field under periodic boundary conditions, the problem is treated using linear computational homogenization, yielding the macroscopic diffusivity tensor **D**^macro^ for the RVE on the continuum scale^18^. Since the constructed polycrystalline model is nearly isotropic, only the average eigenvalue of the diffusivity tensor is reported and denoted as *D*^macro^. This average deviates from the minimum and maximum eigenvalues by at most 3%, and typically by less than 1%. Comparisons with the analytical Maxwell–Garnett estimate^70^ for isotropic GB setups show good agreement. We emphasize that our approach extends the prediction of *D*^macro^ to polycrystalline solid electrolytes with transversely isotropic GBs.

In the resolved approach, the FFT-based homogenization solver FANS^20^ (see “Code availability”) was operated on a voxel-based image of the polycrystalline model. For production runs, a 512 × 512 × 512 voxel grid, corresponding to  ~134 million degrees of freedom, was used. In the collapsed approach, a conforming mesh was employed, with  ~0.44 million degrees of freedom for the 27-grain tessellation and  ~0.23 million degrees of freedom for the two-grain tessellation. The grain size of the polycrystalline model was defined as the diameter of a sphere with a volume equal to the average grain volume in the model. Grain-size variation in the polycrystalline model was achieved by rescaling the simulation cell size while keeping the GB width unchanged.

During the preparation of this manuscript, the authors used ChatGPT (OpenAI GPT-5 model) to improve clarity and readability. All content generated with its assistance was reviewed, edited, and verified by the authors, who take full responsibility for the final content of the published article.

## Supplementary information

Supplementary Information

Transparent Peer Review file

## Source data

Source Data

## Data Availability

The data generated in this study have been deposited in the DaRUS repository^71^. The atomic dataset includes DFT and MD input files, training data, and fitted MTPs. The continuum dataset contains the employed geometries, full-field solutions, and effective responses. Source data are provided with this paper.

## Code availability

Scripts for performing quality-level-based local active learning, generating bulk, GB, and polycrystal structures, and analyzing results have been deposited in the DaRUS repository^71^. In addition, an easy-to-use demonstrator is provided in the repository for generating voxel-based geometries, performing simulations with FANS and MSUTILS, and visualizing the simulation results.
