# Supplementary material for: Microstructural insights into fast ion transport in solid electrolytes via multiscale modeling
Source: Nat Commun. 2026 Aug 20;17:8726. doi: 10.1038/s41467-026-76216-w (PMC13493929; doi:10.1038/s41467-026-76216-w)
Supplement: Supplementary file 1 [file 41467_2026_76216_MOESM1_ESM.pdf]

Supplementary Information for
Microstructural Insights into Fast Ion Transport in Solid Electrolytes via Multiscale Modeling

Yongliang Ou,[1,2,*] Lena Scholz,[3,†] Sanath Keshav,[3] Yuji Ikeda,[1] Marvin Kraft,[4,5] Sergiy Divinski,[6] Rafael Gómez-Bombarelli,[2] Wolfgang G. Zeier,[4,5] Felix Fritzen,[3,‡] and Blazej Grabowski[1,§]

[1] *Institute for Materials Science, University of Stuttgart, Stuttgart, Germany*
[2] *Department of Materials Science and Engineering, Massachusetts Institute of Technology, Cambridge, MA, USA*
[3] *Institute of Applied Mechanics, University of Stuttgart, Stuttgart, Germany*
[4] *Institute of Inorganic and Analytical Chemistry, University of Münster, Münster, Germany*
[5] *Institute of Energy Materials and Devices (IMD), IMD-4: Helmholtz-Institut Münster, Forschungszentrum Jülich, Münster, Germany*
[6] *Institute of Materials Physics, University of Münster, Münster, Germany*

(Dated: July 1, 2026)

Contents

Suppl. Note 1. Parameterization of moment tensor potentials 2

Suppl. Note 2. Validation of moment tensor potentials 3

Suppl. Note 3. Consistency of continuum simulation models 3

Suppl. Note 4. Sensitivity to grain boundary width 5

Suppl. Note 5. Non-Arrhenius transport behavior 5

Suppl. Note 6. Additional results and discussion 5

Supplementary references 32

List of supplementary items

Table 1: Transferability of MTPs from local AL
Table 2: Examples of calculated GB volume fractions
Table 3: Study timeline of solid electrolyte GBs
Table 4: Overview of predicted diffusivities

Fig. 1: Quality-level-based MTP training scheme
Fig. 2: Complete iterative MTP training workflow
Fig. 3: Steps and hyperparameters of local AL
Fig. 4: Overview of multiscale modeling framework
Fig. 5: Accuracy of local active learning
Fig. 6: Efficiency of local active learning
Fig. 7: Training errors for single-type GBs
Fig. 8: Validation errors for single-type GBs
Fig. 9: Comparison of MTPs to ab initio results
Fig. 10: Li_6PS_5Cl with varying disorder ratios
Fig. 11: Finite element simulations with GBs
Fig. 12: Sensitivity of diffusivity to GB width
Fig. 13: Non-Arrhenius in ordered argyrodites
Fig. 14: Arrhenius-like in disordered argyrodites
Fig. 15: Diffusivities of argyrodites with 1 µm grains
Fig. 16: Li-ion diffusivities in single-type GBs
Fig. 17: Columnar vs. polycrystalline models
Fig. 18: GB effects in argyrodite electrolytes
Fig. 19: Li-ion diffusion barrier vs. anion radius
Fig. 20: Predicted diffusivities vs. grain size
Fig. 21: Map of atomistic- to continuum-scales
Fig. 22: Diffusivities of porous argyrodites with GBs

* yongliang.ou@imw.uni-stuttgart.de
† scholz@mib.uni-stuttgart.de
‡ fritzen@mib.uni-stuttgart.de
§ blazej.grabowski@imw.uni-stuttgart.de

Suppl. Note 1. Parameterization of moment tensor potentials

Supplementary Fig. 1 shows an overview of the parameterization scheme for moment tensor potentials, with extended information shown in Suppl. Fig. 2. The concept of quality-level-based active learning was proposed and tested in a previous study [1] for the same material system, i.e., argyrodites. Initially, ab initio molecular dynamics simulations are performed on the ideal bulk structure. Considering the high computational cost of ab initio molecular dynamics simulations, the simulation temperature is set relatively high (up to the melting point), and the simulation time is set relatively short (a few picoseconds). The high-temperature molecular dynamics simulations enable a quick exploration of the phase space for a fixed chemical configuration by thermal vibrations, and the resulting trajectories provide the basis for the moment tensor potential training. The initial optimization of the moment tensor potential parameters is essential in calculating the extrapolation grade based on the D-optimality criterion [2]. Since the ab initio molecular dynamics trajectories contain structurally similar configurations, only a small number of configurations from the ab initio molecular dynamics are chosen for the initial moment tensor potential training, and a pretraining step is introduced to include more configurations evaluated as extrapolation, i.e., with an extrapolation grade larger than one. Unlike standard active learning schemes, where a high level of moment tensor potential is directly used for active learning of complex structures, a low level of moment tensor potential is used for standard active learning, and the target material properties (for example, formation energy or diffusion coefficient) are evaluated at the end of active learning with the obtained low-level moment tensor potentials. If the accuracy of the target material properties is insufficient, a higher level of untrained moment tensor potential is used, and the pretraining, the standard active learning, and the evaluation processes are repeated. During the step-by-step training of moment tensor potentials with an increasing level, the configurations selected by active learning and labeled by density-functional theory are accumulated in the training set. The final moment tensor potential is obtained when the accuracy of the target material property is satisfied.

In our previous paper [1] focusing on grain boundaries in Li_6PS_5Cl, only three small grain boundaries were considered, and the full targeted grain boundary (GB) structure was used for active learning. This process is classified as global-AL. When the target structure is large, e.g., a large GB structure with more than 30 000 atoms, the density-functional theory calculation of the target structure selected by active learning becomes problematic. To overcome this problem, we have incorporated the quality-level-based concept with active learning of the local atomic environments (local-AL). The local-AL process proposed here has five main steps (Suppl. Fig. 3).

(i) Sampling. The moment tensor potential obtained after pretraining is used to run molecular dynamics simulations of the full target structure. For each snapshot resulting from molecular dynamics simulations, the extrapolation grade based on the local atomic environment is evaluated for each atom, and the maximum local grade of all atoms is focused on. Hyperparameters related to the threshold local grade, i.e., when to select the snapshot or stop the molecular dynamics simulations, need to be set. When the maximum local grade of a snapshot is larger than the set threshold, the snapshot is added to and accumulated in a sampling set.

(ii) Extraction. In this step, the number of atoms in the simulation cell, which is used for labeling (energy and forces calculation with density-functional theory) later on, is significantly reduced. First, the atom that has the maximum local grade is centered in the simulation cell. The local atomic environment of the centered atom is obtained by cutting out the atoms within a cubic box around the centered atom. The cubic box forms a new simulation cell with periodic boundary conditions. Next, atoms closely positioned due to the periodic boundary conditions are removed until the stoichiometry of atoms in the simulation cell is ensured.

(iii) Relaxation. This step aims to avoid unfavorable interactions of atoms at the boundary of the simulation box due to the periodic boundary conditions. To protect the local atomic environment that has been sampled, a protected region in the form of a cubic box is defined around the central atom, and the atoms within the protected region are fixed during the relaxation. The rest of the atoms in the simulation cell are relaxed to minimize the atomic forces. To reduce computational cost, density-functional theory calculations are performed with looser numerical convergence parameters. In this step, only the final relaxed structure enters the next step. Energies and forces obtained during structural relaxation do not enter the training set, maintaining the consistency of the data accuracy of the training set.

(iv) Labeling. The energies and forces of the relaxed structure are calculated by density-functional theory with tight convergence parameters. Because only single-point density-functional theory calculations are performed, the computational cost is kept low. The resulting energies and forces of all atoms in the simulation cell with the corresponding relaxed structures are added to and accumulated in the training set.

(v) Updating. The moment tensor potential, either initialized or obtained from the previous round of active learning, is retrained using the updated training set. The resulting moment tensor potential is then used for the next round of local-AL, starting from Sampling.

With this approach, active learning can be efficiently applied to large and structurally complex systems. Since ab initio molecular dynamics simulations are required only for the bulk unit cell, the overall computational cost is significantly reduced. A schematic overview of the multiscale modeling framework incorporating local active learning is presented in Suppl. Fig. 4.

Suppl. Note 2. Validation of moment tensor potentials

The accuracy and efficiency of the quality-level-based local active learning scheme are demonstrated in Suppl. Figs. 5 and 6 for a $\Sigma3$ GB. Supplementary Fig. 7 shows the training energy and force errors obtained independently for various GB types ranging from $\Sigma3$ up to $\Sigma99$. Although large grain boundaries such as $\Sigma9$ and $\Sigma11$ are structurally more complex than $\Sigma3$ grain boundaries, only small increases are shown for training errors in energy (within $2\,\mathrm{meV\,atom^{-1}}$) and force (within $0.2\,\mathrm{eV\,Å^{-1}}$). These results indicate that GB types exert only a limited effect on the training structures obtained via local active learning.

We also evaluated the accuracy of the moment tensor potentials for a $\Sigma9$ GB and provide a comparison of the results to a $\Sigma3$ GB in Suppl. Fig. 8. Moment tensor potentials reproduce the density-functional-theory-derived grain-boundary formation energies for both GB types. Additional density-functional-theory results also confirm that complex grain boundaries such as $\Sigma9$ generally have higher formation energy than a $\Sigma3$ GB. Next, we performed ab initio molecular dynamics simulations for the $\Sigma3$ and $\Sigma9$ grain-boundary structures at 600 K, to generate configurations for validating forces predicted by the moment tensor potentials. Results show that errors for both grain boundaries are less than $0.2\,\mathrm{eV\,Å^{-1}}$, with the errors for $\Sigma9$ only about $0.03\,\mathrm{eV\,Å^{-1}}$ higher, confirming the high accuracy of the developed moment tensor potentials.

Supplementary Fig. 9 shows a comparison of results from moment tensor potentials and previous ab initio studies using the representative system Li_6PS_5Cl with 50% anion disorder. We performed independent molecular dynamics simulations for six simulation models with random arrangement of Cl/S atoms. Different arrangements of Cl/S atoms were also considered for the RSC Adv. study [3]. Diffusion barriers predicted from our work show much smaller variations due to different Cl/S arrangements (Suppl. Fig. 9**a**), likely due to the three orders of magnitude more atoms in the simulation cell and an order of magnitude longer simulation time compared to prior studies (Suppl. Fig. 9**c**). Nevertheless, the predicted diffusion barrier of 213 meV from our study is within the range reported in previous ab initio studies (202 to 282 meV) [3–5]. In all studies, diffusion coefficients at 300 K were derived by extrapolating high-temperature diffusivities using Arrhenius relations (Suppl. Fig. 9**b**). Such extrapolation is sensitive to statistical errors of the Arrhenius fitting and availability of low-temperature data points. The predicted diffusion coefficients are higher than previously reported values but remain within one order of magnitude. Discrepancies may arise from the limited length and time scales accessible in earlier ab initio studies. We further validated the MTP-MD–predicted diffusivities by comparison with experimental values for anion-disordered Li_6PS_5Cl (Suppl. Fig. 10). Our results show good agreement with quasielastic neutron scattering measurements.

The transferability of moment tensor potentials developed via local active learning was evaluated across the anion ordering chemical space in argyrodites (Suppl. Table 1). Transferability is relatively good for Li_6PS_5Cl, whereas it is limited for Li_6PS_5Br and Li_6PS_5I.

Suppl. Note 3. Consistency of continuum simulation models

A. Methodology

We employed two representations for GBs in polycrystalline solid electrolytes: (i) a volume-resolved GB model, in which the GB domain is explicitly meshed (Suppl. Fig. 11**a**), and (ii) a thickness-collapsed model, in which two-dimensional submanifolds represent GBs (Suppl. Fig. 11**b**). The volume-resolved approach is feasible only when the GB width exceeds the numerical mesh size and when sufficient spatial resolution is available to capture the behavior within the GB domain. Hence, this model is limited to nanograin setups. In contrast, the collapsed GB model assumes a thin GB—typical for micrograins—so there is a clear separation of length scales for grain size and GB width. Due to the conceptually different assumptions concerning the GB representation, a straightforward quantitative comparison of their results is non-trivial. We therefore systematically studied the respective applicability regimes required to guarantee physically sound results. In particular, a transition grain size was determined at which one should switch from the volume-resolved to the thickness-collapsed model.

To isolate the intrinsic behavior of the two continuum models and eliminate any geometrical artifacts, we employed a highly idealized microstructure: a two-grain Voronoi tessellation. This configuration is perfectly isotropic, features symmetric GB junctions, and keeps the computational cost low. The collapsed approach allows for a parametric variation of the GB width on a single mesh. In contrast, changing the GB width in a resolved model requires rebuilding the three-dimensional voxel image each time to properly identify GB voxels. Therefore, we generated resolved polycrystalline models for a fixed spatial resolution where the GB width is a multiple of the voxel size. Because the considered Voronoi tessellation was fixed, increasing the GB width reduces the effective grain size. Designing a corresponding collapsed model that matches the GB width and GB volume fraction of the resolved model is then straightforward. This was done by rescaling the GB width parameter together with the simulation cell length. Simulations were then carried out for both GB representations independently for a range of bulk and GB diffusivities, D^{bulk} and $D^{\mathrm{GB}} := D^{\mathrm{GB}}_{\parallel} = D^{\mathrm{GB}}_{\perp}$. By repeating the procedure at several mesh resolutions, we could (i) compare the results of both models across different grain size regimes depending on the inputs $D^{\mathrm{GB}}/D^{\mathrm{bulk}}$, and (ii) assess how the spatial resolution influences the accuracy of the resolved approach. From these systematic studies, we (i) identified

the transition grain size at which the modeling strategy should be switched (Suppl. Note 3 B), and (ii) formulated a practical guideline for selecting a mesh resolution that ensures convergence and accurate results for the resolved approach (Suppl. Note 3 C).

B. Transition grain size and agreement of the models

The true diffusion behavior of a polycrystalline material cannot be measured in detail, so the following considerations are based on two assumptions: (i) the resolved approach yields accurate results in the nanograin regime, and (ii) the collapsed model reliably reproduces diffusion in the micrograin regime. When both models are applied across the entire range of grain sizes, we therefore expect a transition point in between the nanograin and micrograin limits where the agreement is significantly improved compared to the two extremes. This is supported by the relatively small deviation in macroscopic diffusivity for medium-sized grains (Suppl. Fig. 11**c**). Consequently, we define the transition grain size as the grain size at which the two models are most consistent, regardless of the GB-to-bulk diffusion contrast $D^{\mathrm{GB}}/D^{\mathrm{bulk}}$, i.e., we minimize the sum of squared relative deviations. The transition grain size (indicated by the dashed lines in Suppl. Fig. 11**c**) depends strongly on the spatial resolution of the resolved model, which will be discussed in Suppl. Note 3 C. Upon mesh refinement, the transition size converges to about 100 nm. This value was therefore adopted as a reference for all continuum-model results presented in this study. For the computations that cover the entire grain size range (e.g., Suppl. Fig. 20), both the resolved and the collapsed representations were evaluated slightly beyond the identified transition grain size to demonstrate the consistency of the two approaches.

The obtained transition grain size of 100 nm is specific to the considered GB width of 2.5 nm that was assumed for the given geometry. Nevertheless, the same procedure can be applied to any GB width or microstructure. Converting the transition grain size to a GB volume fraction yields approximately 6.3%, which serves as a GB width-independent indicator of the regime change.

Even in the vicinity of the transition grain size, the two approaches do not achieve perfect agreement (Suppl. Fig. 11**c**). This residual mismatch stems from inherent differences between the two GB representations: (i) the explicit representation of GB junction domains in the volume-resolved approach versus their implicit treatment in the collapsed approach, (ii) the volume corrections required for the collapsed approach, and (iii) the underlying microstructures considered for the two approaches are not identical but only match key characteristics. Nevertheless, the deviation is modest—typically below 5%—at the transition grain size.

Large deviations occur primarily for blocking GBs, i.e., when the GB diffusivity is lower than the bulk diffusivity ($D^{\mathrm{GB}} < D^{\mathrm{bulk}}$). In this regime, the less-conductive GBs induce pronounced concentration jumps across the GB. These jumps and their effects are especially difficult to capture if not resolved well, as in the following two cases: (i) in volume-resolved simulations of micrograins, a GB may be represented by only a few voxel layers, leading to an under-resolved GB domain and noticeable staircasing effects; and (ii) in the collapsed approach for nanograins, the zero-width assumption neglects a substantial GB volume. The impact of resolution in case (i) on concentration and flux fields is shown in Suppl. Fig. 11**d** for the smallest GB width relative to the grain size. Notably, the volume fraction varies substantially with resolution, even when the GB width remains fixed. This leads to differences in the flux magnitude in the presented results obtained with the volume-resolved approach.

When GB diffusion dominates in the polycrystalline model ($D^{\mathrm{GB}} > D^{\mathrm{bulk}}$), results from the volume-resolved and thickness-collapsed approaches converge for micrograins. In this diffusion regime, GBs do not cause concentration discontinuities but serve as additional pathways. Because the grains are large, the contribution of these pathways to the overall transport is modest, so the agreement between the two models persists even when the GB width is resolved by only a few voxel layers. Therefore, the volume-resolved approach can be employed reliably for grain sizes beyond the estimated transition grain size (100 nm).

C. Resolution requirements for volume-resolved grain boundaries

When a resolved microstructure is generated for a prescribed GB width for different resolutions, the resulting GB volume fraction varies slightly because the voxel-based discretization only approximates the GB domain. This prevents a direct comparison of the macroscopic diffusivities across different resolutions, since the GB volume fraction itself influences the resulting macroscopic diffusivities. Therefore, the convergence behavior of the volume-resolved approach cannot be evaluated unambiguously. Supplementary Fig. 11**c** shows that the mesh resolution has a noticeable impact on the accuracy of the results, considering the corresponding results from the collapsed approach as a resolution-independent reference.

In the polycrystalline model, the relative GB width decreases with increasing grain size. Consequently, for a given resolution, the number of voxel layers representing a GB varies with grain size. Supplementary Fig. 11**c** shows that only a few layers were used to resolve a GB for micrograins, and more layers were used for nanograins. The resolution effect is most pronounced for blocking GBs ($D^{\mathrm{GB}} < D^{\mathrm{bulk}}$) combined with large grains, i.e., when the resolved model under-resolves the GB. The convergence of the relative error curves with increasing resolution implies a practical rule: The GB width should be at least ten times the voxel length. For a given geometry, the GB width in the unit

cube is first adjusted to satisfy the prescribed ratio between GB width and grain size. The resolution is then selected such that the voxel size is less than one-tenth of the GB width. In contrast, as discussed in Suppl. Note 3 B, results with an enhancing GB setup ($D^{\mathrm{GB}} > D^{\mathrm{bulk}}$) exhibit almost no sensitivity to mesh resolution. In those cases, a coarser discretization is sufficient: A GB width of more than five times the voxel length yields mesh-independent results. In the present study, all volume-resolved simulations were conducted on a $512 \times 512 \times 512$ voxel grid, which exceeds the resolution required to resolve GBs in polycrystalline models with nanograins (grain size less than 100 nm) while avoiding prohibitive computational cost.

Suppl. Note 4. Sensitivity to grain boundary width

Sensitivity to the GB width can be straightforwardly quantified within our multiscale modeling framework. At the atomistic scale, GB diffusivities derived from polycrystalline models depend on the assumed GB width. For a two-grain polycrystalline model with cubic geometry, the chosen GB widths and the corresponding calculated GB volume fractions are summarized in Suppl. Table 2. Supplementary Fig. 12 shows the predictions obtained using different GB widths across atomistic and continuum scales. Reducing the GB width from 2.5 nm to 1.5 nm yields similar Li-ion diffusion barriers, consistent with experimental measurements. In contrast, a further reduction to 1 nm leads to an overestimation of the diffusion barriers relative to experiment. For polycrystals with small grains, diffusivities obtained from all-atom MD simulations agree well with those predicted by our multiscale framework using a GB width of 2.5 nm. Smaller widths of 1 nm and 1.5 nm introduce inconsistencies between atomistic and continuum-scale simulations.

Suppl. Note 5. Non-Arrhenius transport behavior

Non-Arrhenius behavior is observed for anion-ordered Li_6PS_5I and Li_6PS_5Br, as shown in Suppl. Fig. 13. At higher temperatures, the diffusivity ratios between GB and bulk are relatively small, and the total diffusivity is dominated by the bulk diffusivity due to the large volume fraction of bulk in the polycrystal model. At lower temperatures, the diffusivity ratios between GB and bulk become larger, and the effect of GBs becomes more critical. Therefore, a grain-boundary-dominated regime is found at low temperatures. Such a GB-induced non-Arrhenius behavior was reported in a previous study [6].

On the other hand, the non-Arrhenius behavior cannot be clearly observed for anion-disordered argyrodites in the investigated temperature range, as demonstrated for Li_6PS_5Cl with 50% anion disorder in Suppl. Fig. 14. The bulk and GB diffusivities are similar, even in the low-temperature range, so the polycrystal diffusion behavior closely follows the bulk Arrhenius fit, without showing non-Arrhenius behavior in the investigated temperature range.

Supplementary Fig. 15 compares predicted temperature-dependent diffusivities of different argyrodite polycrystals. Note that the non-Arrhenius behavior, including the transition temperature, is also dependent on the grain size of the polycrystal model. Our results are consistent with experimental observations, which report non-Arrhenius diffusion behavior in Li_6PS_5I but not in Li_6PS_5Br [7]. In experiments, Li_6PS_5I is predominantly anion-ordered, whereas Li_6PS_5Br is generally anion-disordered. Further application of our multiscale modeling framework enables a priori screening of material systems that show anomalous diffusion behavior.

Suppl. Note 6. Additional results and discussion

An overview of investigations on GBs in solid electrolytes is presented in Suppl. Table 3. The present study extends previous investigations to a systematic class of sulfides and a broader set of GBs using accurate machine-learning potentials. Comparisons of Li-ion diffusivities for various single-type GBs and between columnar and polycrystalline grains are shown in Suppl. Figs. 16 and 17, respectively. GB effects across the chemical space of argyrodites, including anion species and ordering, are shown in Suppl. Figs. 18 and 19. The predicted Li-ion diffusivities from this study are summarized in Suppl. Table 4, and the derived hopping frequencies are consistent with experimental values [8]. Using the multiscale modeling framework, grain-size-dependent diffusivities of polycrystalline argyrodites (Suppl. Fig. 20) and a precomputed map linking atomic-scale mechanisms to continuum-scale behavior in general polycrystalline solid electrolytes (Suppl. Fig. 21) are obtained. Moreover, the impact of porosity on macroscopic conductivity can be incorporated on top of these results, as demonstrated for anion-disordered Li_6PS_5Cl (Suppl. Fig. 22).

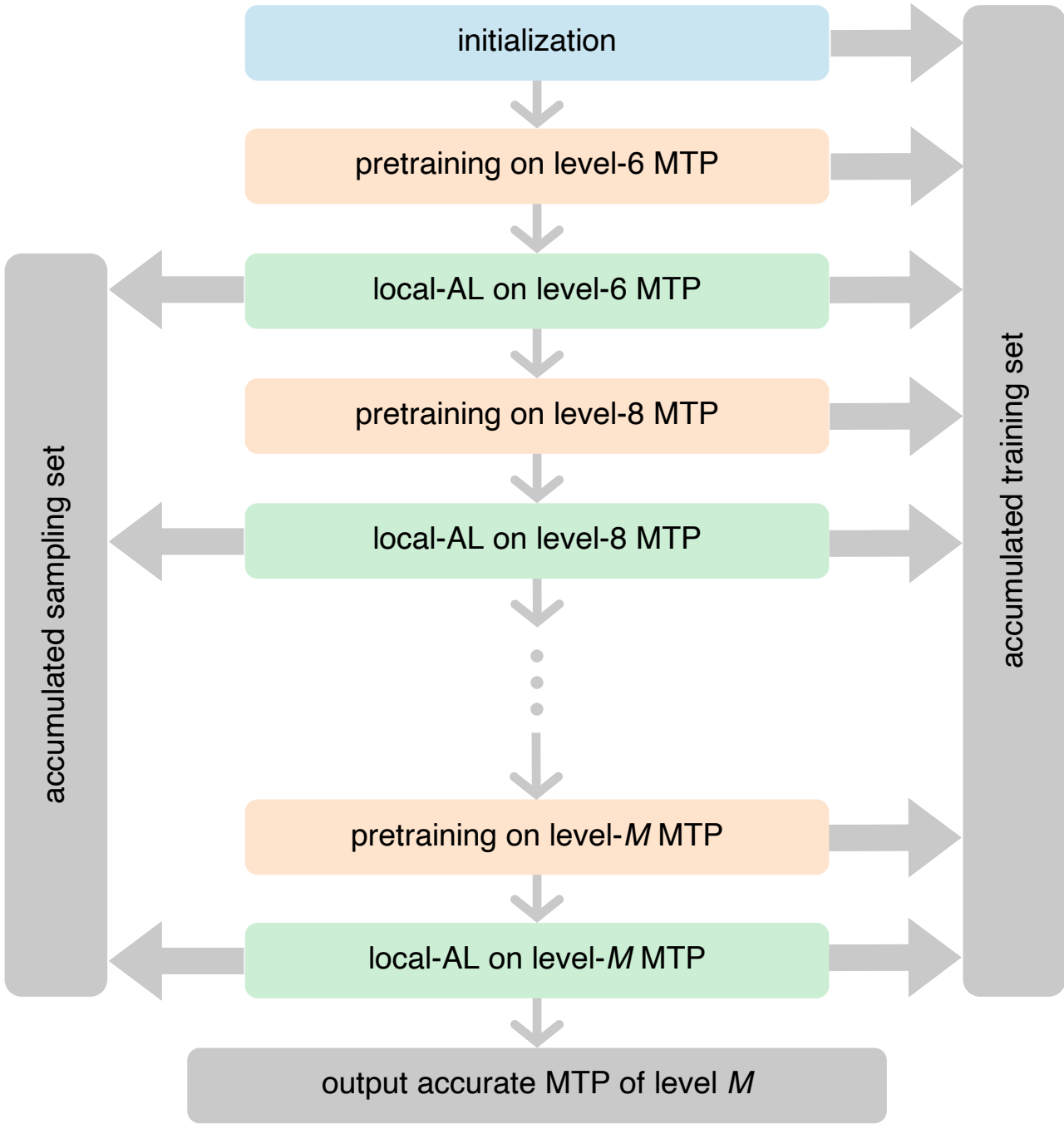

Suppl. Fig. 1. **Overview of the parameterization scheme for moment tensor potentials (MTP).** The quality-level-based concept is emphasized with the changing MTP level.

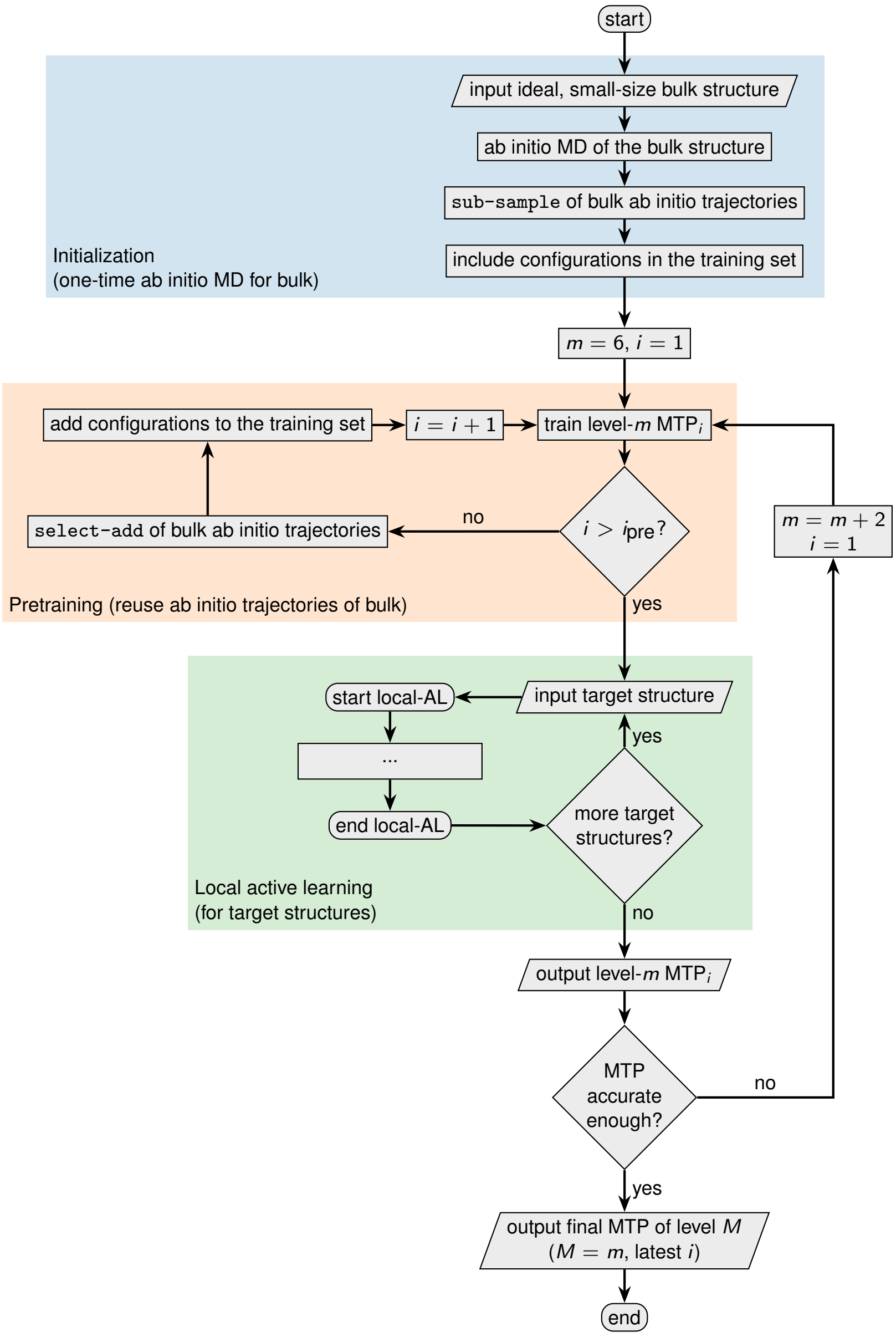

Suppl. Fig. 2. **Complete workflow of the parameterization scheme for moment tensor potentials (MTPs).** MD stands for molecular dynamics simulations, while AL stands for active learning. Details of the local active learning steps, indicated by "$\cdots$" in the present figure, are provided in Suppl. Fig. 3.

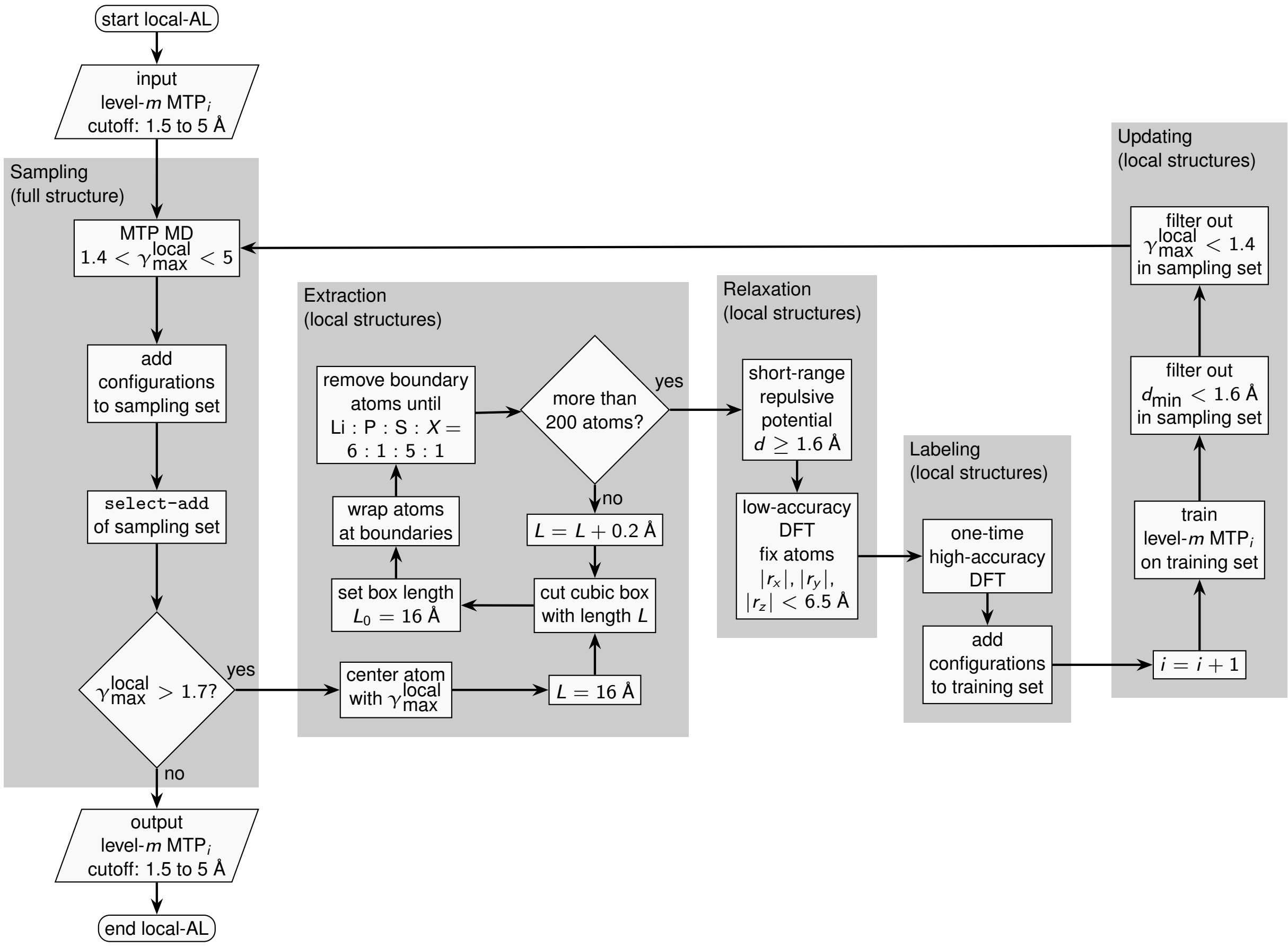

Suppl. Fig. 3. **Details of active learning (AL) for local structures.** Parameters used for Li_6PS_5X with $X \in \{\mathrm{Cl}, \mathrm{Br}, \mathrm{I}\}$ are shown. MTP-MD and DFT refer to molecular dynamics simulations with moment tensor potentials and density-functional theory, respectively. The extrapolation grade and atomic distance are denoted by γ and d, respectively.

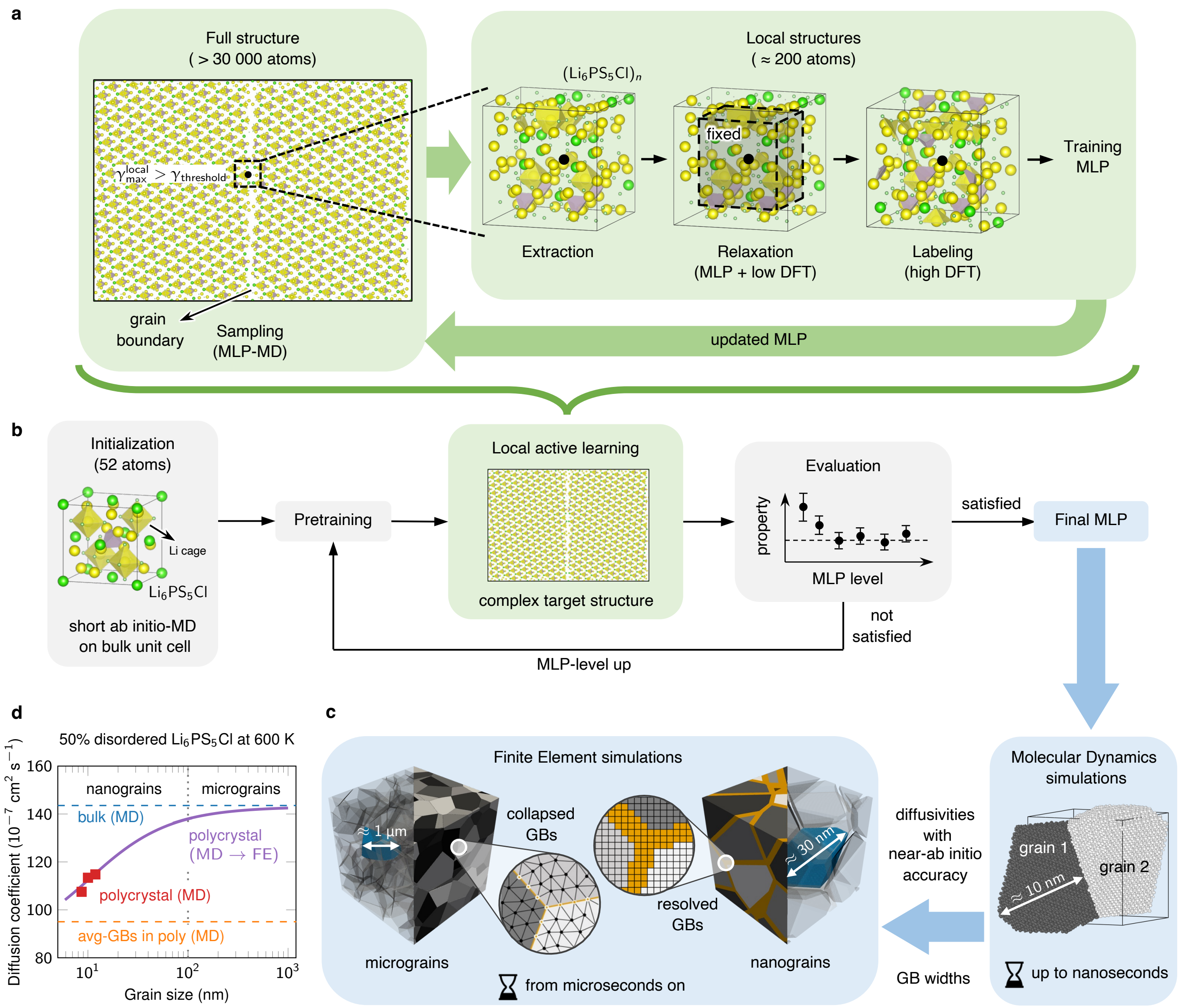

Suppl. Fig. 4. **Schematic illustration of the multiscale modeling framework for resolving Li-ion transport in polycrystalline solid electrolytes.** **a**, Closed-loop local active learning. Local structures are extracted from the full structure for efficient labeling with density-functional theory (DFT). Sampling is based on the local grade (γ^{local}) from the machine-learning potentials (MLPs). The extracted local structure retains its stoichiometry. Active learning finishes when no more configurations are sampled from MD simulations. **b**, Quality-level-based parameterization. Accurate and efficient MLP fitting is performed for complex structures, illustrated here with ordered Li_6PS_5Cl as an example. The Σ99[110] grain boundary (GB) embedded in a bicrystalline model is an example of the target structure. After initialization, a low level of MLP is used for pretraining and active learning. Then, the quality of the obtained MLP is evaluated based on the material properties of interest. If the MLP quality is not satisfied, a higher level of MLP is used for the next stage of active learning. **c**, Connecting atomic-scale to continuum-scale simulations. Diffusivities with near-ab initio accuracy from MD simulations are inputs for FE simulations. Different approaches are employed on the continuum scale to simulate GBs in polycrystals with nanograins and micrograins. **d**, Example application of the multiscale modeling framework (MD → FE) for predicting diffusivities of polycrystalline anion-disordered Li_6PS_5Cl at 600 K. Results from MD simulations of polycrystalline models are compared with FE simulations to demonstrate their consistency.

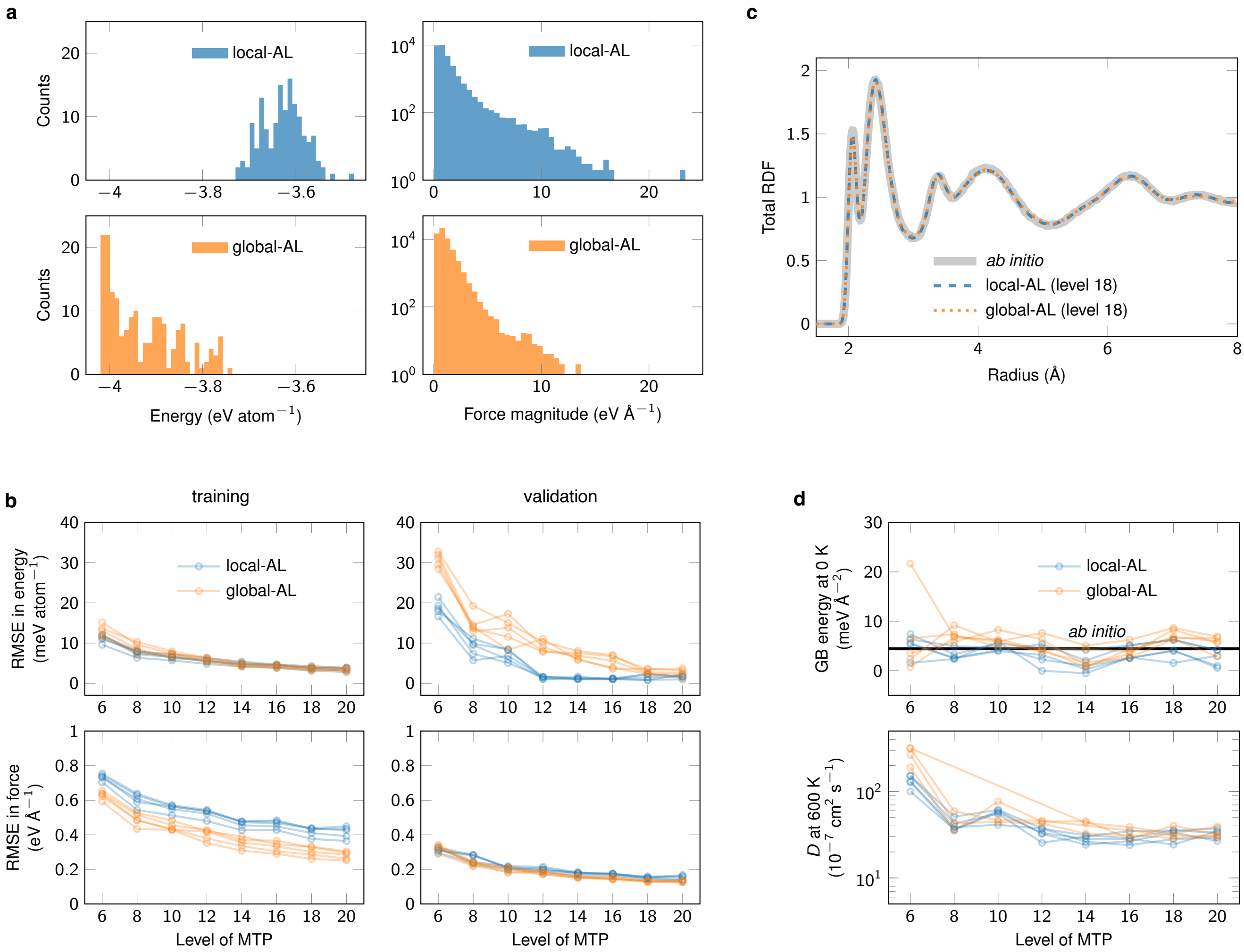

Suppl. Fig. 5. **Accuracy of the quality-level-based local active learning scheme.** **a**, Energy and force distributions of configurations sampled using local- or global-active learning (AL) schemes up to level 20. The term global-AL refers to cases where uncertainty is quantified over the entire simulation model, whereas local-AL refers to cases where uncertainty is quantified individually for each atom in the model. The configurations used to train moment tensor potentials (MTPs) in the local-AL scheme are extracted local structures and thus exhibit higher potential energies. **b**, Root mean square errors (RMSEs) of the trained MTPs from level 6 to 20 for the training and the validation sets. For MTPs resulting from the local-AL scheme, smaller RMSEs are observed in potential energy for the validation set, while larger RMSEs are seen in atomic force for both the training and validation sets. The validation set is obtained by MD simulations of grain boundary (GB) structures at 600 K. **c**, Time-averaged radial distribution functions (RDFs) of the configurations obtained by running ab initio-molecular dynamics simulations and molecular dynamics simulations with level-18 MTPs for the $\Sigma 3$ GB in anion-ordered Li_6PS_5Cl. The MTPs were trained using the quality-level-based scheme with local- or global-AL. Good agreement is observed between ab initio- and MTP-derived RDFs. **d**, Comparison of the predicted GB formation energy and diffusivity for the $\Sigma 3$ GB, using MTPs trained with local- or global-AL. The GB energy from ab initio density-functional theory calculations is compared. MTPs trained to higher levels (e.g., level 18) yield consistent results for both AL schemes. To obtain statistics, five MTPs were independently trained with the same AL scheme and subsequently employed for RMSE and property analysis.

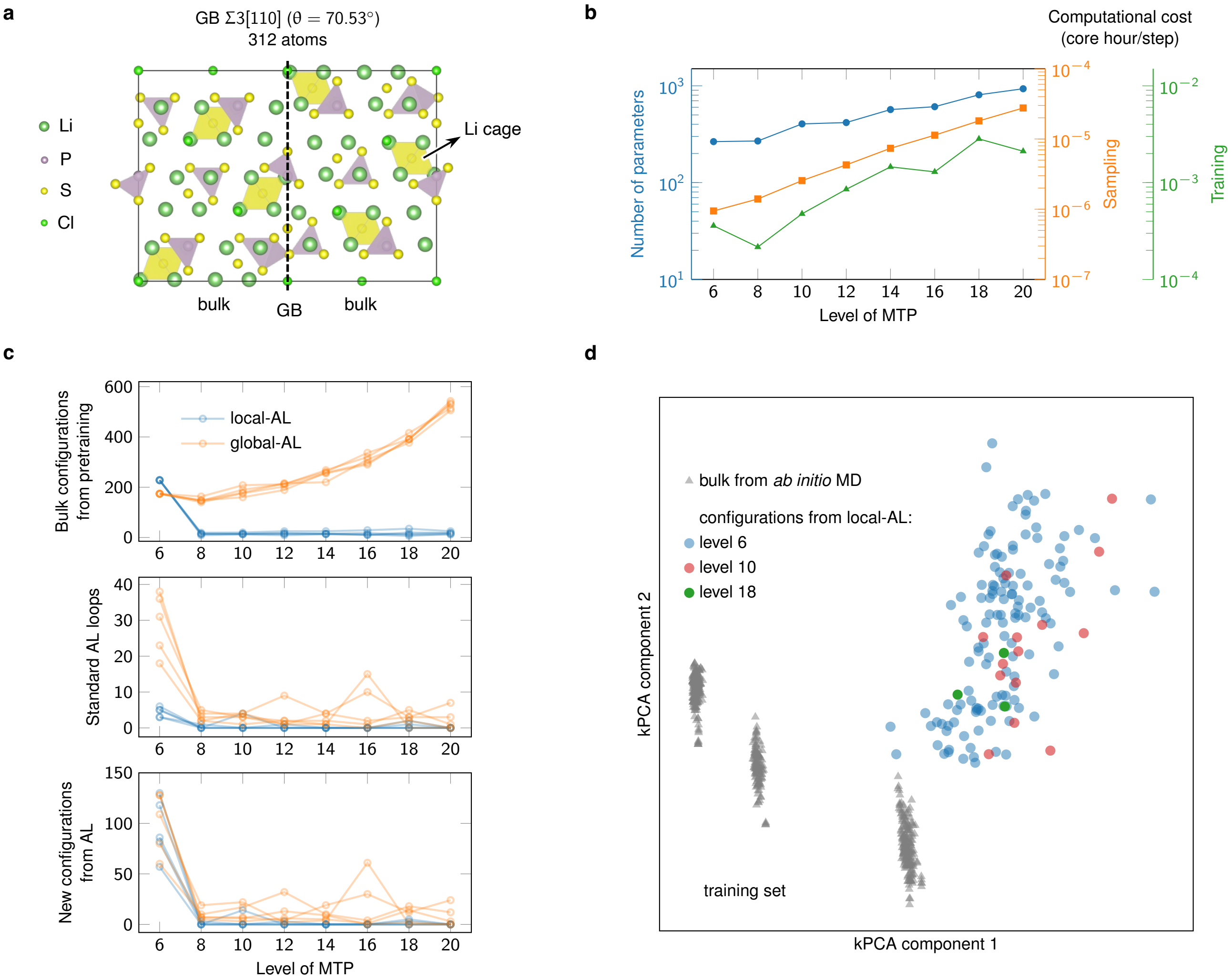

Suppl. Fig. 6. **Efficiency of the quality-level-based local active learning scheme.** **a**, Atomistic structure of a bicrystalline model consisting of 312 atoms, incorporating single-type Σ3 grain boundaries (GBs). The anion-ordered bulk structures of Li_6PS_5Cl were rotated by $\theta = 70.53°$ in the [110] direction to construct the GB. The model is set as the target structure for comparison of local- and global-active learning (AL). **b**, Number of fitting parameters in moment tensor potentials (MTPs) from level 6 to 20, along with the computational costs associated with MTP-based molecular dynamics simulations of the target structure (sampling) and MTP training. All of them increase exponentially with the increasing level of MTP. **c**, Comparison of the performance between local-AL and global-AL schemes for the targeted bicrystalline model. At the end of each level of AL, the number of bulk configurations added to the training set during pretraining, the number of standard-AL loops performed, and the number of new configurations sampled and labeled during AL are presented. Fewer configurations are needed to train the MTPs using the local-AL scheme. Each AL scheme was performed five times for statistics. **d**, Kernel principal component analysis (kPCA) of the training set after local-AL up to level 20. More diverse configurations were sampled at lower levels of AL compared to higher levels. Structural representations were constructed using smooth-overlap-of-atomic-positions (SOAP) [9] descriptors.

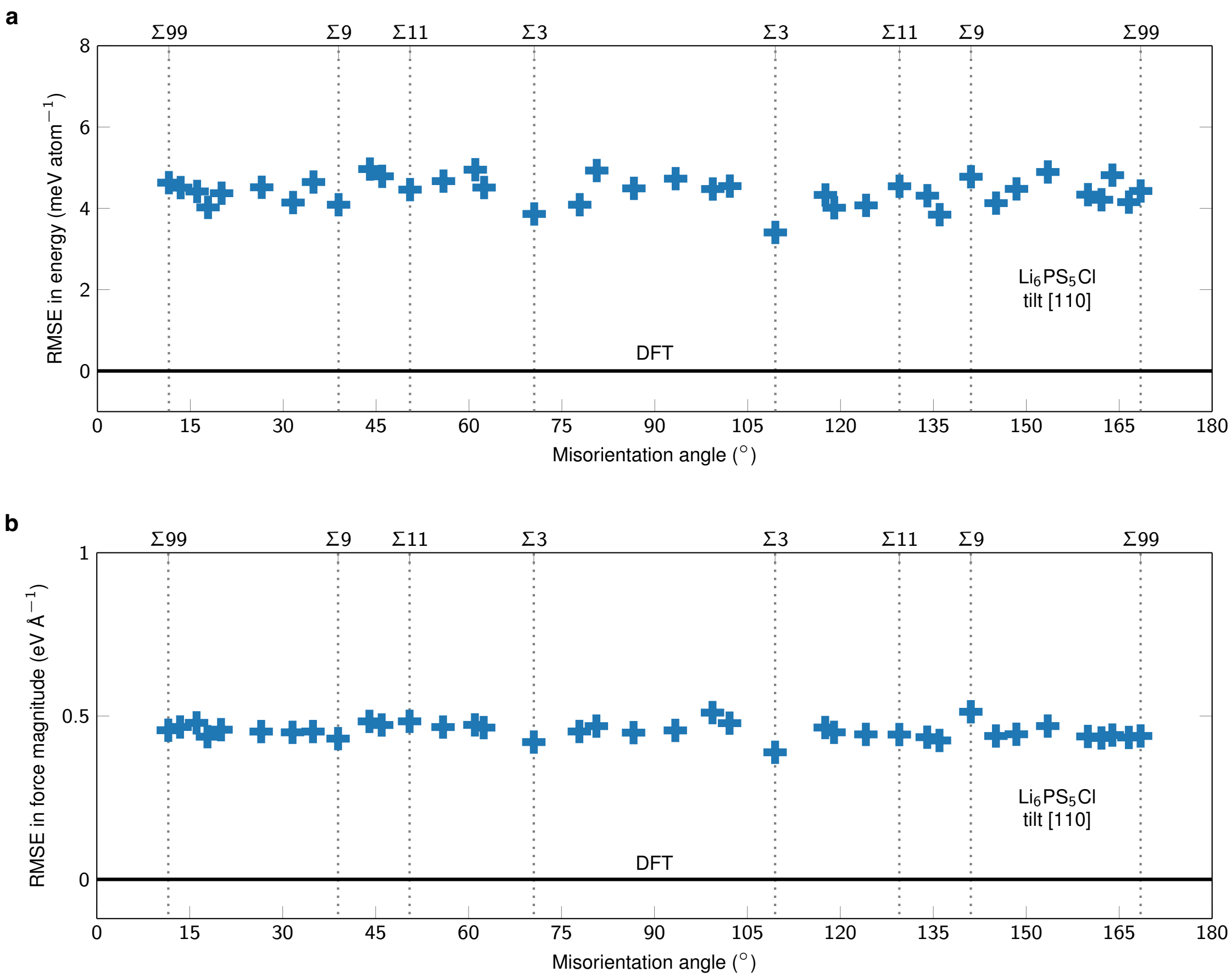

Suppl. Fig. 7. **Energy and force errors for training structures obtained through local active learning.** **a**, Root-mean-square errors (RMSEs) in machine-learning-potential-predicted energies are around 4 meV atom^{-1}. **b**, RMSEs in force are around 0.5 eV Å^{-1}. Errors in energies and forces remain consistent across different grain-boundary types. Results are based on single-type tilt grain boundaries in anion-ordered Li_6PS_5Cl with a [110] rotation axis.

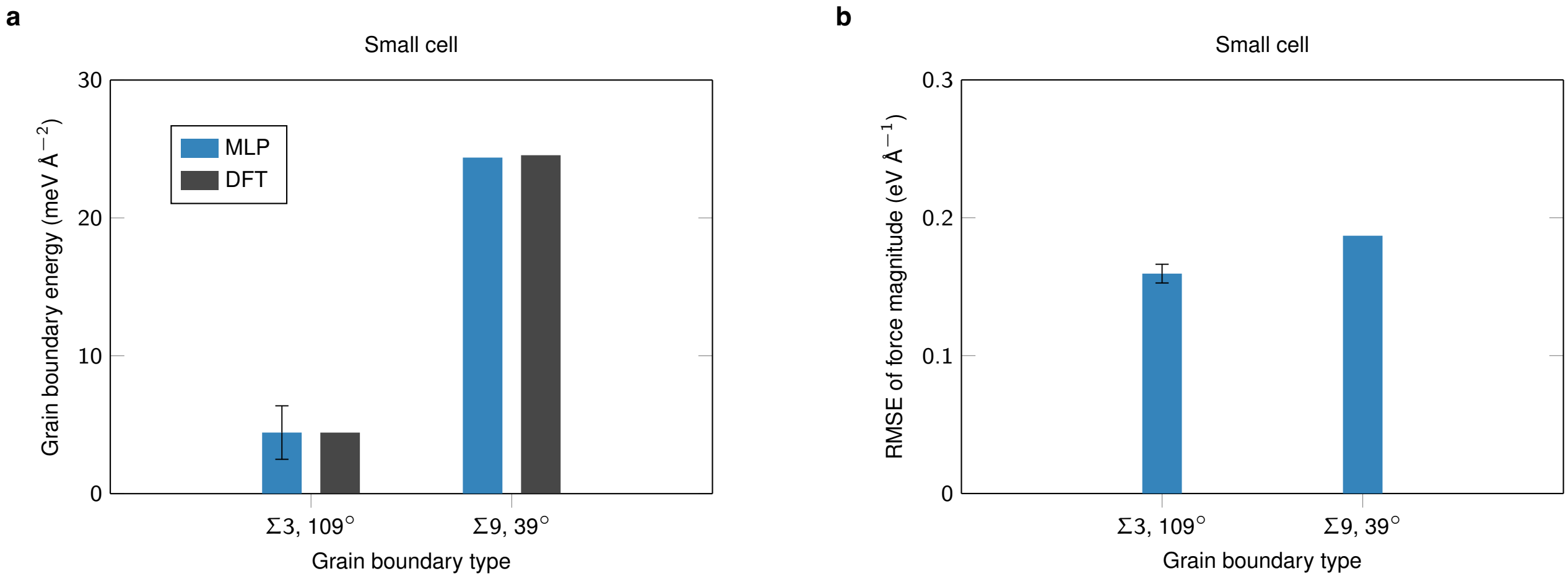

Suppl. Fig. 8. **Validation of machine-learning potentials (MLPs) for different types of grain boundaries.** Results for representative Σ3 and Σ9 grain boundaries in Li_6PS_5Cl, with misorientation angles of about 109° and 39°, respectively, are compared. **a**, Grain-boundary energetics at 0 K. MLPs reproduce grain-boundary formation energies obtained from density-functional theory (DFT). Σ9 grain boundaries exhibit formation energies about 20 meV Å^{-2} higher than Σ3. Error bars represent standard deviation of results from five independently trained potentials. **b**, Force prediction errors at 600 K. Ab initio molecular dynamics simulations of grain-boundary structures at 600 K were used to generate validation configurations. Force errors for both Σ3 and Σ9 grain boundaries remain below 0.2 eV Å^{-1}. Due to the high computational cost of DFT, grain-boundary structures were modeled in small simulation cells. Error bars represent the standard deviation across five independently trained MLPs obtained via local active learning.

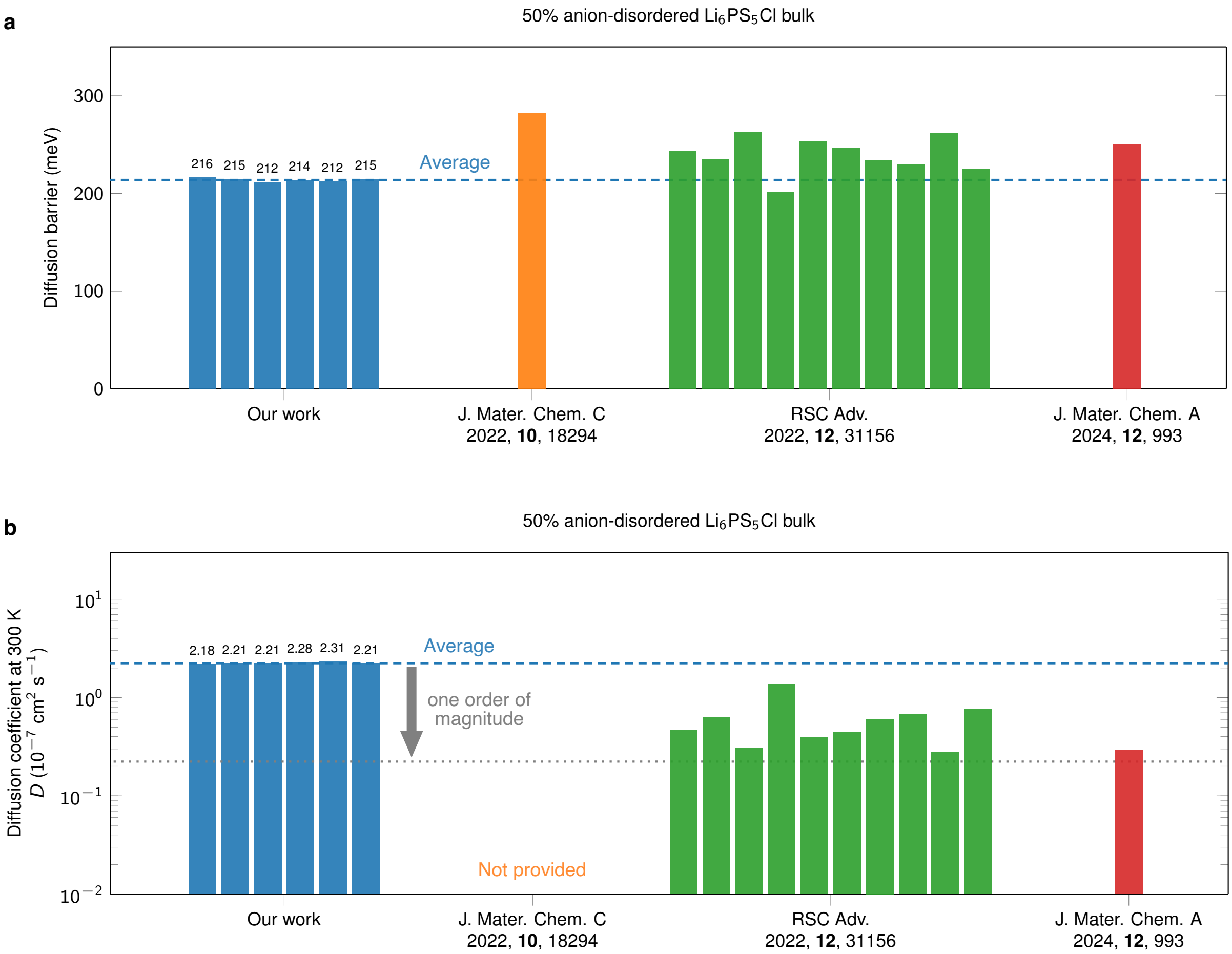

c

	Our work	J. Mater. Chem. C 2022, **10**, 18294	RSC Adv. 2022, **12**, 31156	J. Mater. Chem. A 2024, **12**, 993
Simulation method	ab initio → MLP	ab initio	ab initio	ab initio
Simulation model (atoms)	52 000	52	52	52
Simulation time (ps)	5 000	120	150	300
Lowest simulation temperature (K)	350	500	600	700

Suppl. Fig. 9. **Comparison of MLP–MD-predicted diffusion properties with ab initio results.** In all studies, molecular dynamics simulations are performed and diffusion properties are extracted from the mean-squared displacement of Li ions. Bulk Li_6PS_5Cl with 50% anion disorder is considered. **a**, Comparison of diffusion barriers. **b**, Comparison of diffusion coefficients extrapolated to 300 K. Dashed lines indicate averaged values from our work and facilitate comparison with previous studies (J. Mater. Chem. C [4], RSC Adv. [3], J. Mater. Chem. A [5]). A dotted line indicates a value one order of magnitude lower than our predicted diffusivities for reference. **c**, Comparison of simulation length and time scales, together with the lowest temperatures sampled. Larger simulation models are essential to capture realistic anion disorder, whereas longer simulation times are required to obtain statistically converged diffusivities based on the Einstein relation. Additionally, access to lower temperatures is critical for reliable Arrhenius extrapolation to obtain room-temperature diffusivities.

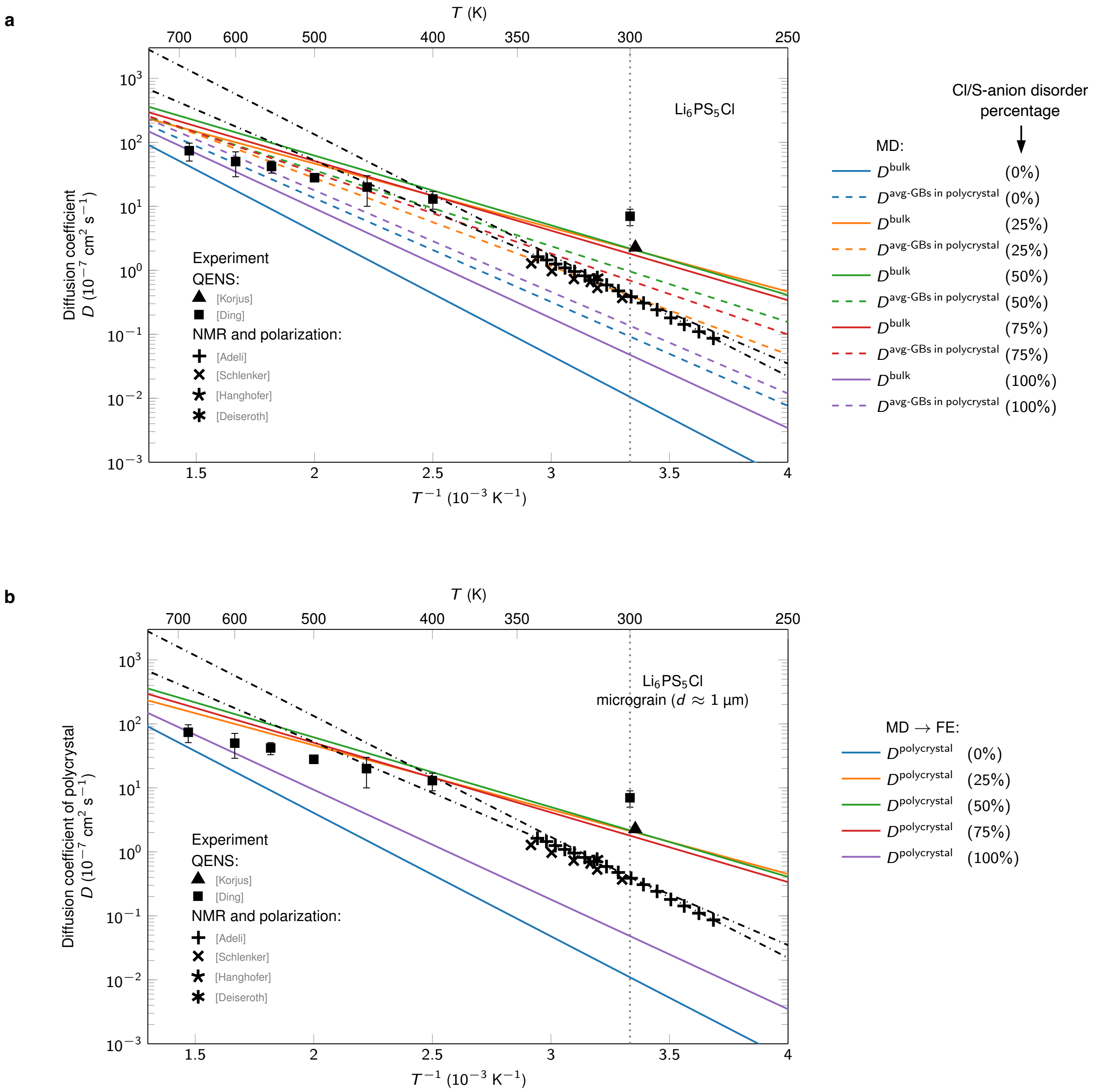

Suppl. Fig. 10. **Grain-boundary (GB) effects in Li_6PS_5Cl at varying anion-disorder ratios.** **a**, Arrhenius fits of predicted diffusion coefficients of bulk and GB structures in Li_6PS_5Cl with varying percentages of Cl/S-anion disorder. The GB values (avg-GBs in polycrystal) were extracted from molecular dynamics simulations of polycrystalline models with random anion disorder. Experimentally measured diffusion coefficients and their uncertainties from Adeli et al. [10], Schlenker et al. [11], Hanghofer et al. [12], Deiseroth et al. [13] (nuclear magnetic resonance and polarization measurements), and Korjus et al. [14], Ding et al. [15] (quasielastic neutron scattering measurements), with samples exhibiting ∼60% anion disorder, are compared. **b**, Predicted diffusion coefficients of polycrystalline Li_6PS_5Cl with an average grain size of 1 µm. Experimental diffusion coefficients fall within the predicted diffusivity domains introduced by chemical and microstructural variations.

Suppl. Table 1. **Transferability of moment tensor potentials developed via local active learning.** Moment tensor potentials trained on polycrystalline models with 50% anion disorder are validated against bulk configurations with different degrees of anion disorder. The validation dataset is generated from molecular dynamics simulations at 600 K. For Li_6PS_5Cl, the potentials show good agreement with reference energies and forces across all considered disorder configurations. In contrast, the potentials for Li_6PS_5Br and Li_6PS_5I exhibit significantly larger errors in both energies and forces.

System	Li_6PS_5Cl	Li_6PS_5Cl	Li_6PS_5Cl	Li_6PS_5Br	Li_6PS_5I
Training anion disorder (%)	50	50	50	50	50
Extrapolating anion disorder (%)	25	75	100	100	100
Energy RMSE (meV atom^{-1})	7.18	8.45	10.06	27.71	15.71
Force RMSE (eV Å^{-1})	0.168	0.181	0.175	0.200	0.213

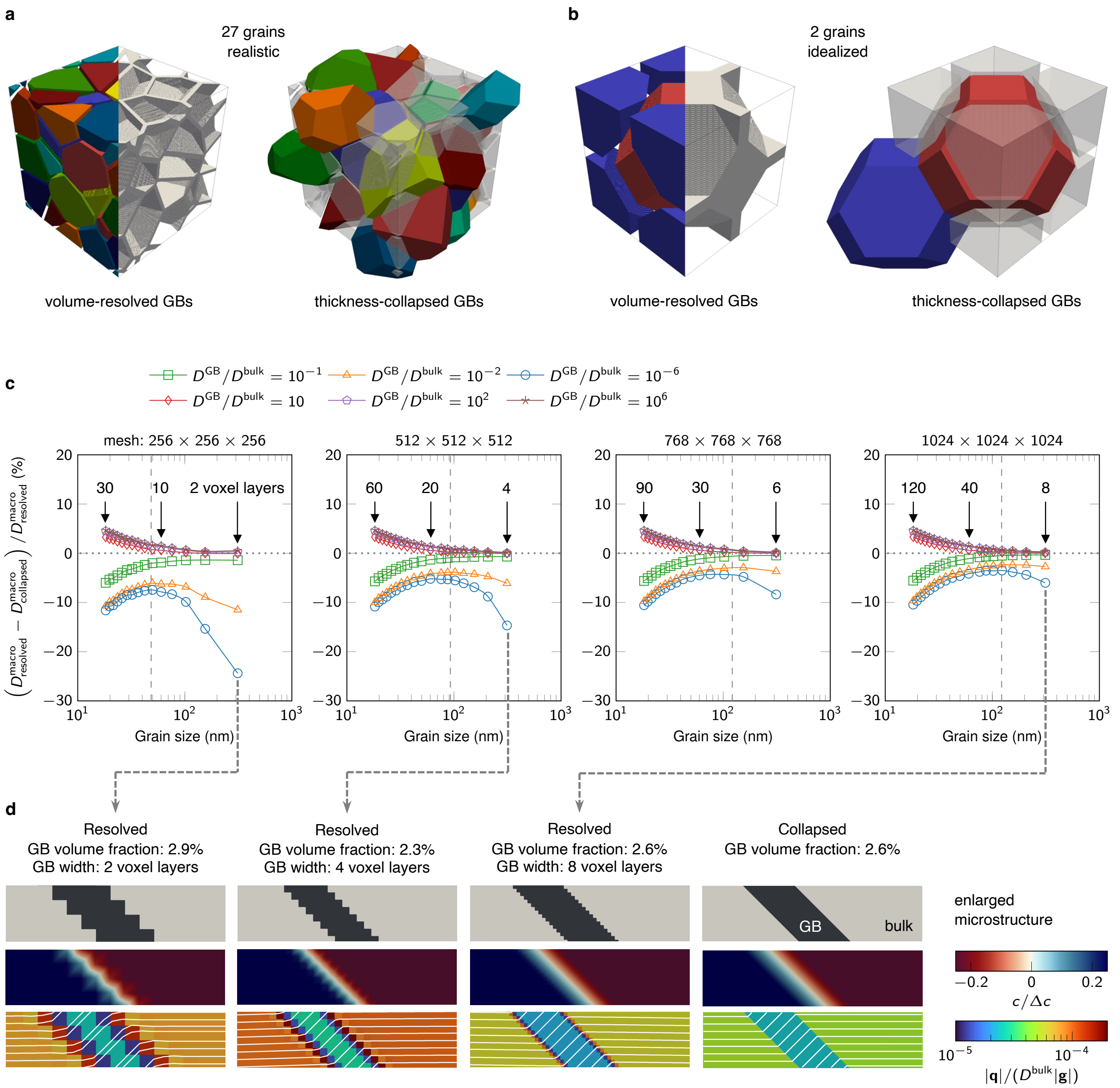

Suppl. Fig. 11. **Finite element models and simulations for predicting macroscopic diffusivity in polycrystalline solid electrolytes.** **a**, Polycrystalline models with 27 grains used as a realistic volume element for finite element (FE) simulations in production runs. Voxel-based image with grain boundaries (GBs) of finite thickness (volume-resolved approach) and the crinkle-cut geometry featuring zero-thickness GBs (thickness-collapsed approach) are shown. **b**, Idealized, isotropic polycrystalline models with two grains used to test the consistency of the two approaches. **c**, Consistency test of the two approaches using the idealized model with the same parametric setup. Deviation in macroscopic diffusivities resulting from the two approaches depends on the mesh resolution of the volume-resolved model, average grain size, and the GB-to-bulk diffusivity ratio. The GB width was varied to enable different grain sizes. Numbers at the arrows indicate the voxel layers used to resolve GBs. The dashed line marks the grain size with the smallest deviation between the two approaches, converging to ~100 nm at higher mesh resolutions. **d**, Enlarged view of the polycrystalline microstructure, the corresponding normalized concentration field c and two-dimensional projection of the normalized flux field $\mathbf{q}$ from FE simulations using the two approaches. The imposed concentration gradient $\mathbf{g}$ and the corresponding concentration jump Δc were used for normalization. The ratio $D^{GB}/D^{bulk} = 10^{-6}$ and grain size ~300 nm are focused on. Increasing mesh resolution reduces differences in concentration and flux fields between the two approaches, consistent with the deviation values.

Suppl. Table 2. **Calculated grain boundary volume fraction for an illustrative two-grain polycrystalline model with cubic geometry.** The simulation cell has a side length of 10 nm. The Voronoi seeds are located at fractional coordinates (0.7120, 0.4287, 0.1273) and (0.3314, 0.5767, 0.3072).

grain boundary width (nm)	grain boundary volume fraction (%)
0.3	10.614639521
0.4	14.063393325
0.5	17.338106036
0.6	20.579787344
0.7	23.666596413
0.8	26.748743653
0.9	29.758719355
1.0	32.612385601
1.1	35.470832884
1.2	38.198748976
1.3	40.934503078
1.4	43.533254415
1.5	46.082925051
1.6	48.511920869
1.7	50.918011367
1.8	53.265763819
1.9	55.476129055
2.0	57.686384767
2.1	59.776703268
2.2	61.869255453
2.3	63.853708655
2.4	65.781814605
2.5	67.630871385
2.6	69.417306781
2.7	71.171044558
2.8	72.814197838
2.9	74.456302822
3.0	76.009458303
3.1	77.504047751
3.2	78.923345357
3.3	80.303093791
3.4	81.643644720
3.5	82.880389690
3.6	84.111364931
3.7	85.245972872
3.8	86.375099421
3.9	87.435894459
4.0	88.439571857
4.1	89.386612922
4.2	90.291553736
4.3	91.164941341
4.4	91.953337938
4.5	92.732436955
4.6	93.431632966
4.7	94.121170789
4.8	94.758135080
4.9	95.344003290

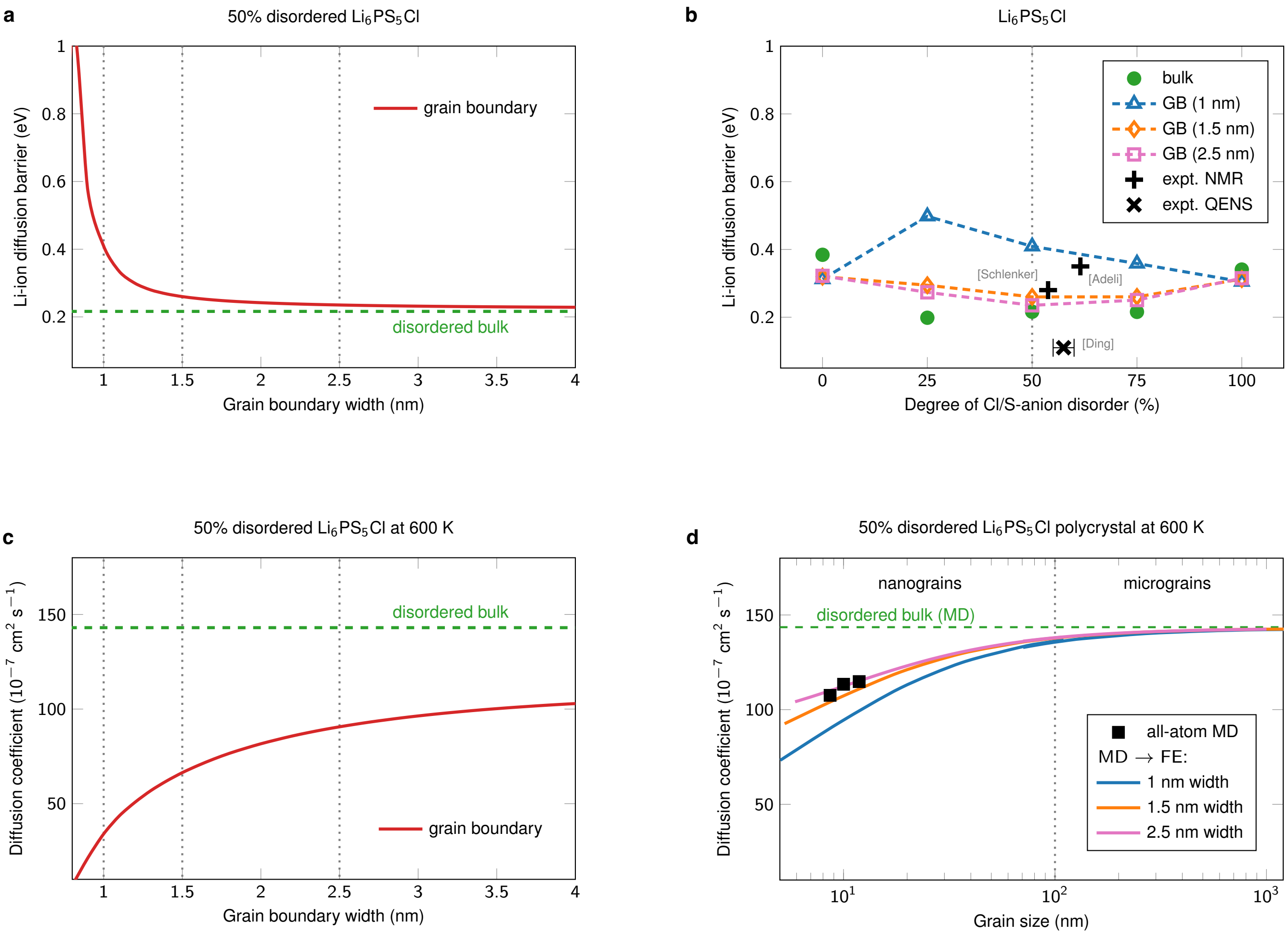

Suppl. Fig. 12. **Sensitivity analysis of grain boundary (GB) width.** A GB width of 2.5 nm was derived from atomistic GB structures and was adopted for the main results in this study. Results obtained using smaller widths of 1.5 and 1 nm are shown in the present figure for comparison. **a**, Predicted Li-ion diffusion barriers in grain boundaries estimated using different GB widths, exemplified for Li_6PS_5Cl with 50% anion disorder. **b**, Li-ion diffusion barriers in Li_6PS_5Cl as a function of anion disorder. GB widths of 1.5 nm and 2.5 nm yield similar results and are consistent with experimental values with uncertainties (Adeli et al. [10], Schlenker et al. [11], Ding et al. [15]), whereas a width of 1 nm leads to overestimated barriers. **c**, GB diffusivities at 600 K estimated using different GB widths. **d**, Macroscopic diffusivities of 50% anion-disordered Li_6PS_5Cl polycrystals at 600 K. The use of smaller GB widths of 1.5 and 1 nm leads to inconsistencies between all-atom atomistic and continuum-scale predictions.

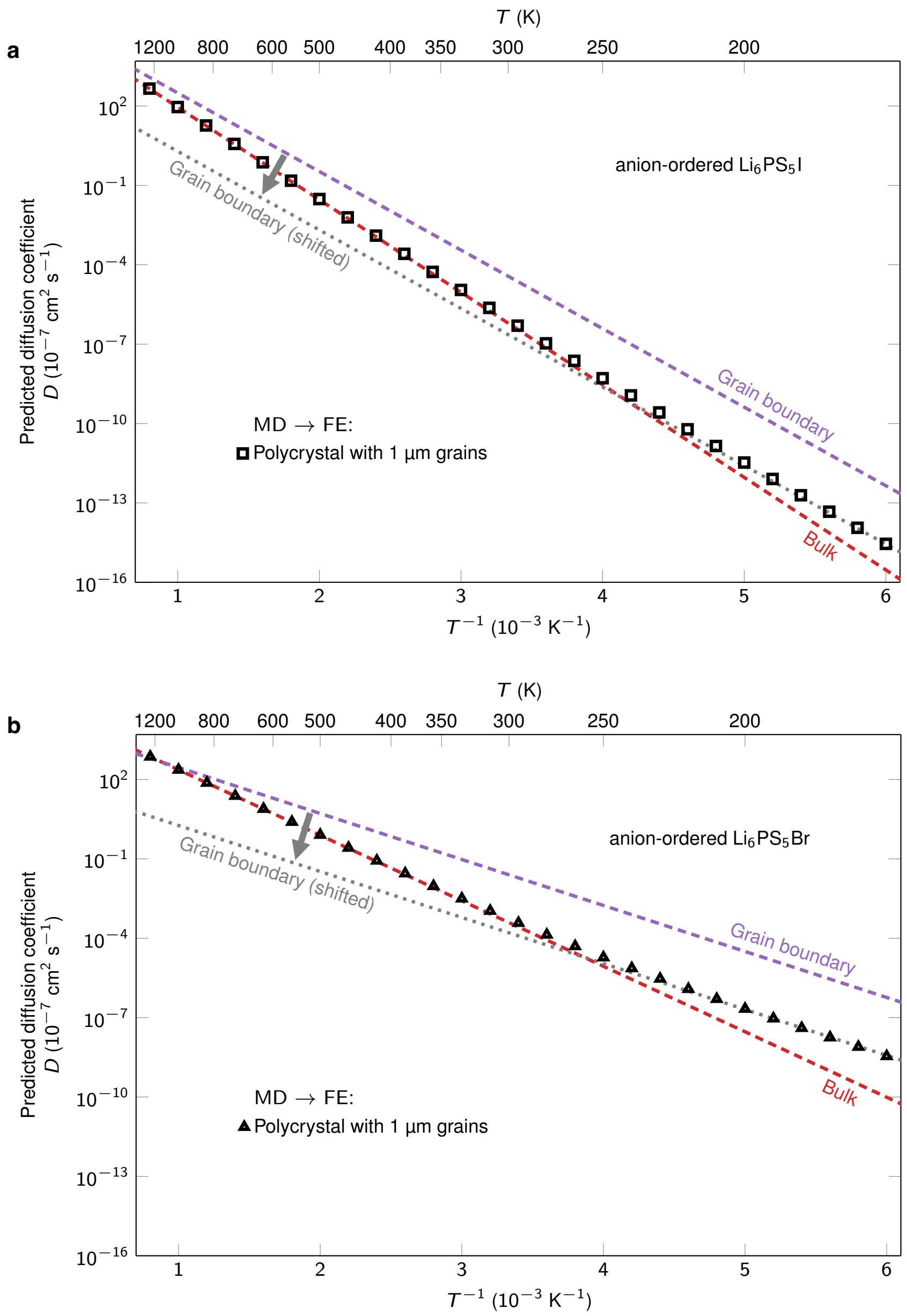

Suppl. Fig. 13. **Non-Arrhenius diffusion behavior arising from grain boundaries in anion-ordered argyrodites.** Temperature-dependent diffusivities of, **a**, anion-ordered Li_6PS_5I and, **b**, anion-ordered Li_6PS_5Br. Polycrystalline models with 1 µm-sized grains are used for demonstration. Dashed lines are obtained from Arrhenius fits to bulk and grain-boundary diffusivities. Additional dotted lines, shifted from the grain-boundary Arrhenius fits, highlight the grain-boundary-dominated regime at low temperatures. The non-Arrhenius behavior depends on the ratio of bulk to grain-boundary diffusivities and its temperature dependence.

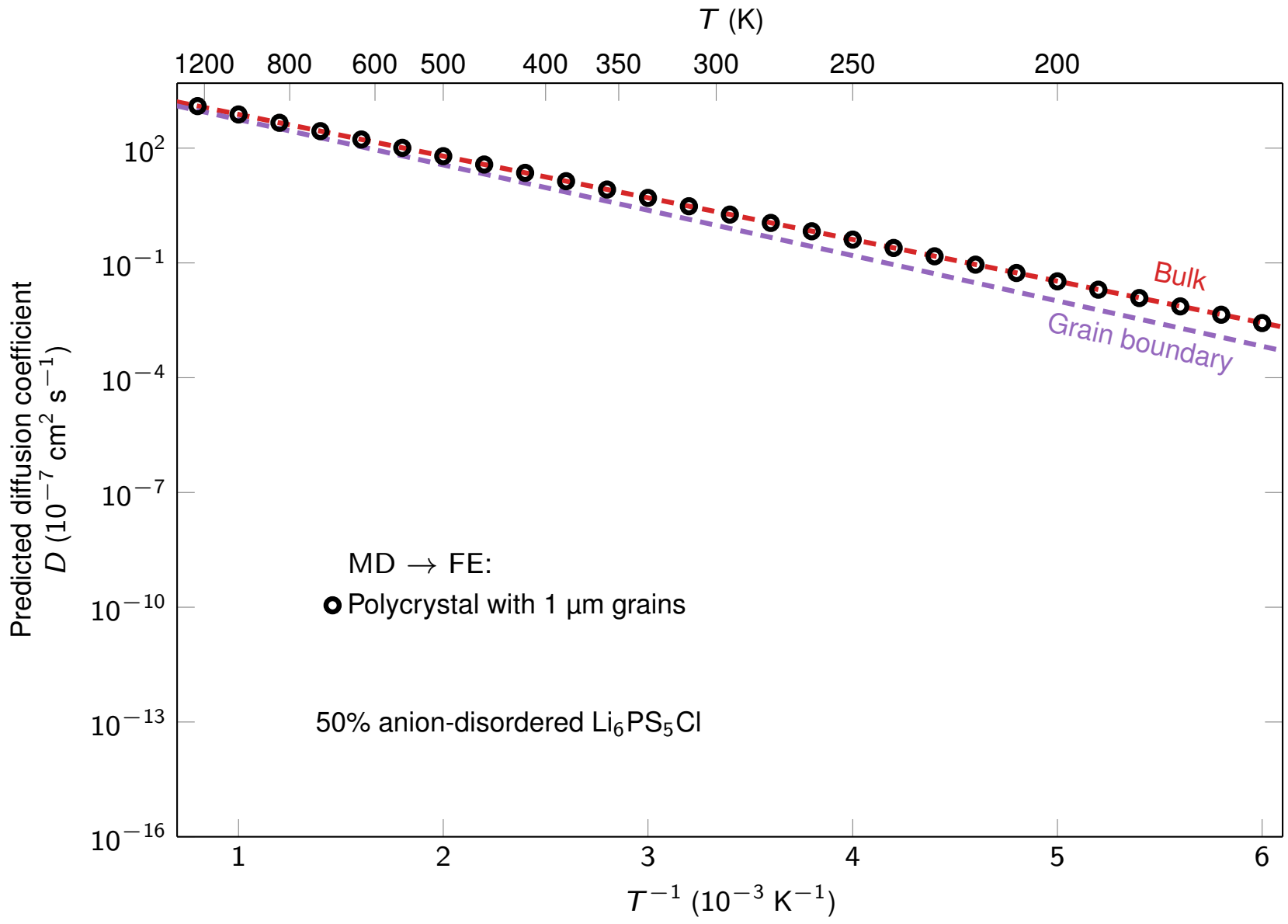

Suppl. Fig. 14. **Diffusion behavior of polycrystalline anion-disordered argyrodites.** Results from 50% anion-disordered Li_6PS_5Cl polycrystalline model are exemplified. Dashed lines are obtained from Arrhenius fits to bulk and grain-boundary diffusivities. Because bulk and grain-boundary diffusivities are comparable, the influence of grain boundaries is minimal, and the polycrystalline diffusivity closely follows the bulk Arrhenius relation. Non-Arrhenius behavior is not clearly observed within the investigated temperature range.

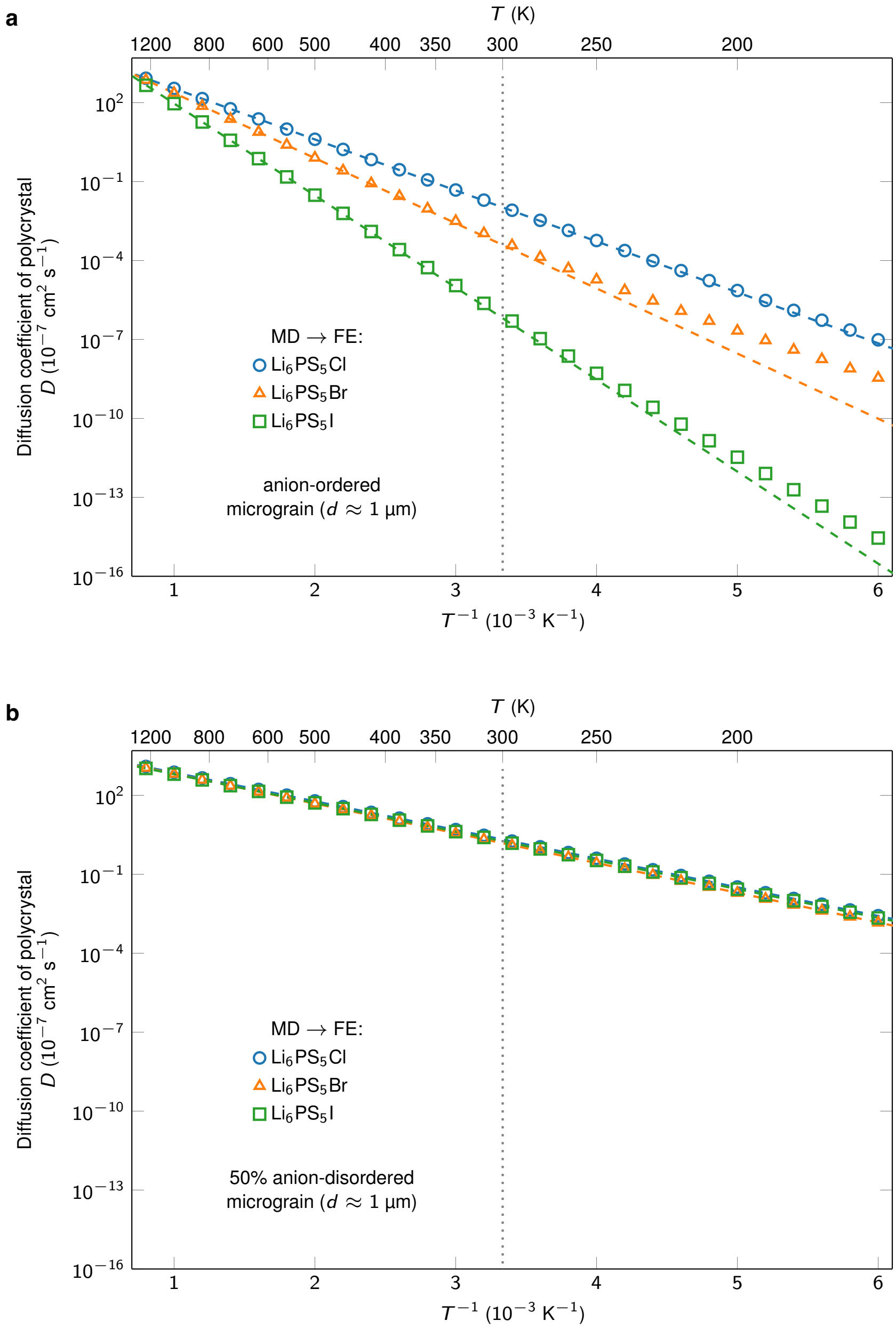

Suppl. Fig. 15. **Comparison of predicted temperature-dependent diffusivities of polycrystalline Li_6PS_5X with $X \in$ {Cl, Br, I}.** **a**, Without anion disorder. The non-Arrhenius diffusion behavior is observed for Li_6PS_5Br and Li_6PS_5I, but cannot be clearly observed for Li_6PS_5Cl in the presented temperature range. **b**, With anion disorder. The non-Arrhenius diffusion behavior cannot be clearly observed for 50% disordered Li_6PS_5X with $X \in \{\mathrm{Cl}, \mathrm{Br}, \mathrm{I}\}$ in the presented temperature range.

Suppl. Table 3. **Timeline of computational approaches for investigating Li-ion diffusion in inorganic solid electrolytes with grain boundaries (GBs).** Molecular dynamics simulations with interatomic potentials were performed in these studies. Yes or - indicates whether the corresponding model was considered in the given study. Since 2022, machine-learning potentials have gained increasing popularity.

Year	Interatomic potential[a]	Solid electrolyte[b]	Single-type GBs[c]	Polycrystal[d]	Reference
2017	pairwise	$Li_7La_3Zr_2O_{12}$	$\Sigma3$, $\Sigma5$	-	[22]
2018	pairwise	Li_3OCl	$\Sigma3$, $\Sigma5$	-	[23]
2018	pairwise	$Li_7La_3Zr_2O_{12}$	$\Sigma3$, $\Sigma5$, $\Sigma7$, $\Sigma9$, $\Sigma11$	-	[24]
2018	pairwise	$Li_7La_3Zr_2O_{12}$	$\Sigma5$	-	[25]
2021	pairwise	$Li_{0.16}La_{0.62}TiO_3$	$\Sigma2$, $\Sigma3$, $\Sigma5$	-	[26]
2021	pairwise + angular	$LiZr_2(PO_4)_3$	-	yes	[27]
2021	pairwise + angular	$LiZr_2(PO_4)_3$	$\Sigma3$, $\Sigma5$, $\Sigma7$	-	[28]
2021	pairwise	$Li_{6.25}La_3Zr_2O_{12}$	-	partially[e]	[6]
2022	pairwise	$Li_{10}GeP_2S_{12}$	-	yes	[29]
2022	NNP	$Li_7La_3Zr_2O_{12}$	$\Sigma5$	-	[30]
2022	pairwise	Li_3OCl, Li_3OBr	-	yes	[31]
2023	MTP	$Li_{0.375}Sr_{0.4375}Ta_{0.75}Zr_{0.25}O_3$	$\Sigma3$, $\Sigma5$, $\Sigma51$	-	[32]
2023	MTP	β-Li_3PS_4	$\Sigma3$, $\Sigma5$, $\Sigma13$, $\Sigma25$	-	[33]
2024	MTP	Li_6PS_5Cl	$\Sigma5$	-	[34]
2024	pairwise	$Li_{6.75}La_3Zr_{1.75}Nb_{0.25}O_{12}$	$\Sigma3$, $\Sigma5$, $\Sigma7$, $\Sigma9$, $\Sigma11$	-	[35]
2024	pairwise	$Li_2Ti_6O_{13}$, $Li_2Zr_6O_{13}$	-	yes	[36]
2024	pairwise	$Li_{0.3125}La_{0.5625}TiO_3$	$\Sigma5$	-	[37]
2024	MTP	Li_6PS_5Cl	$\Sigma3$, $\Sigma5$	-	[1]
2025	ReaxFF[f]	$Li_{1.3}Al_{0.3}Ti_{1.7}(PO_4)_3$	-	yes	[39]
2025	DP	LiCl[g]	-	yes	[40]
2026	MTP	Li_6PS_5Cl, Li_6PS_5Br, Li_6PS_5I	$\Sigma3$, $\cdots$, $\Sigma51$, $\cdots$, $\Sigma99$[h]	yes	this work

[a] The pairwise potentials include the Morse, the Buckingham, and the Lennard–Jones forms, some combined with a Coulombic term. The angular potential includes the Stillinger–Weber potential. Machine-learning potentials, such as the neural network potential (NNP) [16, 17], the moment tensor potential (MTP) [2, 18, 19], and the deep potential (DP) [20] are highlighted.

[b] The sulfide-based solid electrolytes are highlighted.

[c] The notation with Σ indicates the degree of misorientation of the GBs [21].

[d] Random GBs and triple junction structures are included in the polycrystalline simulation model.

[e] Diffusivities of amorphous phases from atomic simulations were used for GBs in a phase field model to simulate polycrystals.

[f] The reactive force field (ReaxFF) developed in Ref. [38] was used.

[g] LiCl forms at the solid electrolyte interphase of chloride-based solid electrolytes, such as Li_6PS_5Cl [40, 41].

[h] To our knowledge, $\Sigma99$ is the single-type GB with the smallest misorientation angle (11.54°) that has been investigated in solid electrolytes to date.

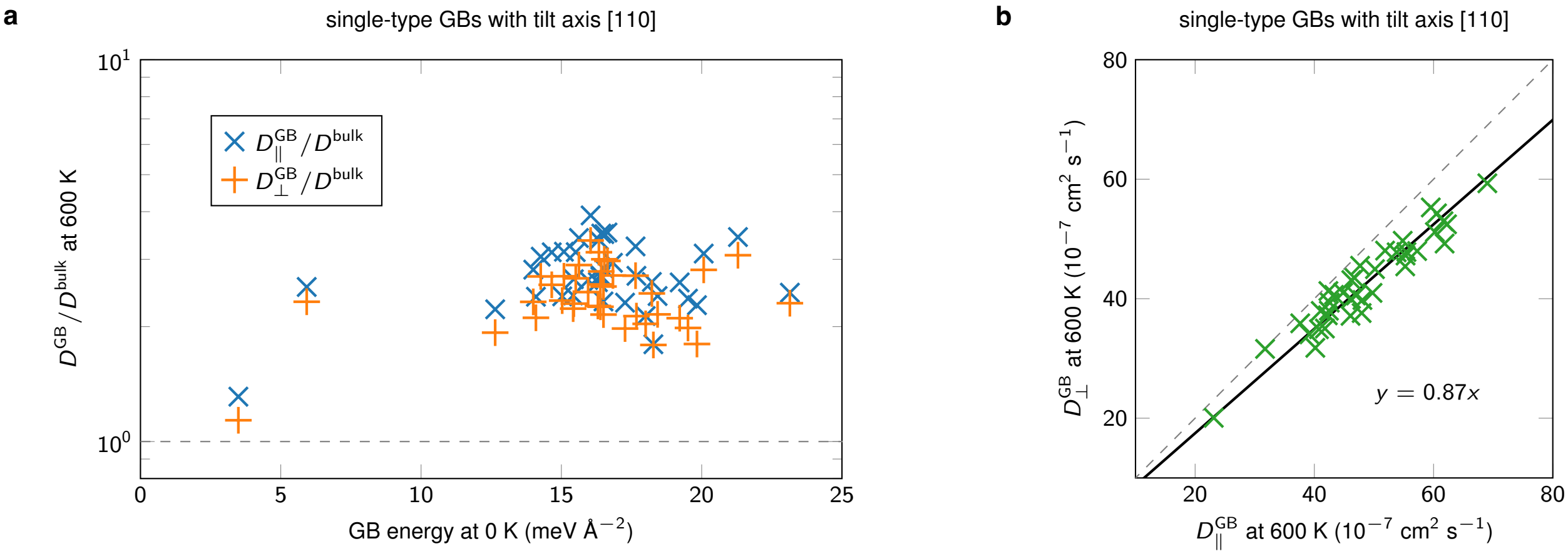

Suppl. Fig. 16. **Analysis of Li-ion diffusivity in different single-type grain boundaries (GBs) of anion-ordered Li_6PS_5Cl.** Tilt GBs with a [110] rotation axis, ranging from Σ3 to Σ99 and covering misorientation angles from 0 to 180°, are included. **a**, Correlation between GB energy at 0 K and GB to bulk diffusivity ratio at 600 K. GB diffusivities are decomposed into components parallel (∥) and perpendicular (⊥) to the GB plane. The Borisov relation [42] is not statistically supported by the present data. **b**, Correlation between Li-ion diffusivity parallel and perpendicular to the GB plane. GBs in Li_6PS_5Cl show weak anisotropy in diffusivity, with the perpendicular component reaching ∼87% of the parallel value at 600 K.

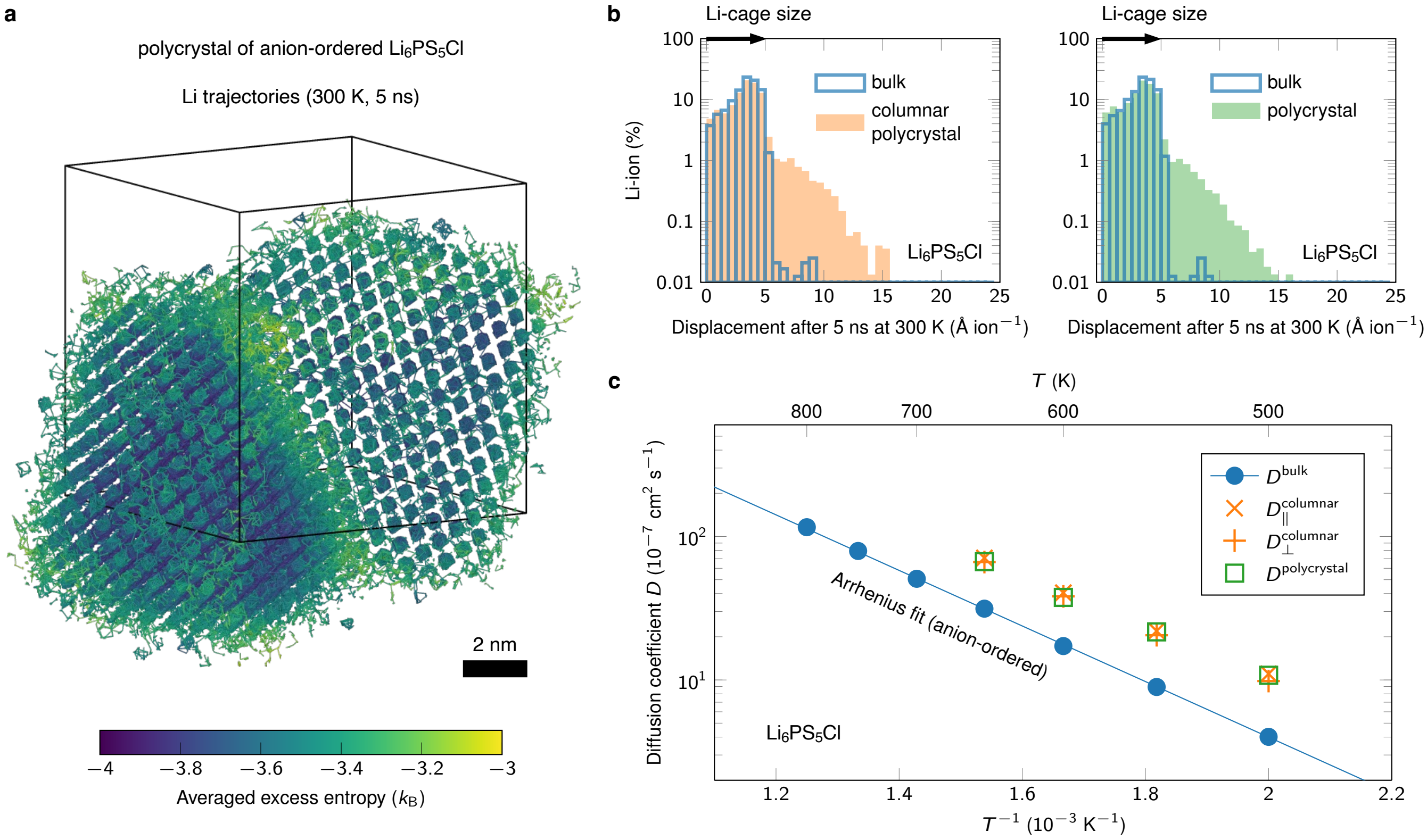

Suppl. Fig. 17. **Comparison of Li-ion diffusivity in anion-ordered Li_6PS_5Cl between columnar and polycrystalline models.** **a**, Trajectories of Li ions in a polycrystalline model after molecular dynamics simulations at 300 K for 5 ns. Grain-boundary regions enclosing the grains exhibit elevated excess entropy, reflecting structural disorder. **b**, Comparison of Li-ion displacement distributions in bulk, columnar, and polycrystalline models after 5 ns of molecular dynamics simulations at 300 K. The Li cage is ∼5 Å in size. Similar enhancement of inter-cage diffusion, indicated by Li-ion displacements exceeding 10 Å, is observed in both columnar and polycrystalline models. **c**, Comparison of Li-ion diffusion coefficients calculated for all Li atoms in bulk, columnar, and polycrystalline models. The number of Li atoms is comparable in these models, and Li ions exhibit similar diffusion coefficients in columnar and polycrystalline models. In the columnar model, Li-ion diffusion parallel (∥) to the grain-boundary plane is comparable to diffusion perpendicular (⊥) to it.

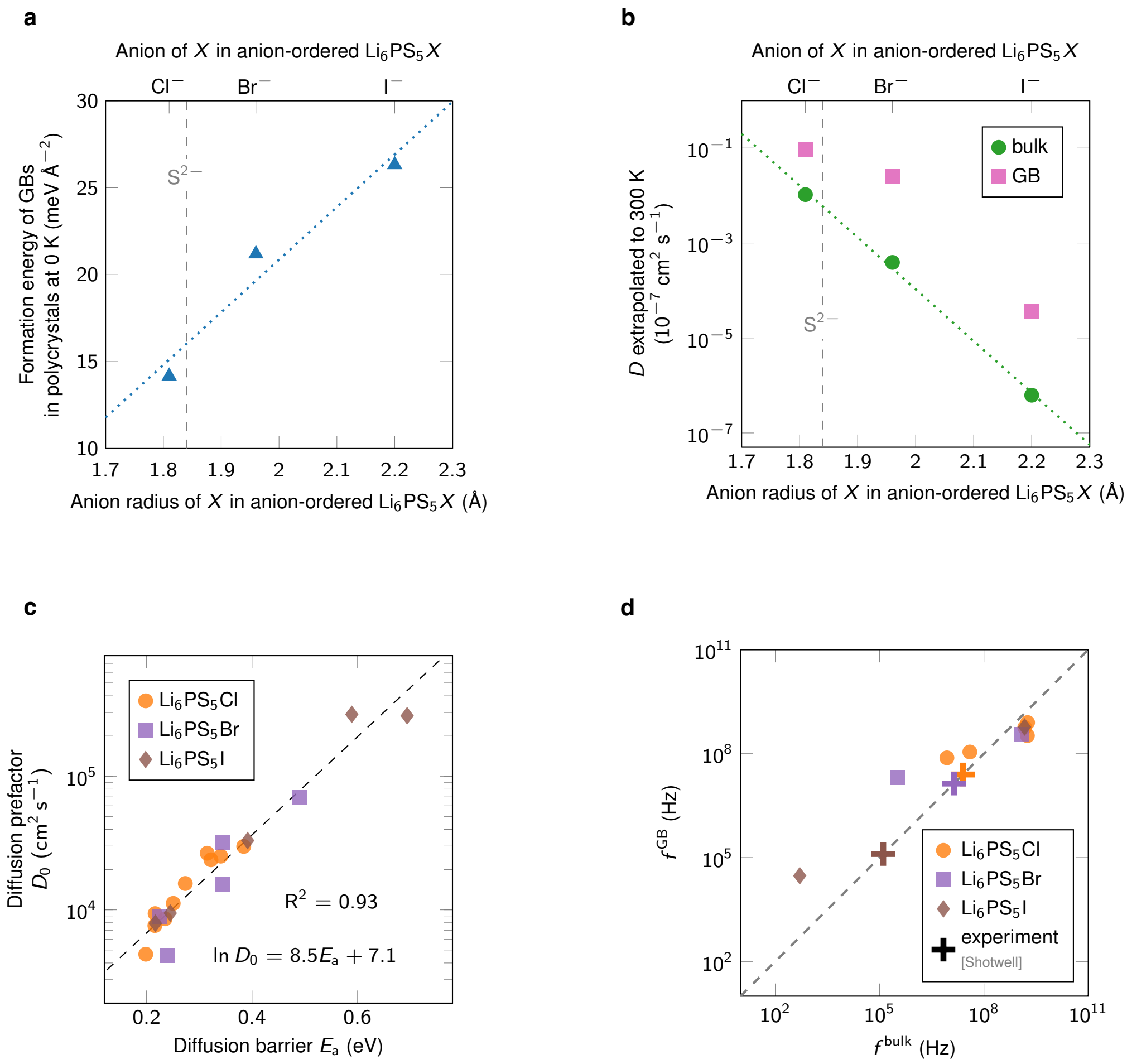

Suppl. Fig. 18. **Grain-boundary (GB) effects in argyrodite solid electrolytes, Li_6PS_5X with $X \in$ {Cl, Br, I}. a**, Impact of the halide anion on GB formation energy at 0 K. The GBs are embedded in polycrystalline models with random grain orientations. The ionic radius of S^{2-} is similar to that of Cl^-. GB formation energy positively correlates with the halide anion radius in argyrodites. **b**, Predicted diffusion coefficients extrapolated to 300 K based on Arrhenius fitting for bulk and GBs in argyrodites. The GB values (averaged over all GBs in polycrystal) were extracted from polycrystalline models. Diffusivity decreases with increasing anion radius of the halide species. The GB-induced cage-opening effect enhances Li-ion diffusivity in all investigated argyrodites. **c**, Correlation between the diffusion prefactor D_0 and diffusion barrier E_a obtained from Arrhenius fitting. The Meyer–Neldel rule [43] is verified for argyrodites with structural (bulk and GB) and chemical (anion disorder) variations. **d**, Correlation of characteristic Li-ion hopping frequencies f between the bulk and GBs. Experimental data from Shotwell et al. [8], which include contributions from both bulk and GBs, are shown assuming $f^{\text{bulk}} = f^{\text{GB}}$ for visual comparison.

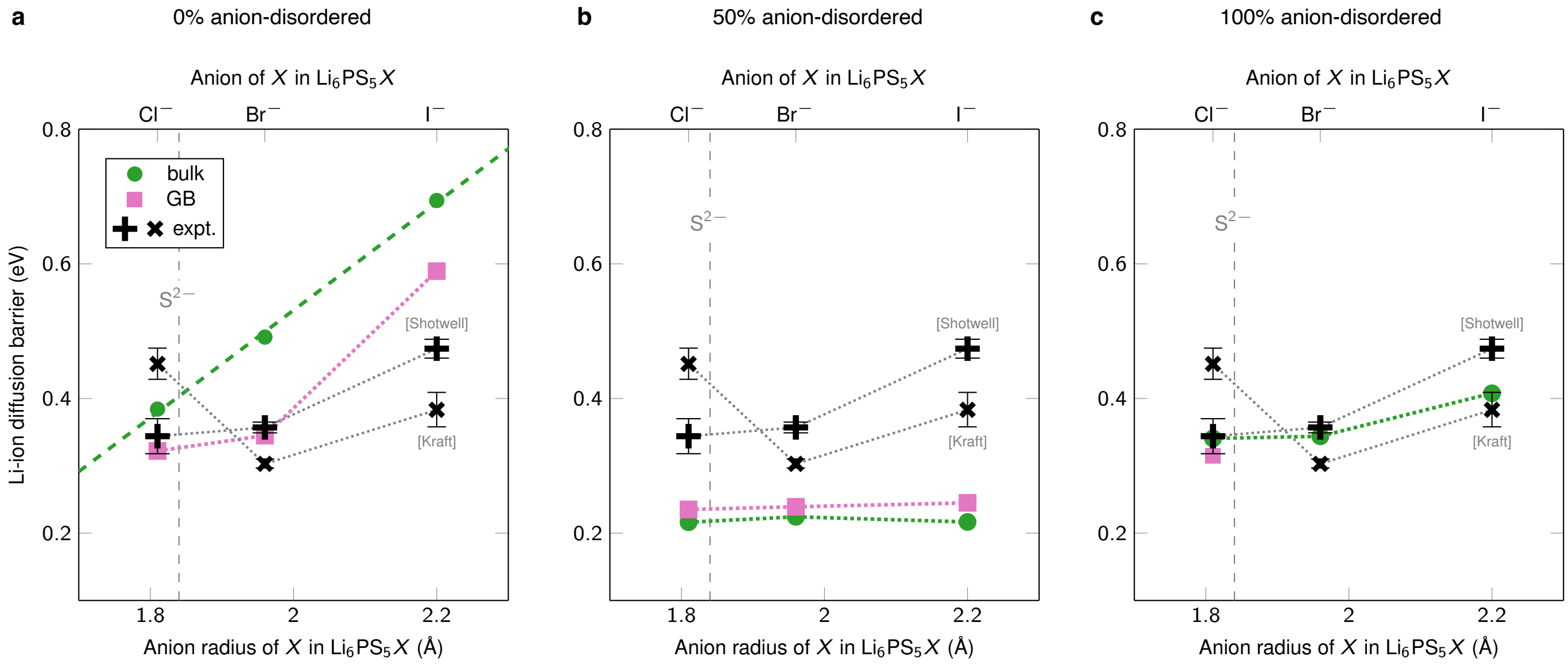

Suppl. Fig. 19. **Influence of halide species on Li-ion diffusion barriers for argyrodites with different anion disorder.** Results are shown for Li_6PS_5X with $X \in \mathrm{Cl}, \mathrm{Br}, \mathrm{I}$, with (**a**) 0%, (**b**) 50%, and (**c**) 100% anion disorder. Consistency is observed across all argyrodites for the activation energy as a function of anion disorder. The bulk diffusion barriers decrease from 0% to 50% anion disorder and then increase from 50% to 100%. Grain boundaries hinder Li-ion diffusion at 50% anion disorder, while they enhance diffusion at 0%. The Li-ion diffusion barrier increases linearly with anion radius in anion-ordered bulk argyrodites, while exhibiting more complex behavior in other cases. Experimental values and uncertainties from electrochemical impedance spectroscopy measurements reported by Shotwell et al. [8] and Kraft et al. [44] are compared.

Suppl. Table 4. **Predicted Li-ion self-diffusivities and derived quantities of argyrodite solid electrolytes at 300 K.** The anion disorder row indicates the degree of X/S-anion disorder. Grain boundary (GB) diffusion coefficients D^{GB} are from MD simulations of polycrystals, representing averages over all GB structures within them. The GB-to-bulk ratio quantifies the impact of GBs on diffusion in the polycrystal with ↑ and ↓ for increase and decrease, respectively. The f rows show the predicted characteristic Li-ion hopping frequencies, which are consistent with experimental values from Shotwell et al. [8].

System	Li_6PS_5Cl				
Anion disorder (%)	0	25	50	75	100
D^{bulk} (10^{-7} cm^{2}s^{-1})	0.010	2.153	2.184	1.808	0.048
D^{GB} (10^{-7} cm^{2}s^{-1})	0.092	0.401	0.959	0.690	0.136
$D^{\mathrm{GB}}/D^{\mathrm{bulk}}$	8.79 ↑	0.19 ↓	0.44 ↓	0.38 ↓	2.85 ↑
f^{bulk} (Hz)	8.61×10^6	1.77×10^9	1.80×10^9	1.49×10^9	3.92×10^7
f^{GB} (Hz)	7.57×10^7	3.30×10^8	7.90×10^8	5.68×10^8	1.12×10^8
$f^{\mathrm{experiment}}$ (Hz) [Shotwell]	2.50×10^7				

System	Li_6PS_5Br		
Anion disorder (%)	0	50	100
D^{bulk} (10^{-7} cm^{2}s^{-1})	3.9×10^{-4}	1.522	0.053
D^{GB} (10^{-7} cm^{2}s^{-1})	0.025	0.436	-
$D^{\mathrm{GB}}/D^{\mathrm{bulk}}$	64.06 ↑	0.29 ↓	-
f^{bulk} (Hz)	3.19×10^5	1.25×10^9	4.38×10^7
f^{GB} (Hz)	2.04×10^7	3.57×10^8	-
$f^{\mathrm{experiment}}$ (Hz) [Shotwell]	1.37×10^7		

System	Li_6PS_5I		
Anion disorder (%)	0	50	100
D^{bulk} (10^{-7} cm^{2}s^{-1})	6.2×10^{-7}	1.817	4.1×10^{-3}
D^{GB} (10^{-7} cm^{2}s^{-1})	3.7×10^{-5}	0.724	-
$D^{\mathrm{GB}}/D^{\mathrm{bulk}}$	59.25 ↑	0.40 ↓	-
f^{bulk} (Hz)	5.05×10^2	1.48×10^9	3.31×10^6
f^{GB} (Hz)	2.99×10^4	5.88×10^8	-
$f^{\mathrm{experiment}}$ (Hz) [Shotwell]	1.27×10^5		

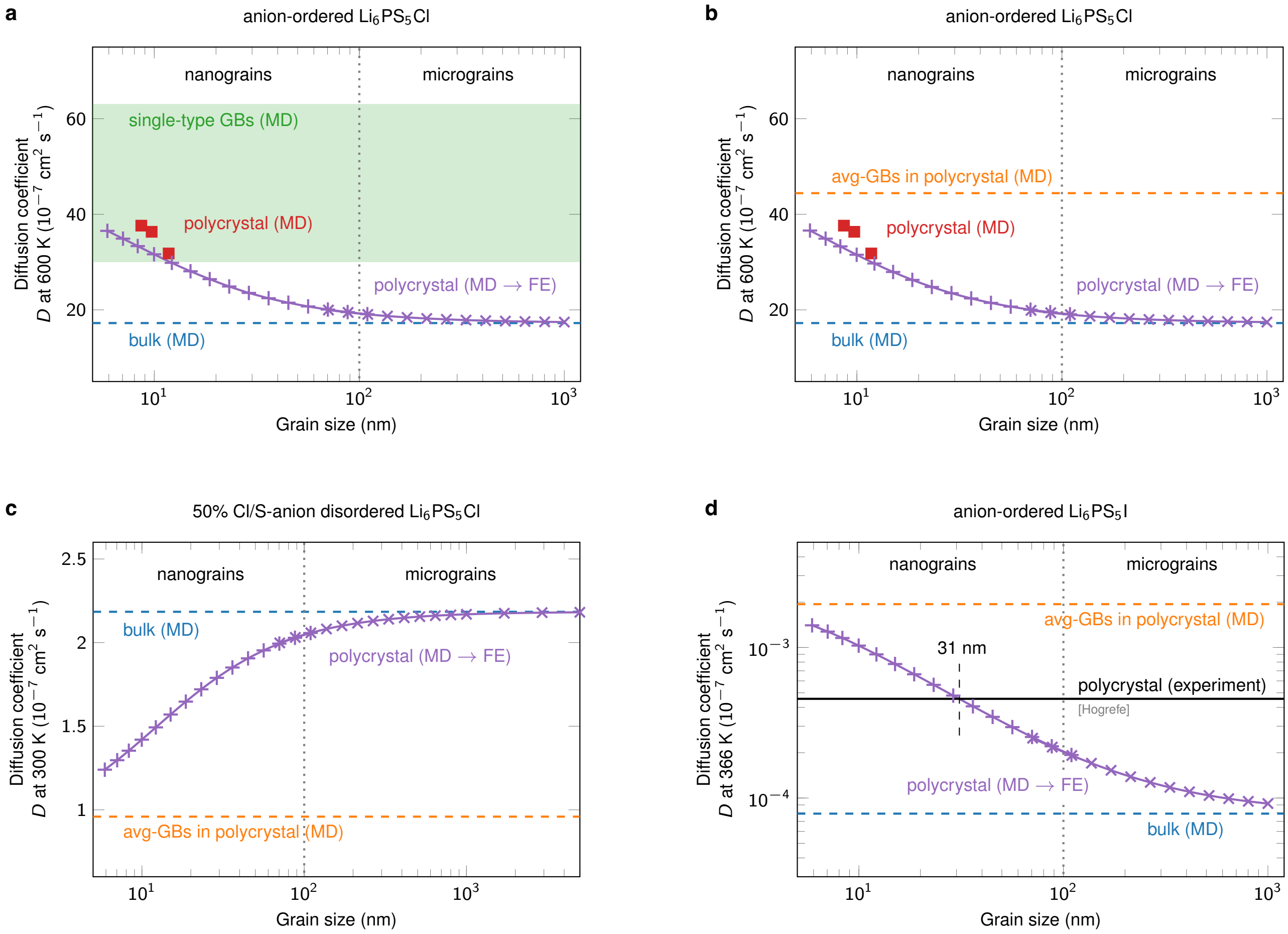

Suppl. Fig. 20. **Predicted Li-ion diffusivities in polycrystalline argyrodite solid electrolytes vs. grain size.** **a**, Anisotropic diffusivities of randomly selected single-type grain boundaries (GBs) are used as input for finite element (FE) simulations. Tests show that deviations arising from the choice of GB types are negligible at the macroscopic scale, owing to the small differences in diffusivity among single-type GBs. Total diffusivities obtained from polycrystalline atomistic models demonstrate the consistency between atomistic and continuum simulations. **b**, GB diffusivity extracted from polycrystalline models (avg-GBs in polycrystal) is used as input for FE simulations. The macroscopic diffusivities from MD and from the MD → FE simulations for anion-ordered Li_6PS_5Cl shown in **a** and **b** are in excellent agreement, confirming that using avg-GBs values as inputs for FE simulations is appropriate. **c**, Macroscopic diffusivity of Li_6PS_5Cl with 50% Cl/S-anion disorder at 300 K. Diffusivity of the bulk structure is larger than that of GBs. Large changes in diffusivity are predicted for polycrystals with nanograins ($d < 100$ nm). **d**, Macroscopic diffusivity of anion-ordered Li_6PS_5I at 366 K. Experimental diffusivity can be reproduced with a polycrystalline microstructure having an average grain size of about 31 nm.

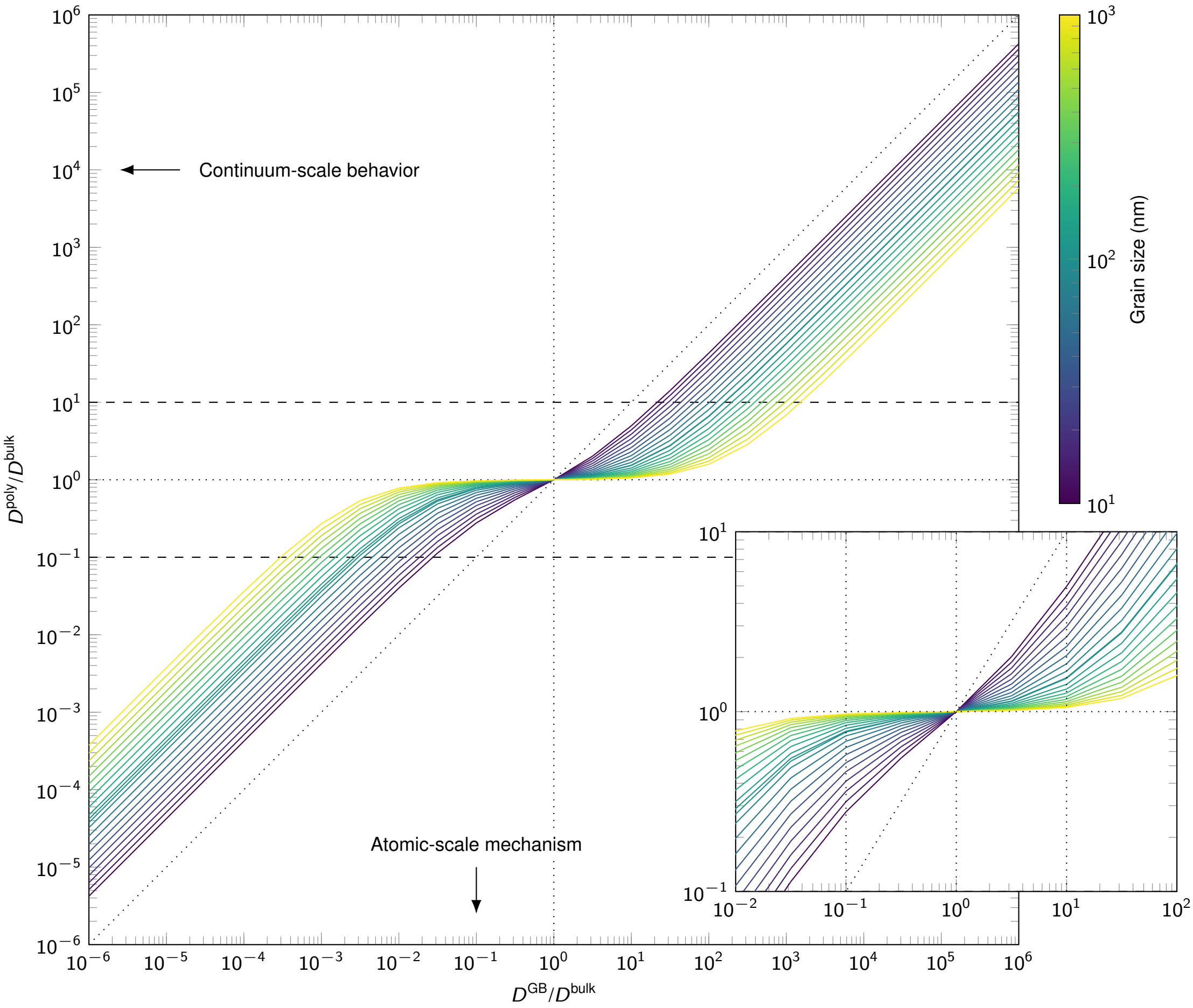

Suppl. Fig. 21. **Precomputed map linking atomic-scale mechanisms to continuum-scale behavior in general polycrystalline solid electrolytes.** In a polycrystalline model with isotropic grain boundaries (GBs), the x-axis indicates the assumed GB diffusivity relative to the bulk, while the y-axis shows the resulting macroscopic diffusivity of the polycrystal relative to the bulk. The line color represents the average grain size of the polycrystalline model, with the GB width of 2.5 nm. Dashed lines indicate a one-order-of-magnitude change in macroscopic diffusivity. The inset shows an enlarged section of the precomputed map.

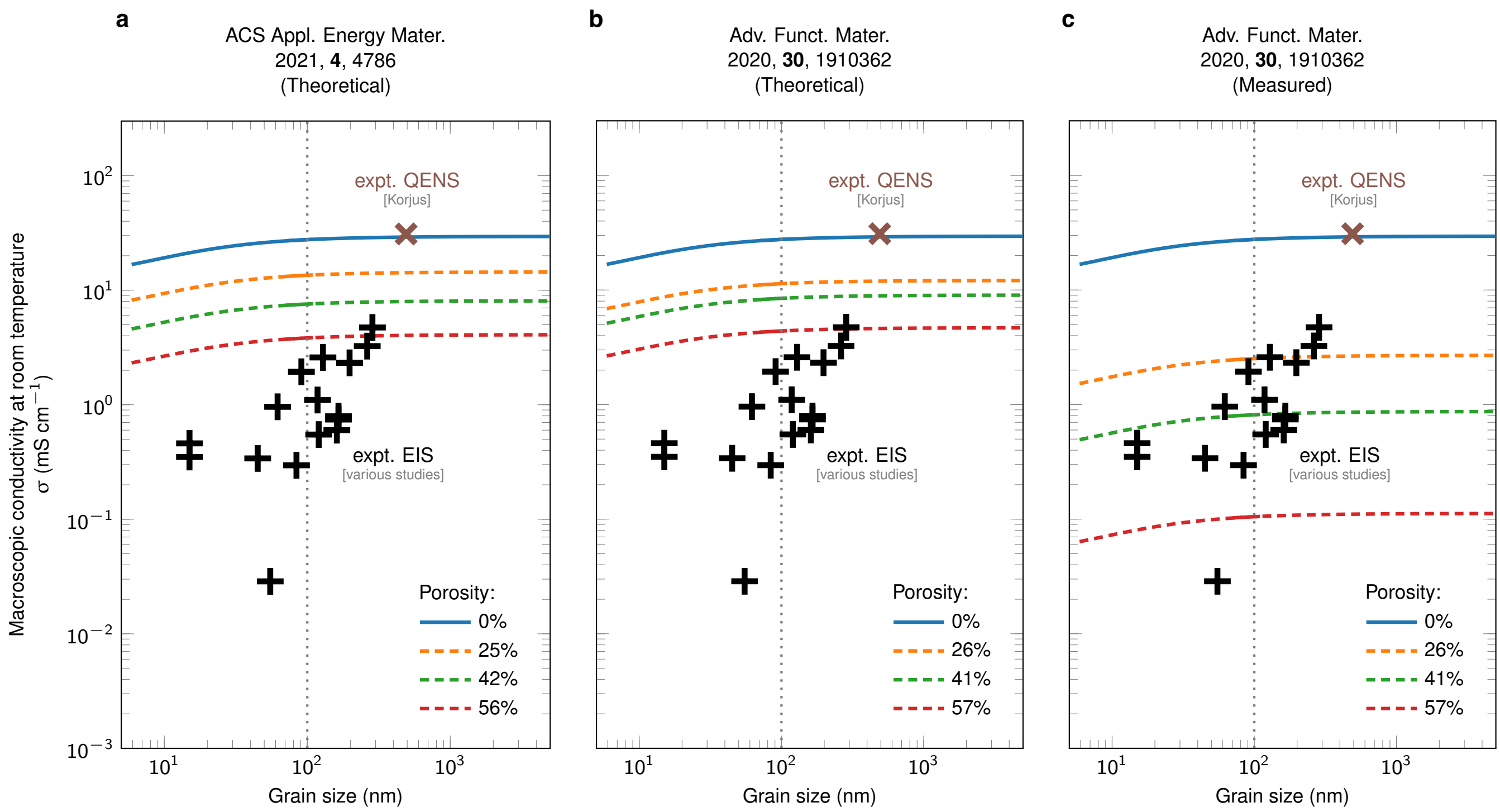

Suppl. Fig. 22. **Impact of porosity on macroscopic conductivity of solid electrolytes at 300 K.** Li_6PS_5Cl with 50% anion disorder is considered. The grain-size effect is estimated for a close-contact polycrystal using our multiscale modeling framework, while the porosity effect is applied as a shift on top of these close-contact results, based on theoretical models (ACS Appl. Energy Mater. [45] and Adv. Funct. Mater. [46]) and experimental measurements [46]. Accounting for porosity reduces the macroscopic conductivity by one to two orders of magnitude, bringing predictions for anion-disordered Li_6PS_5Cl closer to experimental observations.

Supplementary references

[1] Y. Ou, Y. Ikeda, L. Scholz, S. Divinski, F. Fritzen, and B. Grabowski, Phys. Rev. Mater. **8**, 115407 (2024).
[2] E. Podryabinkin, K. Garifullin, A. Shapeev, and I. Novikov, J. Chem. Phys. **159**, 084112 (2023).
[3] B. D. Lee, J.-W. Lee, J. Park, M. Y. Cho, W. B. Park, and K.-S. Sohn, RSC Adv. **12**, 31156 (2022).
[4] M. Jiang, Z.-W. Chen, A. Rao, L.-X. Chen, X.-T. Zu, and C. V. Singh, J. Mater. Chem. C **10**, 18294 (2022).
[5] T. Jeon, G. H. Cha, and S. C. Jung, J. Mater. Chem. A **12**, 993 (2024).
[6] T. W. Heo, A. Grieder, B. Wang, M. Wood, T. Hsu, S. A. Akhade, L. F. Wan, L.-Q. Chen, N. Adelstein, and B. C. Wood, npj Comput. Mater. **7**, 1 (2021).
[7] M. Brinek, C. Hiebl, and H. M. R. Wilkening, Chem. Mater. **32**, 4754 (2020).
[8] A. M. Shotwell, M. C. Schulze, P. Yox, C. Alaniz, and A. E. Maughan, Adv. Funct. Mater. **35**, 2500237 (2025).
[9] S. De, A. P. Bartók, G. Csányi, and M. Ceriotti, Phys. Chem. Chem. Phys. **18**, 13754 (2016).
[10] P. Adeli, J. D. Bazak, K. H. Park, I. Kochetkov, A. Huq, G. R. Goward, and L. F. Nazar, Angew. Chem. Int. Ed. **58**, 8681 (2019).
[11] R. Schlenker, A.-L. Hansen, A. Senyshyn, T. Zinkevich, M. Knapp, T. Hupfer, H. Ehrenberg, and S. Indris, Chem. Mater. **32**, 8420 (2020).
[12] I. Hanghofer, M. Brinek, S. L. Eisbacher, B. Bitschnau, M. Volck, V. Hennige, I. Hanzu, D. Rettenwander, and H. M. R. Wilkening, Phys. Chem. Chem. Phys. **21**, 8489 (2019).
[13] H.-J. Deiseroth, J. Maier, K. Weichert, V. Nickel, S.-T. Kong, and C. Reiner, Z. Anorg. Allg. Chem. **637**, 1287 (2011).
[14] O. Korjus, S. Mitra, Q. Berrod, V. Vanpeene, M. Appel, L. Broche, S. Lyonnard, and C. Villevieille, Energy Storage Mater. **82**, 104589 (2025).
[15] J. Ding, M. K. Gupta, C. Rosenbach, H.-M. Lin, N. C. Osti, D. L. Abernathy, W. G. Zeier, and O. Delaire, Nat. Phys. **21**, 118 (2025).
[16] A. Singraber, T. Morawietz, J. Behler, and C. Dellago, J. Chem. Theory Comput. **15**, 3075 (2019).
[17] A. Singraber, J. Behler, and C. Dellago, J. Chem. Theory Comput. **15**, 1827 (2019).
[18] A. V. Shapeev, Multiscale Model. & Simul. **14**, 1153 (2016).
[19] I. S. Novikov, K. Gubaev, E. V. Podryabinkin, and A. V. Shapeev, Mach. Learn.: Sci. Technol. **2**, 025002 (2020).
[20] H. Wang, L. Zhang, J. Han, and W. E, Comput. Phys. Commun. **228**, 178 (2018).
[21] H. Grimmer, W. Bollmann, and D. H. Warrington, Acta Crystallogr. A **30**, 197 (1974).
[22] S. Yu and D. J. Siegel, Chem. Mater. **29**, 9639 (2017).
[23] J. A. Dawson, P. Canepa, T. Famprikis, C. Masquelier, and M. S. Islam, J. Am. Chem. Soc. **140**, 362 (2018).
[24] H. Shiiba, N. Zettsu, M. Yamashita, H. Onodera, R. Jalem, M. Nakayama, and K. Teshima, J. Phys. Chem. C **122**, 21755 (2018).
[25] S. Yu and D. J. Siegel, ACS Appl. Mater. Interfaces **10**, 38151 (2018).
[26] A. R. Symington, M. Molinari, J. A. Dawson, J. M. Statham, J. Purton, P. Canepa, and S. C. Parker, J. Mater. Chem. A **9**, 6487 (2021).
[27] R. Kobayashi, K. Nakano, and M. Nakayama, Acta Mater. **226**, 117596 (2022).
[28] K. Nakano, N. Tanibata, H. Takeda, R. Kobayashi, M. Nakayama, and N. Watanabe, J. Phys. Chem. C **125**, 23604 (2021).
[29] J. A. Dawson and M. S. Islam, ACS Mater. Lett. **4**, 424 (2022).
[30] K. Kim, A. Dive, A. Grieder, N. Adelstein, S. Kang, L. F. Wan, and B. C. Wood, J. Chem. Phys. **156**, 221101 (2022).
[31] L. Van Duong, M. T. Nguyen, and Y. A. Zulueta, RSC Adv. **12**, 20029 (2022).
[32] T. Lee, J. Qi, C. A. Gadre, H. Huyan, S.-T. Ko, Y. Zuo, C. Du, J. Li, T. Aoki, R. Wu, J. Luo, S. P. Ong, and X. Pan, Nat. Commun. **14**, 1 (2023).
[33] R. Jalem, M. L. H. Chandrappa, J. Qi, Y. Tateyama, and S. P. Ong, Energy Adv. **2**, 2029 (2023).
[34] J. H. Kim, B. Jun, Y. J. Jang, S. H. Choi, S. H. Choi, S. M. Cho, Y.-G. Kim, B.-H. Kim, and S. U. Lee, Nano Energy **124**, 109436 (2024).
[35] H. Shiiba, M. Koyama, N. Zettsu, and K. Teshima, Chem. Mater. **36**, 6370 (2024).
[36] Y. A. Zulueta, M. T. Nguyen, and M. P. Pham-Ho, RSC Adv. **14**, 22974 (2024).
[37] J. C. M. Madrid, A. Jonderian, E. McCalla, and K. K. Ghuman, ACS Appl. Energy Mater. **7**, 8342 (2024).
[38] Y. K. Shin, M. Y. Sengul, A. S. M. Jonayat, W. Lee, E. D. Gomez, C. A. Randall, and A. C. T. v. Duin, Phys. Chem. Chem. Phys. **20**, 22134 (2018).
[39] S. Ghosh, S. Nandy, A. Valalahally Gopala, T. K. Patra, K. H. Chae, B. R. K. Nanda, and C. Sudakar, ACS Appl. Mater. Interfaces **17**, 9460 (2025).
[40] Y. Kim, Mater. Lett. **389**, 138336 (2025).
[41] Y. Li, W. Arnold, J. B. Jasinski, A. Thapa, G. Sumanasekera, M. Sunkara, B. Narayanan, T. Druffel, and H. Wang, Electrochim. Acta **363**, 137128 (2020).
[42] J. Pelleg, Philos. Mag.-J. Theor. Exp. Appl. Phys. **14**, 595 (1966).
[43] R. Metselaar and G. Oversluizen, J. Solid State Chem. **55**, 320 (1984).
[44] M. A. Kraft, S. P. Culver, M. Calderon, F. Böcher, T. Krauskopf, A. Senyshyn, C. Dietrich, A. Zevalkink, J. Janek, and W. G. Zeier, J. Am. Chem. Soc. **139**, 10909 (2017).
[45] A. Neumann, T. R. Hamann, T. Danner, S. Hein, K. Becker-Steinberger, E. Wachsman, and A. Latz, ACS Appl. Energy Mater. **4**, 4786 (2021).
[46] T. Hamann, L. Zhang, Y. Gong, G. Godbey, J. Gritton, D. McOwen, G. Hitz, and E. Wachsman, Adv. Funct. Mater. **30**, 1910362 (2020).
