# Supplementary material for: Microstructural insights into fast ion transport in solid electrolytes via multiscale modeling
Source: Nat Commun. 2026 Aug 20;17:8726. doi: 10.1038/s41467-026-76216-w (PMC13493929; doi:10.1038/s41467-026-76216-w)
Supplement: Supplementary file 2 [file 41467_2026_76216_MOESM2_ESM.pdf]

nature portfolio

Peer Review File

Microstructural Insights into Fast Ion Transport in Solid Electrolytes via Multiscale Modeling

Corresponding Author: Mr Yongliang Ou

This file contains all reviewer reports in order by version, followed by all author rebuttals in order by version.

Version 0:

Reviewer comments:

Reviewer #1

(Remarks to the Author)
This study used multiscale modeling to gain microstructural insights into fast ion transport in argyrodite Li_6PS_5X (X = Cl, Br, and I) solid electrolytes containing grain boundaries. In particular, the authors attempted to model solid electrolytes more realistically by performing extensive calculations ranging from the atomic to macroscopic levels, combining machine-learning potentials, ab initio molecular dynamics, and finite element simulations. After careful review of the manuscript, I think that the manuscript can be published in Nature Communications after addressing the following issues.

1. It is necessary to specify the meaning of the symbol 'sigma' used in the manuscript.

2. Simulating GBs is challenging because the detailed structure of GBs is not well known. How close are the GBs modeled by the authors to reality, especially in the thickness of GBs? The authors appear to have simulated GBs assuming intimate contact between grains. What happens to the conductivity if there are voids in the GBs, i.e. loose contact rather than close contact?

3. In general, GBs are known to cause a decrease in conductivity. However, the anion-ordered Li_6PS_5X (X = Cl, Br, and I) in Table 1 exhibits the opposite effect. There is a need for discussion as to whether this phenomenon is limited to these systems.

4. In Fig. 4a, the diffusivity of Li_6PS_5I exhibits non-Arrhenius behavior. Do Li_6PS_5Cl and Li_6PS_5Br also exhibit non-Arrhenius behavior? If not, why would the Arrhenius behavior differ depending on the type of halogen atom?

5. In Fig. 4c, the conductivities of disordered Li_6PS_5Cl are 1-2 orders of magnitude higher than the experimental values. However, previous ab initio calculations have shown much smaller differences from experiments [J. Mater. Chem. C, 2022, 10, 18294–18302; RSC Adv., 2022, 12, 31156–31166; J. Mater. Chem. A, 2024, 12, 993-1002]. Why is the discrepancy greater in this manuscript than in previous studies that did not consider the GB effect, even though the GB effect was taken into account?

Reviewer #2

(Remarks to the Author)
This study provides a quantitative understanding for ion transport in polycrystalline sulfide electrolytes by linking atomistic mechanisms at GBs to macroscopic transport through a constructed MD-to-FE workflow. The results are of potential interest to materials design. Nevertheless, several concerns require clarification before further consideration.
1.The validation of machine-learning potentials in GBs is limited to Σ3 and is assessed primarily through energy comparison. The authors are encouraged to extend the validation to additional GB types and include atomic force validation. Where feasible, comparison of key transport properties (e.g., diffusivities and activation energies) against AIMD results would further substantiate the MLMD-based results.
2.The continuum simulations adopt a fixed GB width (2.5 nm) across all compositions. Given that ion transport across the GB depends largely on the assumed GB thickness, the authors should assess how the GB thickness influences the ion transport.

3.The proposed linear correlation between Ea and anion radius is inferred from only three compositions in the anion-ordered phase, which appears not very convincing to establish a compelling trend. Additional corroboration is needed—for example, reporting activation energies for compositions spanning different degrees of anion disorder—to demonstrate the generality of this linear relationship.
4.Please add the corresponding analysis for Li_6PS_5Br in Fig. 3d and 3e (or, alternatively, in the Supporting Information).
5.The manuscript considers anion disorder only for Li_6PS_5Cl, while anions in Li_6PS_5Br and Li_6PS_5I are considered to be ordered. Table 1 shows that GB transport can differ significantly between ordered and disordered phases. In this context, comparing the experimentally measured activation energy of Li_6PS_5Br—typically associated with an anion-disordered phases—with a simulated GB activation energy derived from an anion-ordered model seems unreasonable.
6.Please expand the discussion on the implications for the rational design and processing of argyrodite electrolytes. This work captures grain-size effects arising from bulk and GB diffusion, but does not include other microstructural factors that often vary with grain size and can strongly affect practical conductivities, such as porosity and interparticle contact.

Version 1:

Reviewer comments:

Reviewer #1

(Remarks to the Author)
The revised manuscript is significantly improved compared to the previous one. It can be published as is.

Reviewer #2

(Remarks to the Author)
My concerns have been properly addressed.

Open Access This Peer Review File is licensed under a Creative Commons Attribution 4.0 International License, which permits use, sharing, adaptation, distribution and reproduction in any medium or format, as long as you give appropriate credit to the original author(s) and the source, provide a link to the Creative Commons license, and indicate if changes were made.
In cases where reviewers are anonymous, credit should be given to 'Anonymous Referee' and the source.
The images or other third party material in this Peer Review File are included in the article’s Creative Commons license, unless indicated otherwise in a credit line to the material. If material is not included in the article’s Creative Commons license and your intended use is not permitted by statutory regulation or exceeds the permitted use, you will need to obtain permission directly from the copyright holder.
To view a copy of this license, visit https://creativecommons.org/licenses/by/4.0/

Response to the Reviewers

Microstructural Insights into Fast Ion Transport in Solid Electrolytes via Multiscale Modeling

NCOMMS-26-002469-T

Yongliang Ou,[1,2,*] Lena Scholz,[3,†] Sanath Keshav,[3] Yuji Ikeda,[1] Marvin Kraft,[4,5] Sergiy Divinski,[6] Rafael Gómez-Bombarelli,[2] Wolfgang G. Zeier,[4,5] Felix Fritzen,[3,‡] and Blazej Grabowski[1,§]

[1] *Institute for Materials Science, University of Stuttgart, Stuttgart, Germany*
[2] *Department of Materials Science and Engineering, Massachusetts Institute of Technology, Cambridge, MA, USA*
[3] *Institute of Applied Mechanics, University of Stuttgart, Stuttgart, Germany*
[4] *Institute of Inorganic and Analytical Chemistry, University of Münster, Münster, Germany*
[5] *Institute of Energy Materials and Devices (IMD), IMD-4: Helmholtz-Institut Münster, Forschungszentrum Jülich, Münster, Germany*
[6] *Institute of Materials Physics, University of Münster, Münster, Germany*

(Dated: May 7, 2026)

Contents

Summary of the Response 1

Response to Reviewer #1 2

Response to Reviewer #2 11

References 21

Summary of the Response

We thank the reviewers for their careful evaluation of the manuscript and for their constructive comments. The manuscript has been significantly improved in response to these suggestions. The accuracy of machine learning potentials is further validated by comparison with ab initio and experimental data (R1Q5), as well as by additional benchmarking of energies and forces for a broader set of grain boundary configurations (R2Q1.1). The robustness of the multiscale modeling framework is reinforced through a sensitivity analysis of the grain boundary thickness (R2Q2). Moreover, the scope has been extended to include more ordered and disordered phases (R2Q3, R2Q4, R2Q5), non-Arrhenius behavior across multiple electrolyte systems (R1Q3, R1Q4), and the impact of porosity (R1Q2.2). These additions substantially improve the validity and generality of our conclusions.

Below you will find a point-by-point response in **black** to each comment in *gray*. For manuscript and supplementary information: Original text is in blue, additions in red, and deletions by ~~strikethrough~~.

* yongliang.ou@imw.uni-stuttgart.de
† scholz@mib.uni-stuttgart.de
‡ fritzen@mib.uni-stuttgart.de
§ blazej.grabowski@imw.uni-stuttgart.de

Response to Reviewer #1

Comments:
This study used multiscale modeling to gain microstructural insights into fast ion transport in argyrodite Li6PS5X (X = Cl, Br, and I) solid electrolytes containing grain boundaries. In particular, the authors attempted to model solid electrolytes more realistically by performing extensive calculations ranging from the atomic to macroscopic levels, combining machine-learning potentials, ab initio molecular dynamics, and finite element simulations. After careful review of the manuscript, I think that the manuscript can be published in Nature Communications after addressing the following issues.

We thank you for the positive assessment of our work and for confirming the value of the multiscale modeling approach. We have carefully addressed all comments raised and revised the manuscript accordingly. Detailed responses are provided below.

R1Q1. It is necessary to specify the meaning of the symbol 'sigma' used in the manuscript.

We fully agree with your suggestion. We have now clarified the meaning of the symbol Σ in both the Main and Methods sections.

`Line 131`, Section **Main**: (Originals in blue and additions in red)
We construct bicrystal models of anion-ordered Li_6PS_5Cl with single-type GBs to probe GB morphology. GBs are labeled by the coincidence-site lattice index Σ, defined during model construction, indicating structural complexity. Equilibrated GB structures, incorporating Li-ion disorder from diffusion and anion-sublattice distortions induced by GBs, are obtained using an MD–based annealing-and-quenching protocol [1] (see Methods).

`Line 569`, Section **Methods**:
The bicrystal GB models were constructed based on the ideal anion-ordered bulk of Li_6PS_5Cl and coincidence-site lattice theory [2] following our previous study [1]. The geometry of a GB structure can be labeled by the integer index Σ, which is defined as the ratio between the coincidence unit cell volume and the rotated unit cell volume [3].

R1Q2.1. Simulating GBs is challenging because the detailed structure of GBs is not well known. How close are the GBs modeled by the authors to reality, especially in the thickness of GBs?

We appreciate your recognition that simulating grain boundaries is inherently challenging. We have further clarified the physical basis of the grain boundary thickness and performed a sensitivity analysis, as detailed in our response to Reviewer#2 Q2.2. The corresponding results have been included in the Supplementary Information.
Briefly, the adopted grain boundary width is derived from atomistic structures equilibrated via molecular dynamics simulations at room temperature. The sensitivity analysis demonstrates that this choice yields diffusion activation energies consistent with experimental data, as well as polycrystalline diffusivities that are consistent across atomistic and continuum scales.

R1Q2.2. The authors appear to have simulated GBs assuming intimate contact between grains. What happens to the conductivity if there are voids in the GBs, i.e. loose contact rather than close contact?

We fully agree that voids and interparticle contacts can play an important role in determining macroscopic conductivity in practical solid-state electrolytes. In the present work, we focused on grain boundary effects under the assumption of intimate contact in order to isolate their intrinsic contribution. The reasons for not explicitly including void effects are as follows:

i. Void effects are well established in prior studies.

   The impact of porosity or tortuosity has been extensively investigated in both theoretical and experimental studies for solid-state electrolytes (e.g., Neumann *et al.* [4], Hamann *et al.* [5], Dixit *et al.* [6], and Hlushkou *et al.* [7]). These works consistently show that voids reduce the macroscopic conductivity. In contrast, grain boundary effects are more challenging to resolve due to their structural complexity and the need for multiscale methodologies [8]. Addressing this challenge is the primary focus of the present study.

ii. Void effects can be incorporated on top of our results.

As shown in Neumann *et al.* [4], the effects of grain boundaries and voids can be approximately decoupled at the continuum scale. Therefore, the transport properties of a porous polycrystalline electrolyte can be estimated by applying porosity-related corrections to the polycrystalline results obtained here. Figure 1 illustrates the expected reduction in macroscopic conductivity upon the introduction of voids. We have included this analysis in the Supplementary Information to extend the scope of the present study.

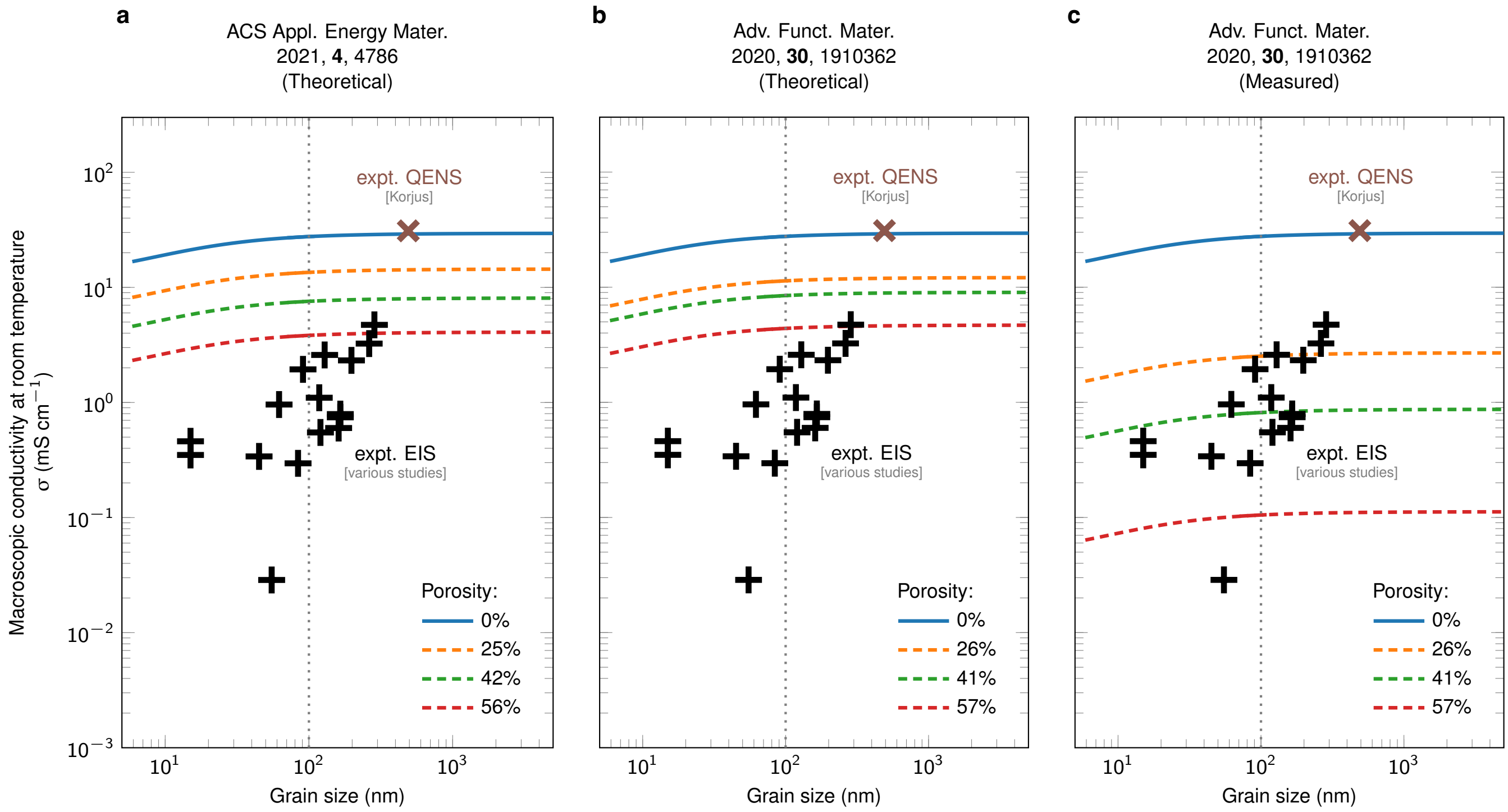

FIG. 1. **Impact of porosity on macroscopic conductivity of solid electrolytes at 300 K.** Li_6PS_5Cl with 50% anion disorder is considered. The grain-size effect is estimated for a close-contact polycrystal using our multiscale modeling framework, while the porosity effect is applied as a shift on top of these close-contact results, based on theoretical models (ACS Appl. Energy Mater. [4] and Adv. Funct. Mater. [5]) and experimental measurements [5]. Accounting for porosity reduces the macroscopic conductivity by one to two orders of magnitude, bringing predictions for anion-disordered Li_6PS_5Cl closer to experimental observations.

iii. Extension of our multiscale framework is straightforward.

Our multiscale modeling framework can be naturally extended to explicitly include additional microstructural features such as voids. For example, surface diffusivities obtained from atomistic simulations could be incorporated into continuum models to describe transport along pore surfaces. We have added a brief indication of such extensions to highlight the broader applicability of our multiscale approach.

`Line 320`, Section **Results**:

The predicted values are roughly an order of magnitude higher than the highest experimental values measured by electrochemical impedance spectroscopy. In contrast, the predicted values are roughly an order of magnitude higher than the highest experimental values measured by electrochemical impedance spectroscopy, which probes macroscopic transport. This discrepancy is partially mitigated by considering voids in solid electrolytes (Supplementary Fig. 1). Additional discrepancy may arise from differences in density, pellet processing, or electrode–sample contact [9].

`Line 504`, Section **Methods**:

The proposed multiscale modeling framework enables prediction of macroscopic diffusivities in polycrystalline solid electrolytes up to device-relevant scales (Supplementary Fig. S4d). Its main strength lies in balancing accuracy and efficiency across the entire modeling process, through an optimized active learning scheme for machine-learning potentials, a data-efficient multiscale workflow, and tailored methods for resolving GBs in FE simulations. Moreover, our modeling framework can be readily extended to explicitly include additional microstructural features. For example, surface diffusivities obtained from atomistic simulations can be incorporated into continuum models to describe transport along pore surfaces, enabling multiscale modeling of porous polycrystalline solid electrolytes.

R1Q3. In general, GBs are known to cause a decrease in conductivity. However, the anion-ordered Li6PS5X (X = Cl, Br, and I) in Table 1 exhibits the opposite effect. There is a need for discussion as to whether this phenomenon is limited to these systems.

We agree that grain boundaries generally reduce ionic conductivity in many solid electrolytes, as shown experimentally for garnet-type $Li_7La_3Zr_2O_{12}$ [10], $Li_{6.4}La_3Zr_{1.4}Ta_{0.6}O_{12}$ [11], and perovskite $Li_{0.33}La_{0.56}TiO_3$ [12].

However, the influence of grain boundaries in sulfide electrolytes is less well understood due to experimental challenges. Characterizing grain boundary morphology and isolating its contribution to conduction remain challenging in sulfides owing to their intrinsically low GB resistance, even at low temperatures, and beam-induced decomposition during high-resolution transmission electron microscopy. At the same time, experimental control over anion ordering remains difficult, leaving a significant gap in the understanding of structure–property relationships [13].

Computational studies have historically been limited by the availability of accurate interatomic potentials, restricting prior investigations to relatively simple grain boundaries embedded in small simulation cells, for example, $\Sigma5$ in Li_6PS_5Cl [14] and up to $\Sigma25$ in β-Li_3PS_4 [15].

In this work, we developed accurate machine learning potentials capable of modeling complex grain boundaries (up to $\Sigma99$) in argyrodite Li_6PS_5X ($X \in \mathrm{Cl, Br, I}$), and found that grain boundaries can enhance Li diffusion in the anion-ordered phase. This finding is consistent with previous *ab initio* studies on argyrodites, e.g., Li_6PS_5Br [16], and aligns with experimental observations of enhanced conductivity in nanocrystalline Li_6PS_5I, attributed to the combined effect of higher grain boundary density and increased disordered-phase volume fraction [17].

To contextualize these findings, we summarize selected literature reporting cases where grain boundaries may enhance ion transport:

i. Sulfides.

Grain boundaries enhance Na-ion diffusion in Na_3PS_4 [18], and in β-Li_3PS_4, diffusion of all the investigated grain boundaries exceeds that of the crystalline bulk [15].

ii. Oxides.

The $\Sigma3$ GB in $Li_7La_3Zr_2O_{12}$ reduces Li-ion diffusion barrier by about 50 meV [19], and certain GBs in $LiZr_2(PO_4)_3$ show higher conductivity than the bulk [20].

iii. Halides.

A $\Sigma3$ GB in Li_3InCl_6 reduce diffusion barriers by about 30 meV [21].

These results suggest that the enhancement of ionic transport by GBs may not unique to argyrodite sulfides, but can occur in other solid electrolytes depending on structural and chemical context. We have added additional information in the revised manuscript to provide broader context for the reader.

`Line 370`, Section **Discussion and Conclusion**:

In contrast, in systems with intrinsically low bulk diffusivity, GBs facilitate Li^+ transport, as exemplified by anion-ordered Li_6PS_5I. Similar GB-induced diffusion enhancement has also been predicted in sulfides such as Na_3PS_4 [18] and β-Li_3PS_4 [15], in the oxide $LiZr_2(PO_4)_3$ [20], and in the halide Li_3InCl_6 [21]. Consequently, nanosizing can offer a strategy to improve potential solid electrolytes lacking intrinsic superionic conductivity.

R1Q4. In Fig. 4a, the diffusivity of Li6PS5I exhibits non-Arrhenius behavior. Do Li6PS5Cl and Li6PS5Br also exhibit non-Arrhenius behavior? If not, why would the Arrhenius behavior differ depending on the type of halogen atom?

It is true that the non-Arrhenius diffusion behavior was mainly discussed for the anion-ordered Li_6PS_5I system. The initial motivation was to clarify the mechanism underlying the experimentally observed non-Arrhenius behavior for Li_6PS_5I [17]. Using our multiscale simulation framework, we show that this behavior can arise from grain boundaries in polycrystalline samples. In particular, it is attributed to the contrast between bulk and grain-boundary diffusivities, which exhibit different temperature dependencies. We provide an analysis of non-Arrhenius behavior predicted for various argyrodite systems below.

i. Anion-ordered argyrodites.

The non-Arrhenius behavior is observed for the anion-ordered Li_6PS_5I and Li_6PS_5Br, as shown in Fig. 2. At higher temperatures, the diffusivity ratios between grain boundary and bulk are relatively small, and the total diffusivity is dominated by the bulk diffusivity due to the large volume fraction of bulk in the polycrystal model. At lower temperatures, the diffusivity ratios between grain boundary and bulk become larger, and the effect of grain boundary become more critical. Therefore, a grain-boundary dominated regime is resulted. Such grain boundary-caused non-Arrhenius behavior was reported in a previous study [8].

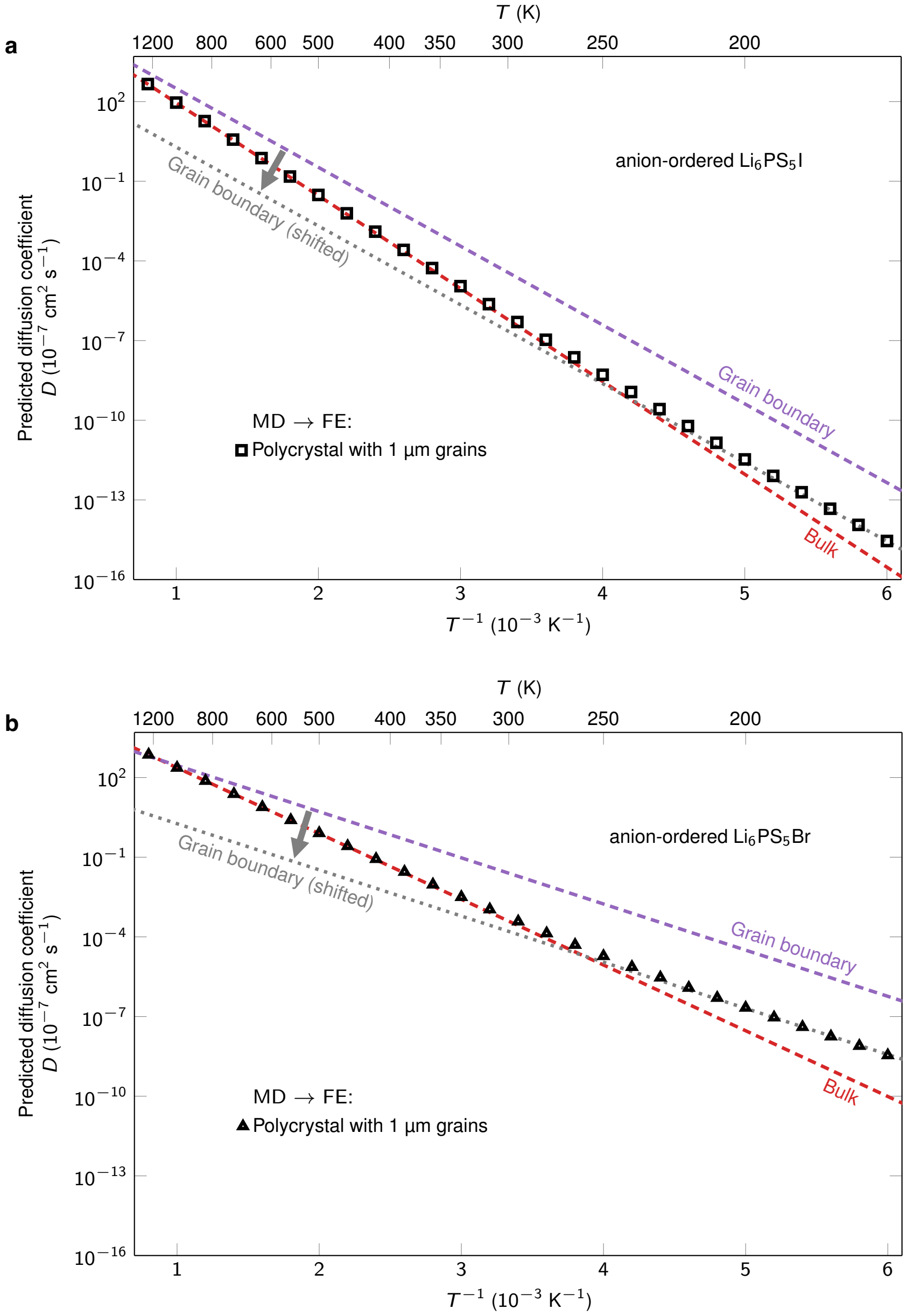

FIG. 2. **Non-Arrhenius diffusion behavior arising from grain boundaries in anion-ordered argyrodites.** Temperature-dependent diffusivities of, **a**, anion-ordered Li_6PS_5I and, **b**, anion-ordered Li_6PS_5Br. Polycrystalline models with 1 µm-sized grains are used for demonstration. Dashed lines are obtained from Arrhenius fits to bulk and grain-boundary diffusivities. Additional dotted lines, shifted from the grain-boundary Arrhenius fits, highlight the grain-boundary-dominated regime at low temperatures. The non-Arrhenius behavior depends on the ratio of bulk to grain-boundary diffusivities and its temperature dependence.

ii. Anion-disordered argyrodites.

The non-Arrhenius behavior *cannot* be clearly observed for anion-disordered argyrodites in the investigated temperature range, as exemplified for Li_6PS_5Cl with 50% anion disorder in Fig. 3. The bulk and grain boundary diffusivities are similar, even at the low temperature range, so the polycrystal diffusion behavior closely follow the bulk Arrhenius fit, without showing non-Arrhenius behavior in the investigated temperature range.

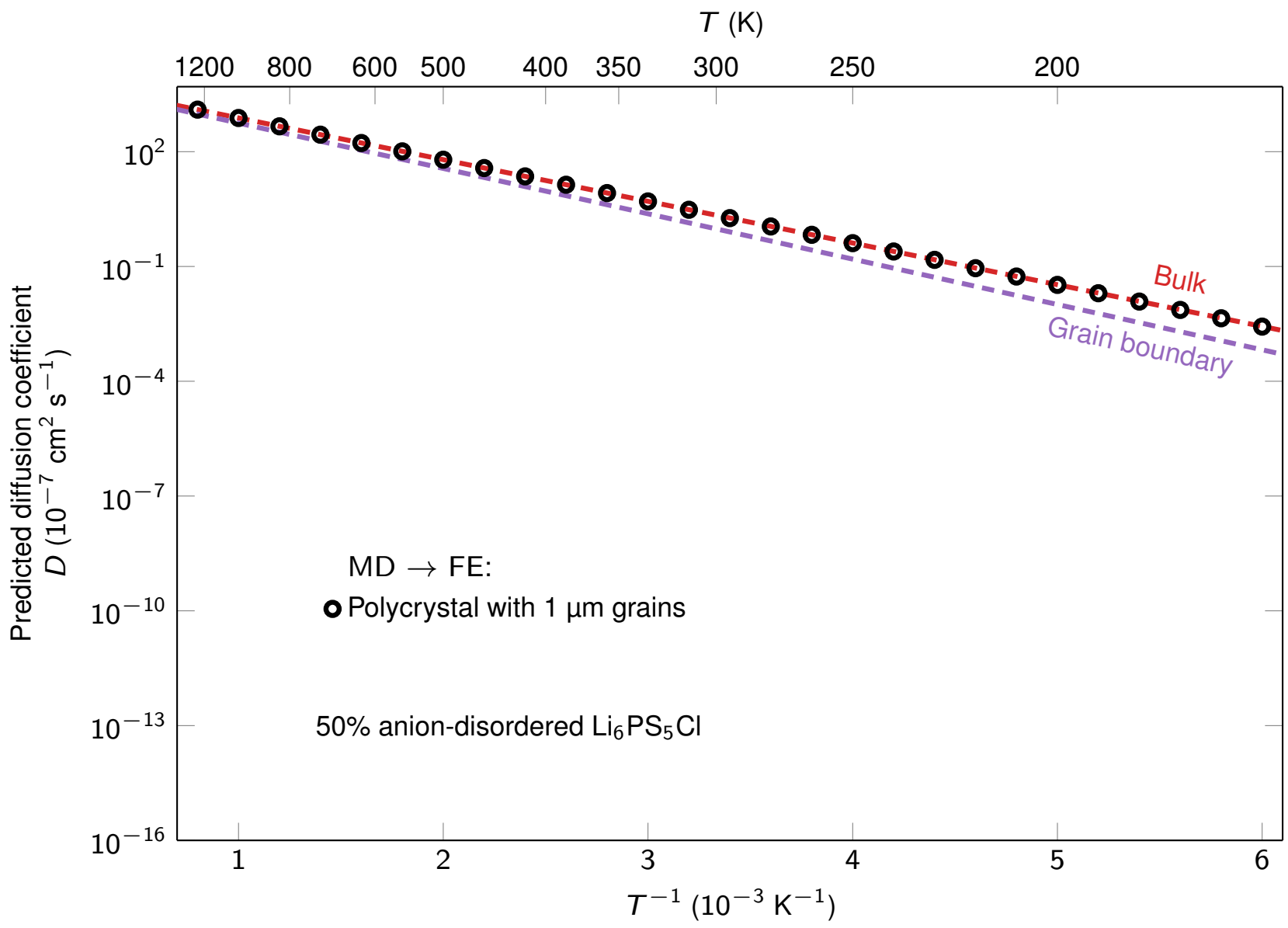

FIG. 3. **Diffusion behavior of polycrystalline anion-disordered argyrodites.** Results from 50% anion-disordered Li_6PS_5Cl polycrystalline model are exemplified. Dashed lines are obtained from Arrhenius fits to bulk and grain-boundary diffusivities. Because bulk and grain-boundary diffusivities are comparable, the influence of grain boundaries is minimal, and the polycrystalline diffusivity closely follows the bulk Arrhenius relation. Non-Arrhenius behavior is not clearly observed within the investigated temperature range.

iii. Comparison to experimental observations.

Figure 4 compares predicted temperature-dependent diffusivities of different argyrodite polycrystals. Note that the non-Arrhenius behavior, including the transition temperature, is also dependent on the grain size of the polycrystal model. Our results are consistent with experimental observations, which report non-Arrhenius diffusion behavior in Li_6PS_5I but not in Li_6PS_5Br [17]. In experiments, Li_6PS_5I is predominantly anion-ordered, whereas Li_6PS_5Br is generally anion-disordered. Further application of our multiscale modeling framework enables *a priori* screening of material systems that shows anomalous diffusion behavior.

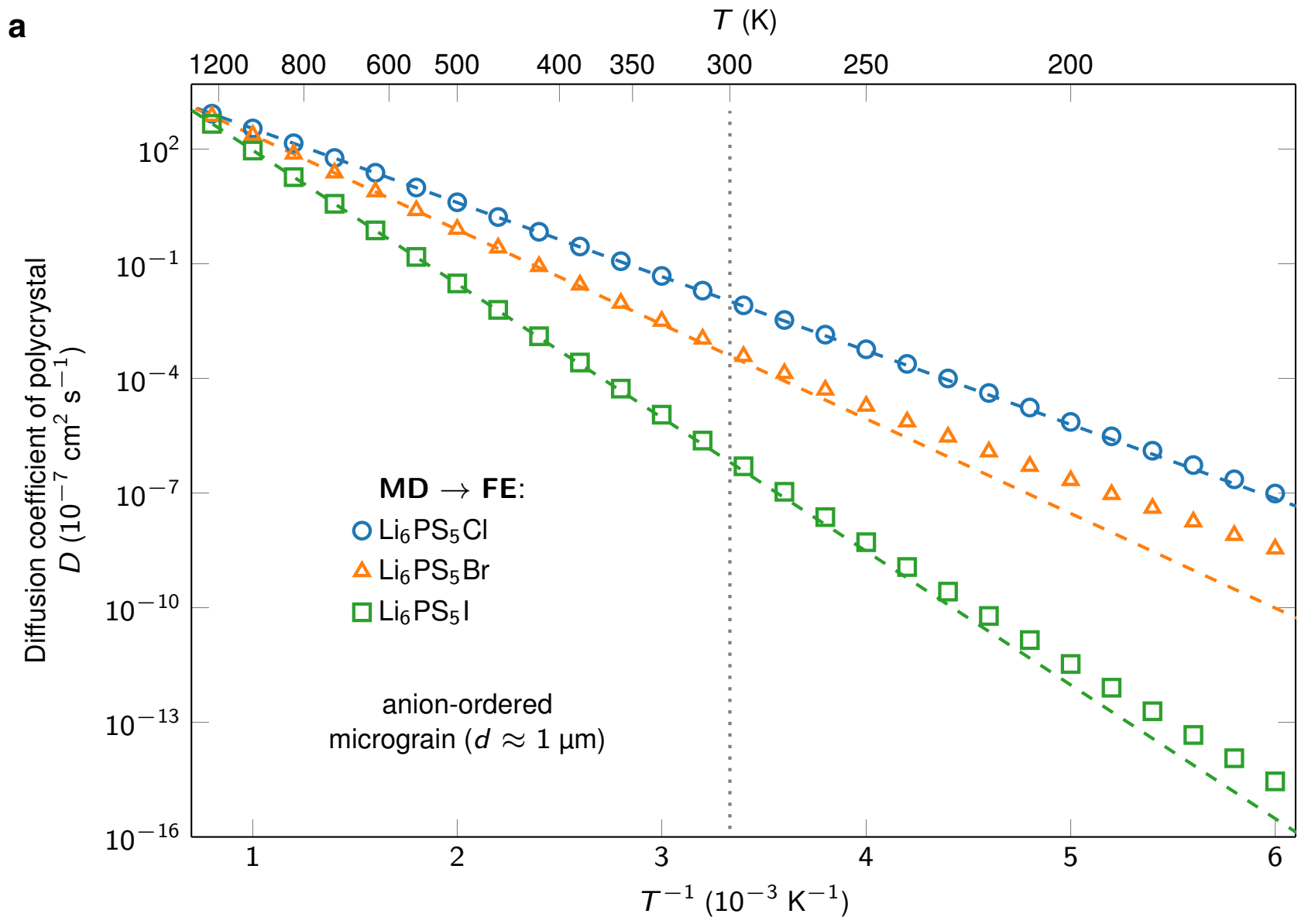

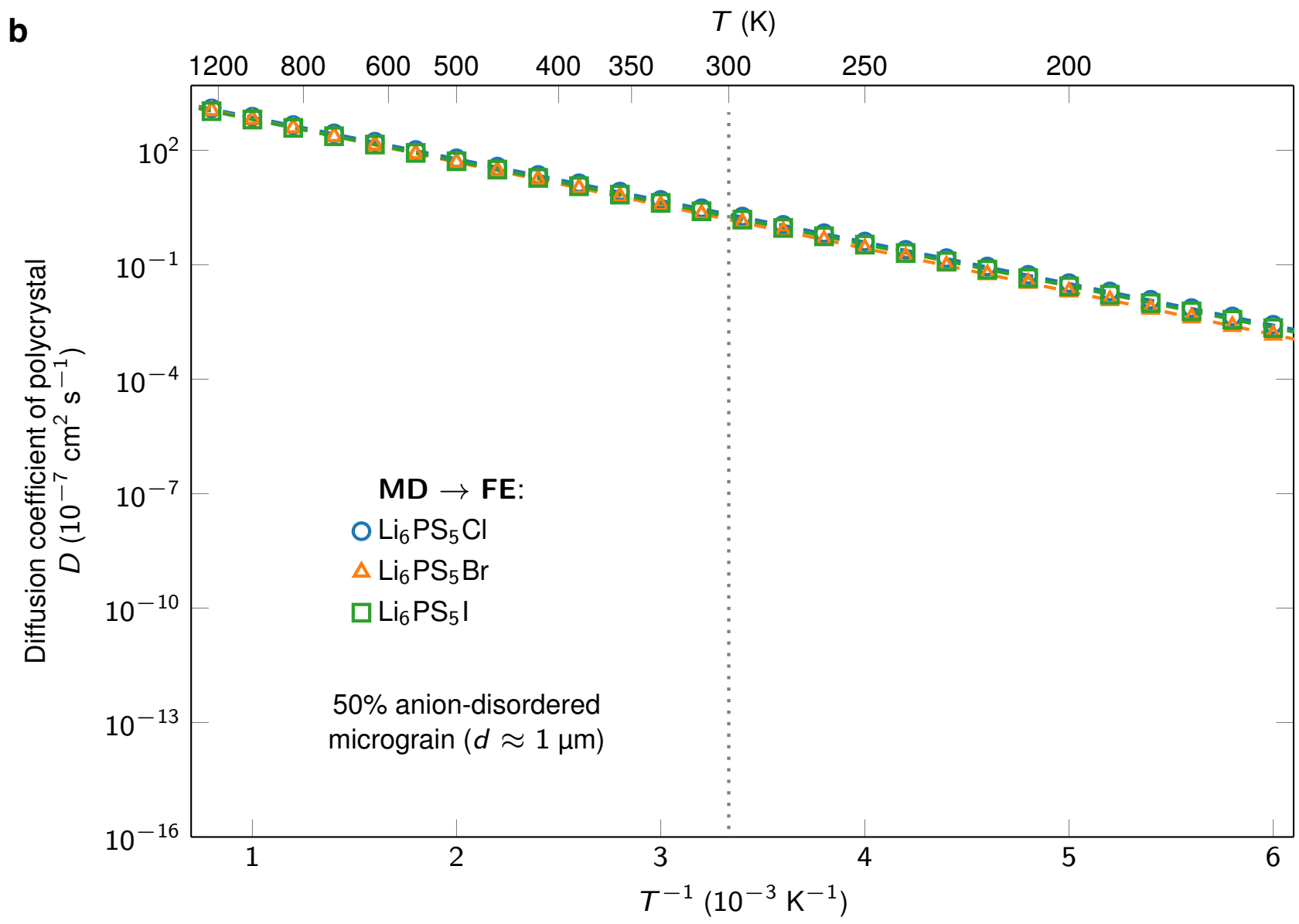

FIG. 4. **Comparison of predicted temperature-dependent diffusivities of polycrystalline Li_6PS_5X with $X \in$ {Cl, Br, I}.** **a**, Without anion disorder. The non-Arrhenius diffusion behavior is observed for Li_6PS_5Br and Li_6PS_5I, but cannot be clearly observed for Li_6PS_5Cl in the presented temperature range. **b**, With anion disorder. The non-Arrhenius diffusion behavior cannot be clearly observed for 50% disordered Li_6PS_5Cl and Li_6PS_5Br in the presented temperature range.

An additional note is added in the Supplementary Information to clarify the system-dependent non-Arrhenius behavior.

`Line 403`, Section **Discussion and Conclusion**:

The simulations show that GBs can give rise to non-Arrhenius behavior in Li_6PS_5I, thereby providing a possible atomic-scale mechanism for the experimental observations [22]. Our results for Li_6PS_5X reveal that both the transition temperature and the magnitude of non-Arrhenius behavior are halogen-dependent (Supplementary Note 5), consistent with the softened and ultimately absent anomaly observed for $Li_6PS_5Br_{0.5}I_{0.5}$ and Li_6PS_5Br, respectively [22]. In halide-rich argyrodites, GBs hinder Li^+ transport, leading to a predicted increase in the low-temperature diffusion barrier, consistent with experiments [23].

`Line 207`, Section **Supplementary Note 5**:

The accuracy and efficiency of the quality-level-based local active learning scheme are demonstrated in Figs. S5 and S6 for a Σ3 GB. Figure 7 shows the training energy and force errors obtained independently for various GB types ranging from Σ3 up to Σ99. Although large grain boundaries such as Σ9 and Σ11 are structurally more complex than Σ3 grain boundaries, only small increases are shown for training errors in energy (within 2 meV atom^{-1}) and

force (within $0.2\,\mathrm{eV\,Å^{-1}}$). These results indicate that GB types exert only a limited effect on the training structures obtained via local active learning.

We also evaluated the accuracy of the moment tensor potentials for a $\Sigma 9$ GB and provide a comparison of the results to a $\Sigma 3$ GB in Fig. 8. Moment tensor potentials reproduce the density-functional-theory-derived grain-boundary formation energies for both GB types. Additional density-functional-theory results also confirm that complex grain boundaries such as $\Sigma 9$ generally have higher formation energy than a $\Sigma 3$ GB. Next, we performed ab initio molecular dynamics simulations for the $\Sigma 3$ and $\Sigma 9$ grain-boundary structures at 600 K, to generate configurations for validating forces predicted by the moment tensor potentials. Results show that errors for both grain boundaries are less than $0.2\,\mathrm{eV\,Å^{-1}}$, with the errors for $\Sigma 9$ only about $0.03\,\mathrm{eV\,Å^{-1}}$ higher, confirming the high accuracy of the developed moment tensor potentials.

R1Q5. In Fig. 4c, the conductivities of disordered Li6PS5Cl are 1-2 orders of magnitude higher than the experimental values. However, previous ab initio calculations have shown much smaller differences from experiments [J. Mater. Chem. C, 2022, 10, 18294–18302; RSC Adv., 2022, 12, 31156–31166; J. Mater. Chem. A, 2024, 12, 993-1002]. Why is the discrepancy greater in this manuscript than in previous studies that did not consider the GB effect, even though the GB effect was taken into account?

We appreciate your careful comparison of our results with the literature and for providing these three important references. A comparison with *ab initio* results and experimental values is presented below.

i. Comparison to previous *ab initio* studies.

Figure 5 shows a comparison using the representative system Li_6PS_5Cl with 50% anion disorder. We performed independent molecular dynamics simulations for six simulation models with random arrangement of Cl/S atoms. Different arrangements of Cl/S atoms were also considered for the RSC Adv. study [24].

Diffusion barriers predicted from our work show much smaller variations due to different Cl/S arrangements (Fig. 5a), likely due to the three orders of magnitude more atoms in the simulation cell and an order of magnitude longer simulation time compared to prior studies (Fig. 5c). Nevertheless, the predicted diffusion barrier of 213 meV from our study is within the range resulted from previous *ab initio* studies (202 to 282 meV) [24–26].

In all studies, diffusion coefficients at 300 K were derived by extrapolation from high temperature diffusivities based on the Arrhenius relations (Fig. 5b). Such extrapolation is sensitive to statistical errors of the Arrhenius fitting and accessibility of low temperature data points. The predicted diffusion coefficients are higher than previously reported values but remain within one order of magnitude. Discrepancies may arise from the limited length and time scales accessible in earlier *ab initio* studies.

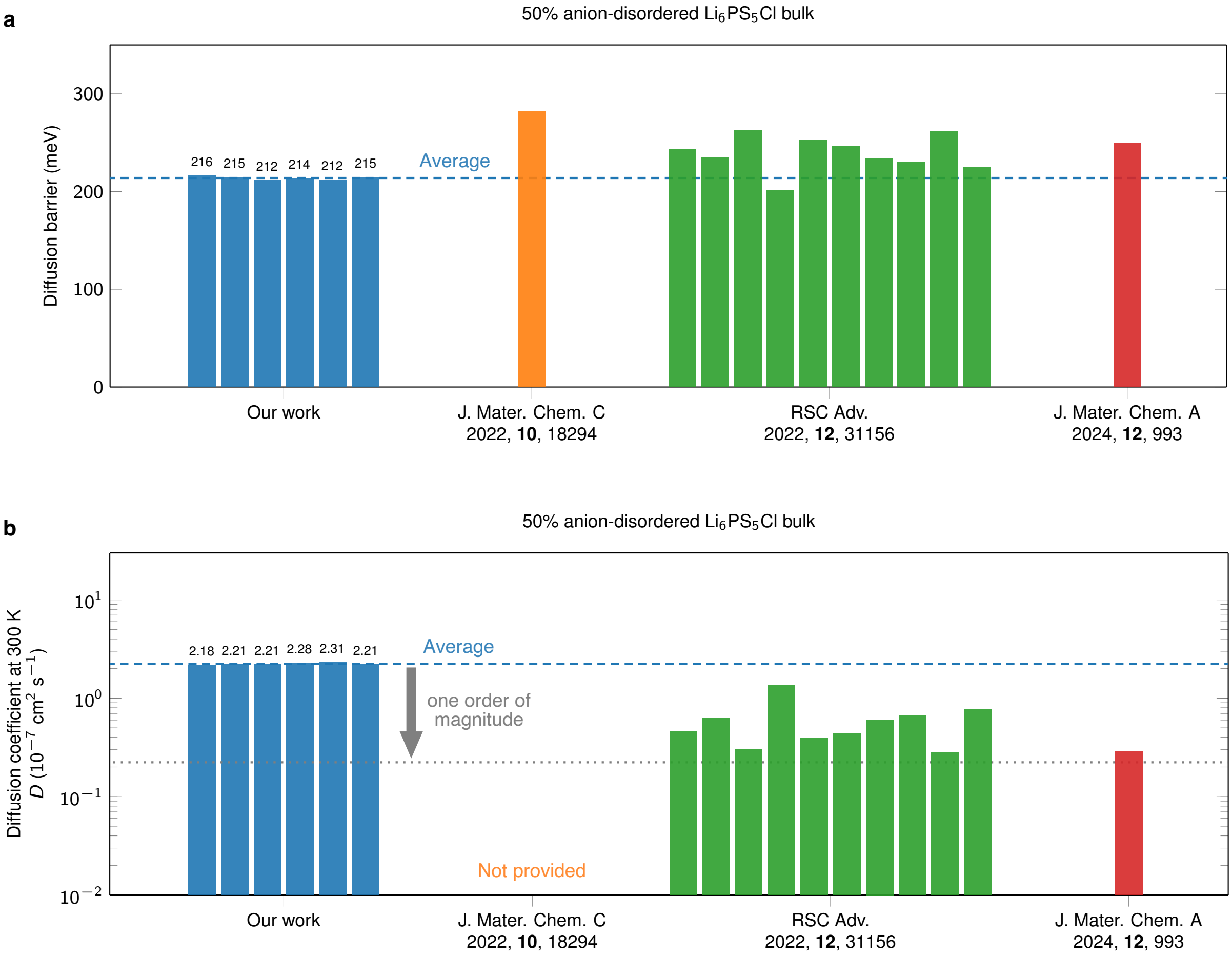

c

	Our work	J. Mater. Chem. C 2022, **10**, 18294	RSC Adv. 2022, **12**, 31156	J. Mater. Chem. A 2024, **12**, 993
Simulation method	*ab initio* → MLP	*ab initio*	*ab initio*	*ab initio*
Simulation model (atoms)	52 000	52	52	52
Simulation time (ps)	5 000	120	150	300
Lowest simulation temperature (K)	350	500	600	700

FIG. 5. **Comparison of MLP–MD-predicted diffusion properties with *ab initio* results.** In all studies, molecular dynamics simulations are performed and diffusion properties are extracted from the mean-squared displacement of Li ions. Bulk Li_6PS_5Cl with 50% anion disorder is considered. **a**, Comparison of diffusion barriers. **b**, Comparison of diffusion coefficients extrapolated to 300 K. Dashed lines indicate averaged values from our work and facilitate comparison with previous studies (J. Mater. Chem. C [25], RSC Adv. [24], J. Mater. Chem. A [26]). A dotted line indicates a value one order of magnitude lower than our predicted diffusivities for reference. **c**, Comparison of simulation length and time scales, together with the lowest temperatures sampled. Larger simulation models are essential to capture realistic anion disorder, whereas longer simulation times are required to obtain statistically converged diffusivities based on the Einstein relation. Additionally, access to lower temperatures is critical for reliable Arrhenius extrapolation to obtain room-temperature diffusivities.

ii. Comparison to experimental values.

We validate the MLP–MD-predicted diffusivities by comparison with experimental values for anion-disordered Li_6PS_5Cl (Fig. 6). Our results show good agreement with experiments, confirming the high accuracy of the machine-learning potentials developed in the present study.

iii. Discrepancy likely arise from factors other than grain boundaries.

Even when accounting for grain boundary effects, a discrepancy persists between predictions from our multiscale modeling framework and experimental values from electrochemical impedance spectroscopy measurements. Our investigations indicate that additional factors, including interparticle voids (see our previous response Q2.2) and electrode–sample contact, likely contribute to this discrepancy.

An addition note is added in the Supplementary Information to comparisons with previous *ab initio* studies.

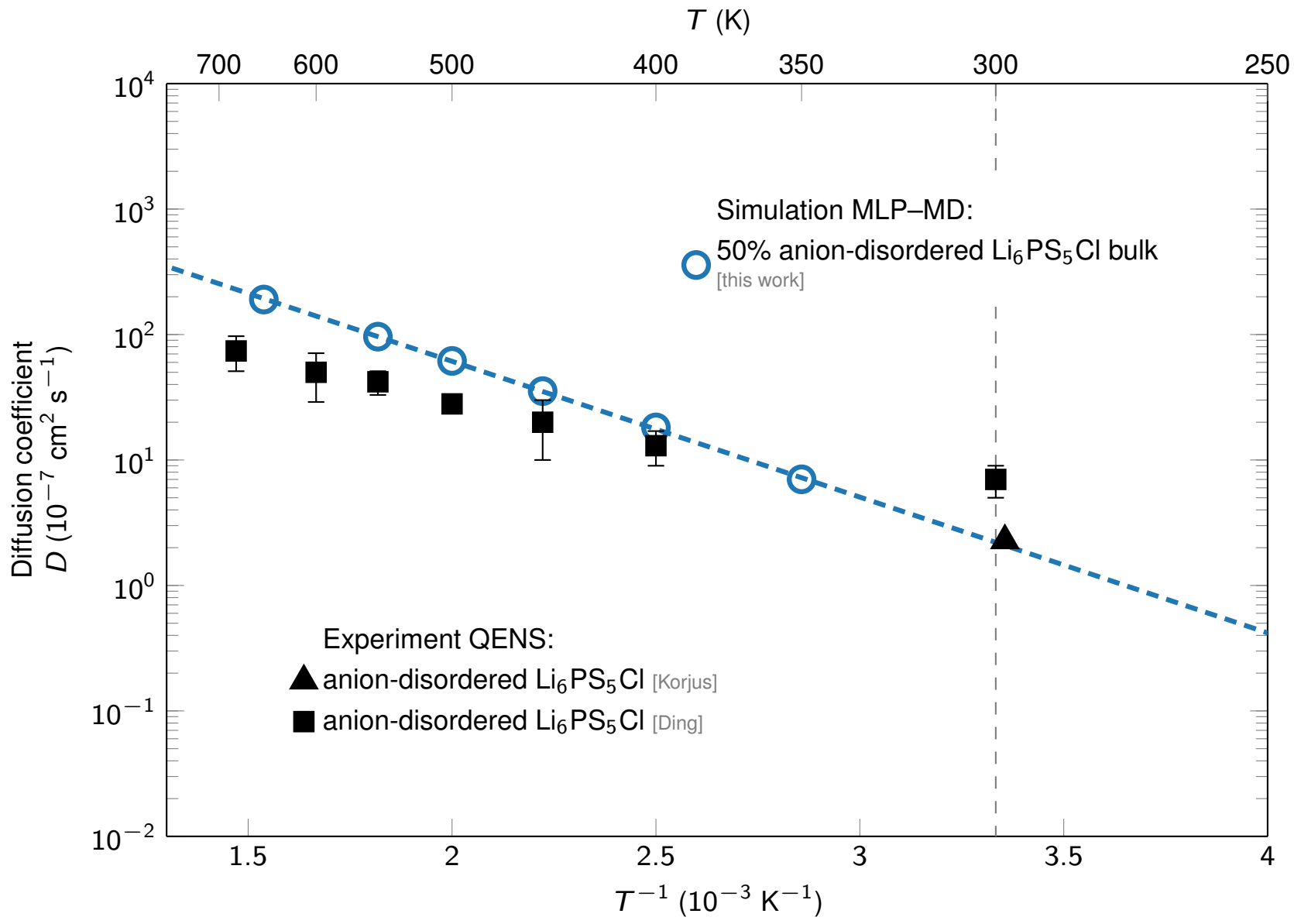

FIG. 6. **Validation of MLP–MD-predicted diffusivities against experiments for intrinsic bulk.** Diffusivities of bulk Li_6PS_5Cl with 50% anion disorder were obtained from molecular dynamics (MD) simulations accelerated by machine-learning potentials (MLPs). The dashed line represents an Arrhenius fit to the MLP–MD diffusivities. Our predictions agree well with recent quasielastic neutron scattering (QENS) measurements (Korjus *et al.* [27], Ding *et al.* [28]), which probe short-time, local Li motion.

`Line 92`, Section **Supplementary Note 2**:

Figure 5 shows a comparison of results from moment tensor potentials and previous ab initio studies using the representative system Li_6PS_5Cl with 50% anion disorder. We performed independent molecular dynamics simulations for six simulation models with random arrangement of Cl/S atoms. Different arrangements of Cl/S atoms was also considered for the RSC Adv. study [24]. Diffusion barriers predicted from our work show much smaller variations due to different Cl/S arrangements (Fig. 5a), likely due to the three orders of magnitude more atoms in the simulation cell and an order of magnitude longer simulation time compared to prior studies (Fig. 5c). Nevertheless, the predicted diffusion barrier of 213 meV from our study is within the range resulted from previous ab initio studies (202 to 282 meV) [24–26]. In all studies, diffusion coefficients at 300 K were derived by extrapolation from high temperature diffusivities based on the Arrhenius relations (Fig. 5b). Such extrapolation is sensitive to statistical error of the Arrhenius fitting and accessibility of low temperature data points. The predicted diffusion coefficients are higher than previously reported values but remain within one order of magnitude. Discrepancies may arise from the limited length and time scales accessible in earlier ab initio studies. We further validate the MTP-MD–predicted diffusivities by comparison with experimental values for anion-disordered Li_6PS_5Cl (Fig. S10). Our results show good agreement with quasielastic neutron scattering measurements.

The transferability of moment tensor potentials developed via local active learning was evaluated across the anion ordering chemical space in argyrodites (Table S1). Transferability is relatively good for Li_6PS_5Cl, whereas it is limited for Li_6PS_5Br and Li_6PS_5I.

Response to Reviewer #2

Comments:
This study provides a quantitative understanding for ion transport in polycrystalline sulfide electrolytes by linking atomistic mechanisms at GBs to macroscopic transport through a constructed MD-to-FE workflow. The results are of potential interest to materials design. Nevertheless, several concerns require clarification before further consideration.

Thank you for your constructive assessment of our work and for recognizing the importance of quantitative studies of ion transport in polycrystalline materials. We have carefully addressed all concerns raised and revised the manuscript accordingly. Detailed responses are provided below.

R2Q1.1. The validation of machine-learning potentials in GBs is limited to Σ3 and is assessed primarily through energy comparison. The authors are encouraged to extend the validation to additional GB types and include atomic force validation.

We fully agree with your suggestion that additional validation of the machine-learning potentials for more complex grain boundaries are needed. We provide additional details on the development of the machine-learning potentials and further validations below.

i. Errors for training structures.

Figure 7 shows the training energy and force errors of the machine learning potentials developed via local active learning independently for various grain boundary types ranging from Σ3 up to Σ99. Although large grain boundaries such as Σ9 and Σ11 are structurally more complex than Σ3 grain boundaries, only small increases are shown for training errors in energy (within $2\,\mathrm{meV\,atom^{-1}}$) and force (within $0.2\,\mathrm{eV\,Å^{-1}}$). These results indicate that grain boundary types exert only a limited effect for the training structures obtain via local active learning.

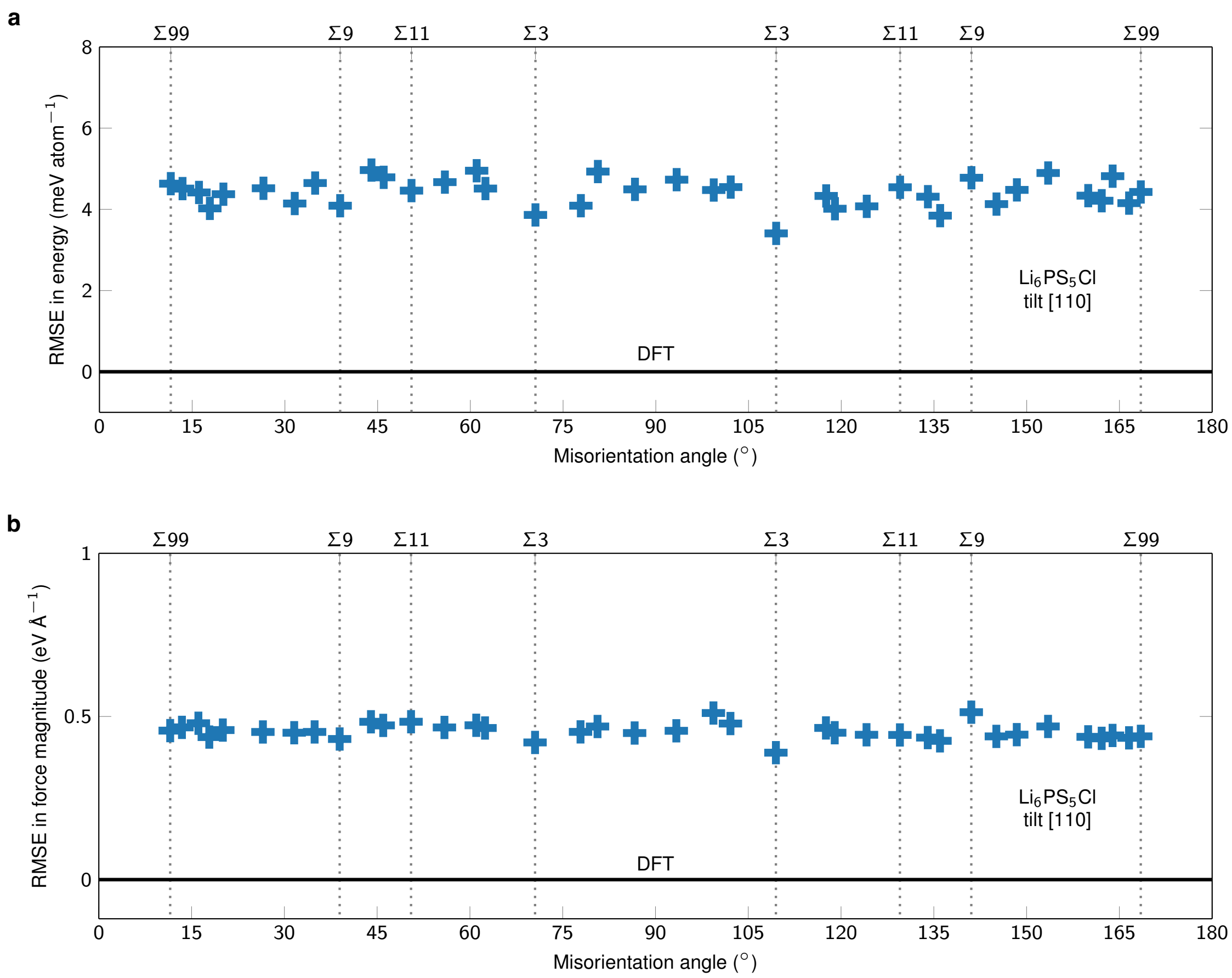

FIG. 7. **Energy and force errors for training structures obtained through local active learning.** **a**, Root-mean-square errors (RMSEs) in machine-learning-potential-predicted energies are around $4\,\mathrm{meV\,atom^{-1}}$. **b**, RMSEs in force are around $0.5\,\mathrm{eV\,Å^{-1}}$. Errors in energies and forces remain consistent across different grain-boundary types. Results are based on single-type tilt grain boundaries in anion-ordered Li_6PS_5Cl with a [110] rotation axis.

ii. Errors for validation structures.

Following your suggestion, we additionally evaluated the accuracy of the machine-learning potentials for a $\Sigma 9$ grain boundary and provide a comparison of the results to a $\Sigma 3$ grain boundary in Fig. 8.

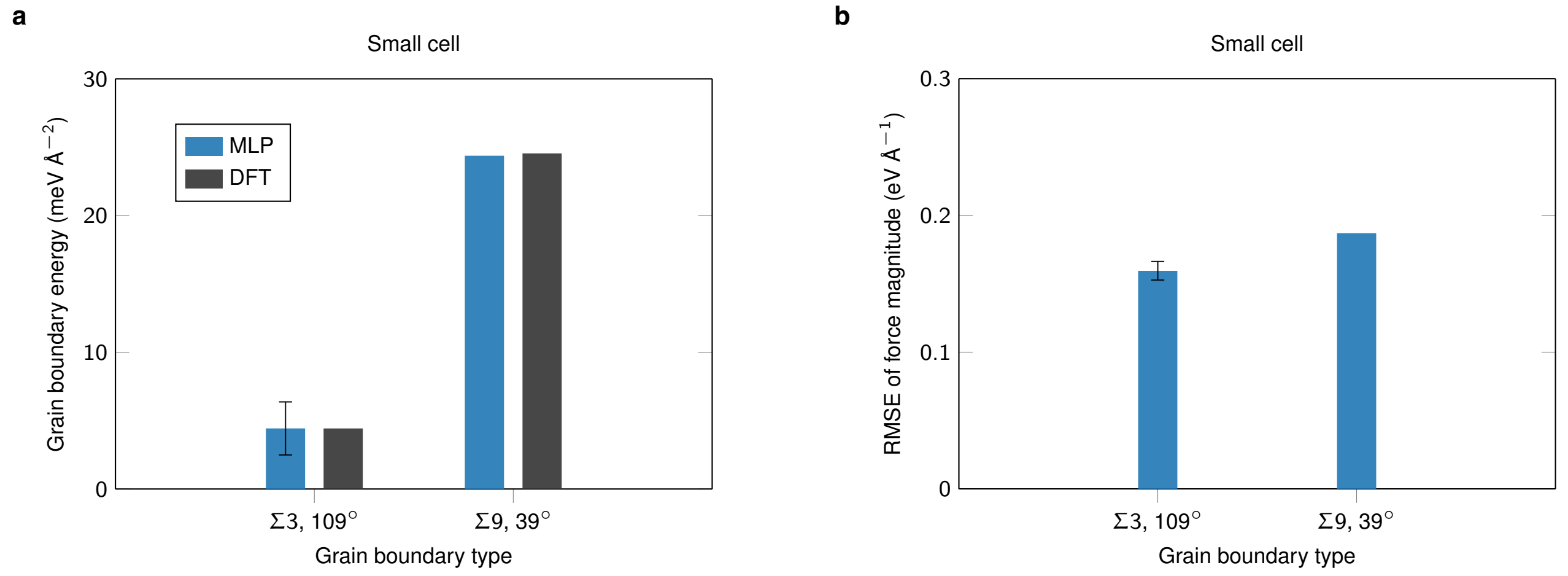

FIG. 8. **Validation of machine-learning potentials (MLPs) for different types of grain boundaries.** Results for representative $\Sigma 3$ and $\Sigma 9$ grain boundaries in Li_6PS_5Cl, with misorientation angles of about 109° and 39°, respectively, are compared. **a**, Grain-boundary energetics at 0 K. MLPs reproduce grain-boundary formation energies obtained from density-functional theory (DFT). $\Sigma 9$ grain boundaries exhibit formation energies about 20 meV Å^{-2} higher than $\Sigma 3$. Error bars represent standard deviation of results from five independently trained potentials. **b**, Force prediction errors at 600 K. *Ab initio* molecular dynamics simulations of grain-boundary structures at 600 K were used to generate validation configurations. Force errors for both $\Sigma 3$ and $\Sigma 9$ grain boundaries remain below 0.2 eV Å^{-1}. Due to the high computational cost of DFT, grain-boundary structures were modeled in small simulation cells. Error bars represent the standard deviation across five independently trained MLPs obtained via local active learning.

Machine learning potentials reproduce the density-functional-theory-derived grain-boundary formation energies for both grain boundary types. Additional density-functional-theory results also confirm that complex grain boundaries such as $\Sigma 9$ generally have higher formation energy compared to $\Sigma 3$ grain boundary.

Next, we performed *ab initio* molecular dynamics simulations for the $\Sigma 3$ and $\Sigma 9$ grain-boundary structures at 600 K, to generate configurations for validating forces predicted by the machine-learning potentials. Results show that errors for both grain boundaries are less than 0.2 eV Å^{-1}, with the errors for $\Sigma 9$ only about 0.03 eV Å^{-1} higher, confirmed the high accuracy of the developed machine-learning potentials. We have incorporated these new results in the revised manuscript and supplementary information.

`Line 74`, Section **Results**:

Fig. 1. **GB impacts in anion-ordered Li_6PS_5Cl.** **a**, Predicted GB formation energy at 0 K. GB energies of $\Sigma 3$ and $\Sigma 9$ GBs in small simulation cells were calculated by density-functional theory (DFT) based on the structures relaxed by machine-learning potentials, which validates the accuracy of the machine-learning potentials. **b**, Predicted GB self-diffusivities D^{GB} at 600 K. The $D_{\parallel}^{GB}$ and $D_{\perp}^{GB}$ represent the diffusion within and across the GB planes, respectively.

`Line 79`, Section **Supplementary Note 2**:

Figure 7 shows the training energy and force errors obtained independently for various GB types ranging from $\Sigma 3$ up to $\Sigma 99$. Although large grain boundaries such as $\Sigma 9$ and $\Sigma 11$ are structurally more complex than $\Sigma 3$ grain boundaries, only small increases are shown for training errors in energy (within 2 meV atom^{-1}) and force (within 0.2 eV Å^{-1}). These results indicate that GB types exert only a limited effect on the training structures obtained via local active learning.

We also evaluated the accuracy of the moment tensor potentials for a $\Sigma 9$ GB and provide a comparison of the results to a $\Sigma 3$ GB in Fig. 8. Moment tensor potentials reproduce the density-functional-theory-derived grain-boundary formation energies for both GB types. Additional density-functional-theory results also confirm that complex grain boundaries such as $\Sigma 9$ generally have higher formation energy than a $\Sigma 3$ GB. Next, we performed ab initio molecular dynamics simulations for the $\Sigma 3$ and $\Sigma 9$ grain-boundary structures at 600 K, to generate configurations for validating forces predicted by the moment tensor potentials. Results show that errors for both grain boundaries are less than 0.2 eV Å^{-1}, with the errors for $\Sigma 9$ only about 0.03 eV Å^{-1} higher, confirming the high accuracy of the developed moment tensor potentials.

R2Q1.2. Where feasible, comparison of key transport properties (e.g., diffusivities and activation energies) against AIMD results would further substantiate the MLMD-based results.

Thank you for your suggestion. We have additionally compared key transport properties, including both room-temperature diffusivities and activation energies from machine-learning potentials to *ab initio* MD results and available experimental values, as discussed in our response to Reviewer#1 Q5. Corresponding details have been added to the Supplementary Information.

Briefly, transport properties derived from machine-learning potentials are consistent with previous *ab initio* studies while exhibit much better statistics due to exponentially increased length and time simulation scales. Our predictions agree well with experimental values from quasi-elastic neutron scattering measurements. Discrepancies with experimental values from electrochemical impedance spectroscopy measurements likely due to the porosity of the experimental samples.

R2Q2. The continuum simulations adopt a fixed GB width (2.5 nm) across all compositions. Given that ion transport across the GB depends largely on the assumed GB thickness, the authors should assess how the GB thickness influences the ion transport.

Your comments reveal a critical consideration in our multiscale modeling framework. A rigorous determination of grain boundary width for solid-state electrolytes remains beyond the current state of knowledge. Therefore, we have carefully selected the grain boundary width and additionally performed a sensitivity analysis to assess its impact.

i. Originated from grain boundary atomistic models after relaxation.

   Following our simulation workflow, grain boundary atomistic models are relaxed via molecular dynamics simulations for over 1 ns. During relaxation, local structures in the grain boundary region are optimized energetically, reflected by changes in averaged excess entropy and atomic energy of Li ions. The grain boundary width can then be estimated based on grain boundary profiles, as shown in Fig. 7 of our previous foundational study, Phys. Rev. Mater. 2024, **8**, 115407 [1].

   Different from our foundational study that only three grain boundaries were investigated, in the present study, we have extended the investigation to more complex grain boundaries indicated by large Σ values and even random grain boundaries embedded in polycrystal models. To enable consistent comparison among the grain boundaries sampled in the present study, a uniform grain boundary width of 2.5 nm was employed throughout.

ii. Consistency across atomistic and continuum scales.

   The grain boundary width of 2.5 nm *and* the corresponding bulk and grain boundary local diffusivities, derived from atomistic models, are passed to the continuum model for finite element simulations, thereby ensuring consistency across atomistic and continuum scales. This important information has been emphasized in Figure 1 in the revised manuscript.

iii. Consistency between experiments and simulations.

   We quantified the sensitivity of our predictions to different grain boundary widths using the multiscale modeling framework (Fig. 9). Reducing the grain boundary width from 2.5 nm to 1.5 nm yields similar Li-ion diffusion barriers, consistent with experimental measurements. In contrast, a further reduction to 1 nm leads to an overestimation of the diffusion barriers relative to experiments. For polycrystals with small grains, diffusivities obtained from all-atom molecular dynamics simulations agree well with those predicted by our multiscale framework using a grain boundary width of 2.5 nm. Smaller widths of 1 nm and 1.5 nm introduce inconsistencies between atomistic and continuum-scale simulations. These results justify our choice of grain boundary width and are included in the Supplementary Information for reference.

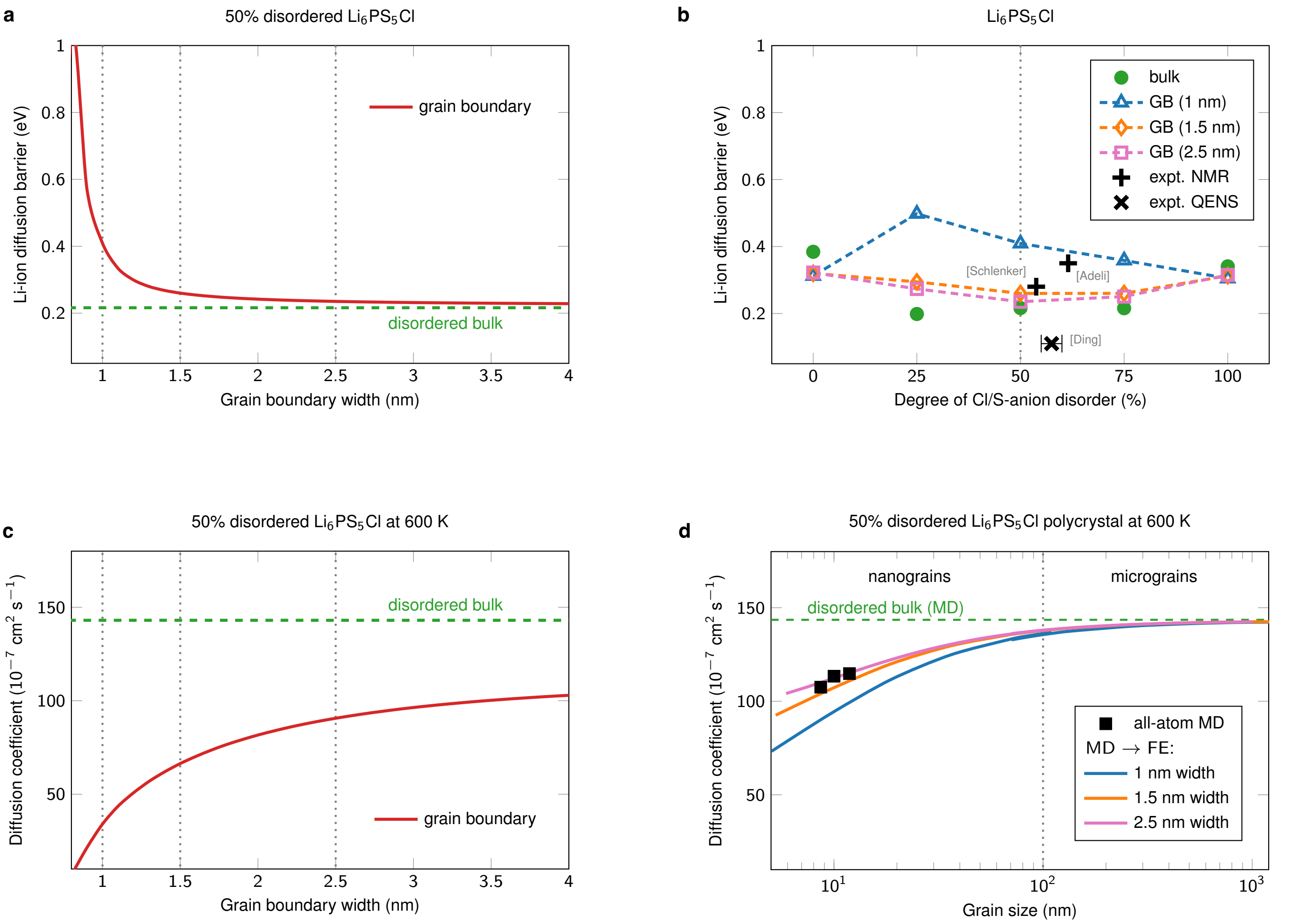

FIG. 9. **Sensitivity analysis of grain boundary width.** A grain boundary width of 2.5 nm was derived from atomistic grain boundary structures and was adopted for the main results in this study. Results obtained using smaller widths of 1.5 and 1 nm are shown in the present figure for comparison. **a**, Predicted Li-ion diffusion barriers in grain boundaries estimated using different grain boundary widths, exemplified for Li_6PS_5Cl with 50% anion disorder. **b**, Li-ion diffusion barriers in Li_6PS_5Cl as a function of anion disorder. Grain boundary widths of 1.5 nm and 2.5 nm yield similar results and are consistent with experimental values, whereas a width of 1 nm leads to overestimated barriers. **c**, Grain boundary diffusivities at 600 K estimated using different grain boundary widths. **d**, Macroscopic diffusivities of 50% anion-disordered Li_6PS_5Cl polycrystals at 600 K. The use of smaller grain boundary widths of 1.5 and 1 nm leads to inconsistencies between all-atom atomistic and continuum-scale predictions.

`Line 73`, Section **Main**:

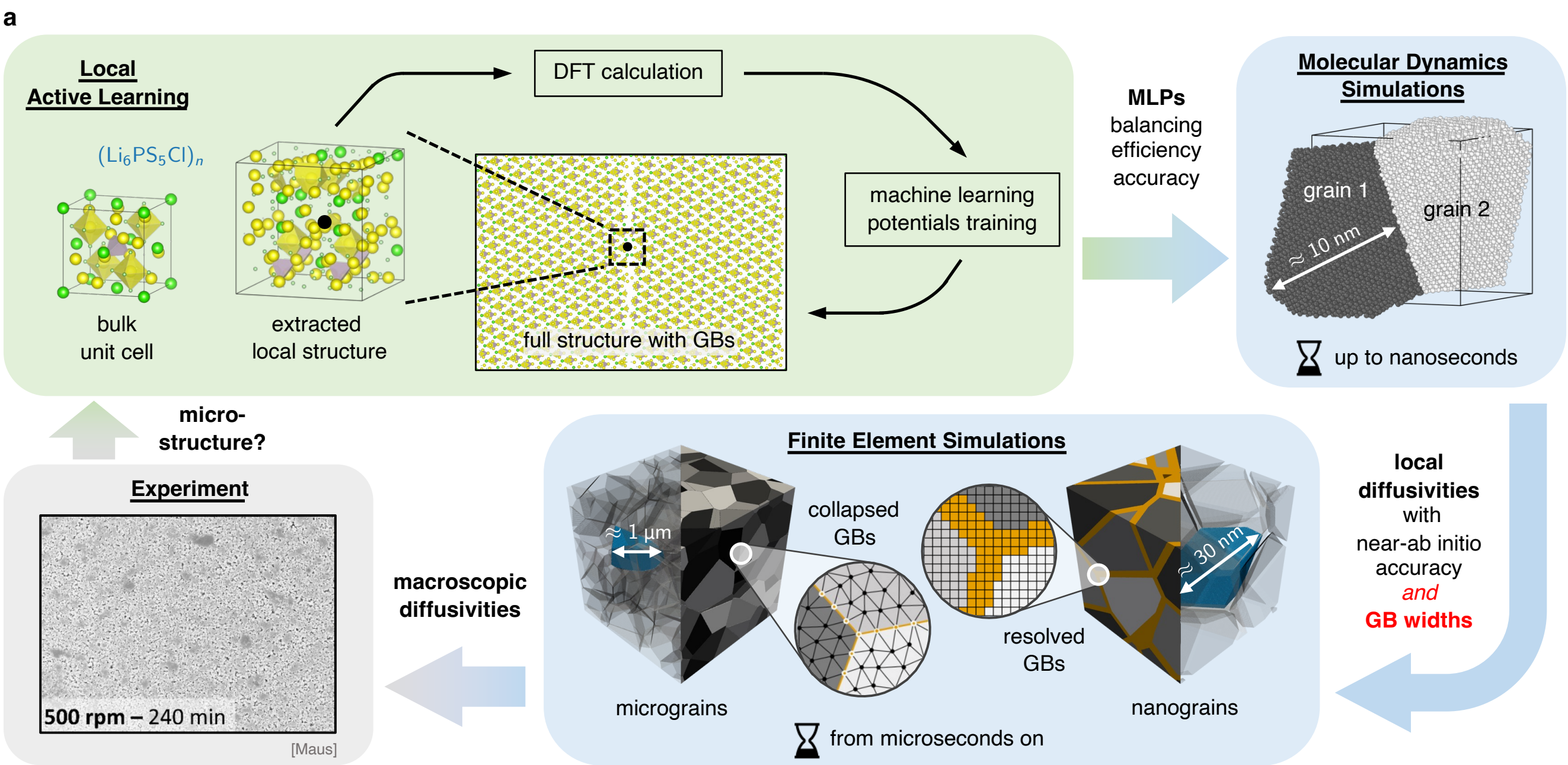

Fig. 1. **a**, Machine-learning potentials (MLPs) balancing accuracy and efficiency are developed with closed-loop active learning, based on density-functional theory (DFT) calculations of local structures extracted from full microstructures with grain boundaries (GBs). These potentials are applied in molecular dynamics (MD) simulations to obtain local Li-ion diffusivities. Finite element (FE) simulations, parameterized with MD-derived diffusivities and GB widths, are then performed on micrograins with collapsed GBs and on nanograins with resolved GBs using Fourier-accelerated nodal solvers, yielding macroscopic diffusivities.

`Line 197`, Section **Supplementary Note 4**:

Sensitivity to the GB width can be straightforwardly quantified within our multiscale modeling framework. At the atomistic scale, GB diffusivities derived from polycrystalline models depend on the assumed GB width. For a two-grain polycrystalline model with cubic geometry, the chosen GB widths and the corresponding calculated GB volume fractions are summarized in Table S2. Figure 9 shows the predictions obtained using different GB widths across atomistic and continuum scales. Reducing the GB width from 2.5 nm to 1.5 nm yields similar Li-ion diffusion barriers, consistent with experimental measurements. In contrast, a further reduction to 1 nm leads to an overestimation of the diffusion barriers relative to experiment. For polycrystals with small grains, diffusivities obtained from all-atom MD simulations agree well with those predicted by our multiscale framework using a GB width of 2.5 nm. Smaller widths of 1 nm and 1.5 nm introduce inconsistencies between atomistic and continuum-scale simulations.

R2Q3. The proposed linear correlation between Ea and anion radius is inferred from only three compositions in the anion-ordered phase, which appears not very convincing to establish a compelling trend. Additional corroboration is needed—for example, reporting activation energies for compositions spanning different degrees of anion disorder—to demonstrate the generality of this linear relationship.

We fully understand your concern that the linear correlation may not be robust to establish a general trend across the chemical space. To address this point, we have expanded our analysis by developing additional machine learning potentials and performing further diffusivity calculations for Li_6PS_5Br and Li_6PS_5I at 50% and 100% degrees of anion disorder. The corresponding activation energies are shown in Fig. 10.

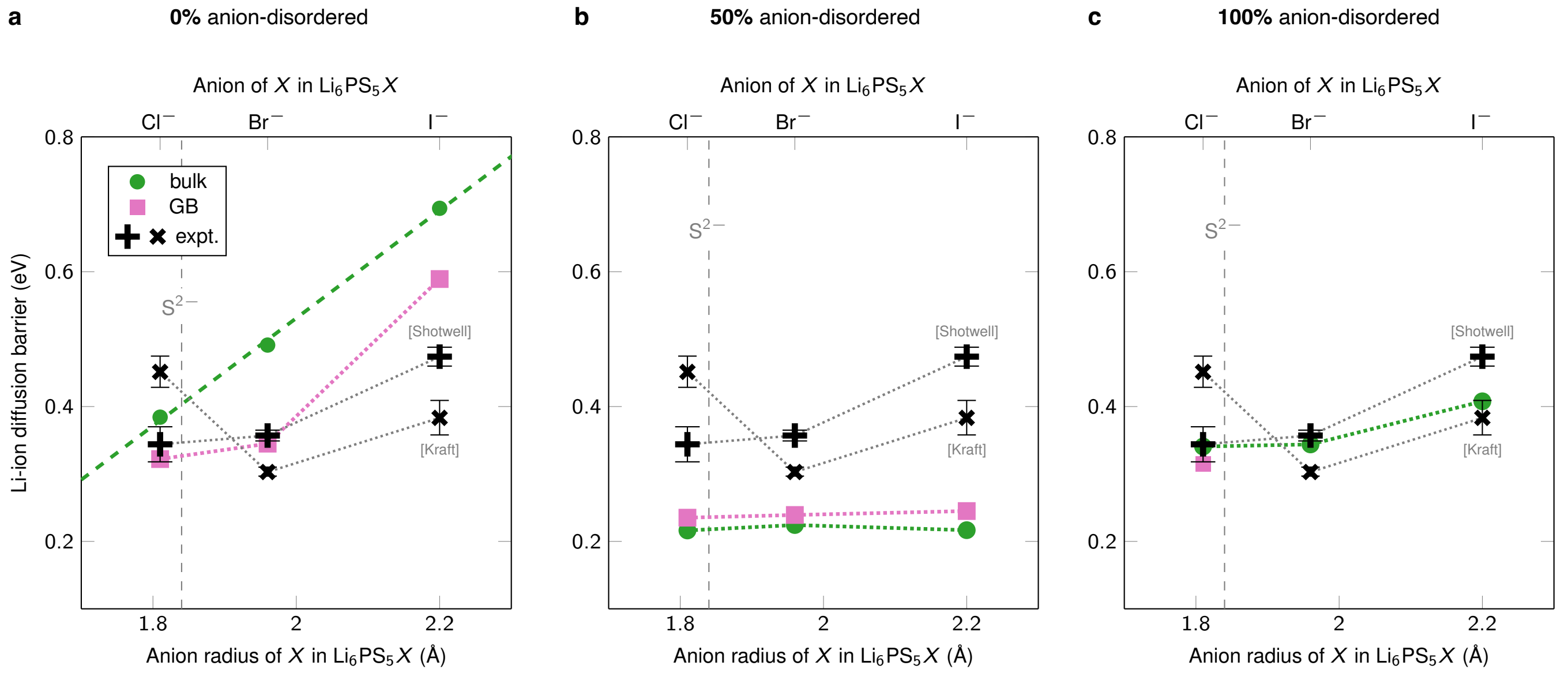

FIG. 10. **Influence of halide species on Li-ion diffusion barriers for argyrodites with different anion disorder.** Results are shown for Li_6PS_5X with $X \in$ Cl, Br, I, with (**a**) 0%, (**b**) 50%, and (**c**) 100% anion disorder. Consistency is observed across all argyrodites for the activation energy as a function of anion disorder. The bulk diffusion barriers decrease from 0% to 50% anion disorder and then increase from 50% to 100%. Grain boundaries hinder Li-ion diffusion at 50% anion disorder, while they enhance diffusion at 0%. The Li-ion diffusion barrier increases linearly with anion radius in anion-ordered bulk argyrodites, while exhibiting more complex behavior in other cases. Experimental values from electrochemical impedance spectroscopy measurements reported by Shotwell *et al.* [29] and Kraft *et al.* [30] are compared.

i. Consistent impacts of anion disorder and grain boundaries.

   For each types of Li_6PS_5X ($X \in$ Cl, Br, I), consistent results are shown regarding the impact of anion disorder and grain boundaries. The bulk diffusion barriers decrease from 0% to 50% anion disorder and them increase from 50% to 100%. Grain boundaries hinder Li-ion diffusion at 50% anion disorder, while they enahnce diffusion at 0%. These trends are consistent with a previous *ab initio* study focusing on Li_6PS_5Br [16].

ii. Anion chemistry becomes more complex with anion disorder.

   Additional results reveal that the dependence of the Li-ion diffusion barrier on anion radius becomes more intricate in argyrodites with anion disorder. At 50% disorder, where three-dimensional diffusion pathways are present [31], the diffusion barrier remains relatively low and shows no clear dependence on anion radius. Moreover, for the fully disordered (100%) case, a positive correlation emerges, albeit without a clear linear trend. These findings are consistent with recent experimental measurements reported by Shotwell *et al.* [29] and shown in Fig. 10. Overall, our results provide a more nuanced picture of anion chemistry by decoupling the effects of anion radius and disorder. These findings have been incorporated into the revised manuscript, and the corresponding sentences have been corrected accordingly.

`Line 13`, Section **Abstract**:

Accurate lightweight machine-learning potentials—developed via closed-loop active learning for exemplar argyrodites Li_6PS_5X, $X \in \{Cl, Br, I\}$—are employed to integrate molecular dynamics with finite element simulations. We find that diffusion barriers of the anion-ordered bulk scale linearly with anion radius. Grain boundaries exert opposite effects depending on the bulk: enhancing ion diffusion in low-diffusivity phases but suppressing it in fast-diffusing ones.

`Line 207`, Section **Results**:

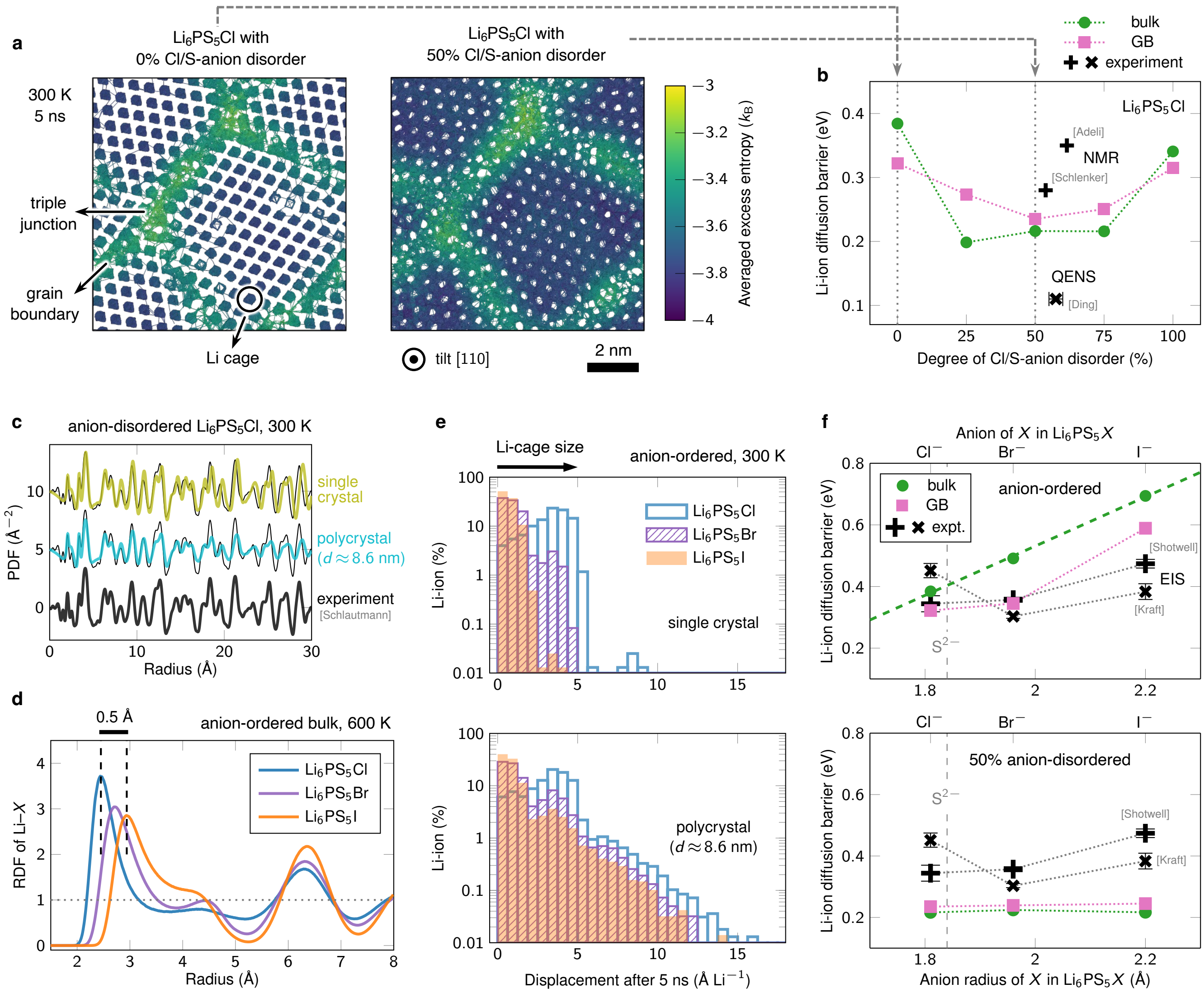

f, Decoupling anion ordering, anion species, and GB effects on Li-ion diffusion barriers. For anion-ordered argyrodites, the diffusion barrier increases linearly with anion radius in the bulk. GBs generally enhance diffusion, with a magnitude that depends on the anion species. For anion-disordered argyrodites, the diffusion barrier remains low and shows no clear dependence on anion radius. The predicted trends agree with the experimental values of Shotwell *et al.* [29], but differ from those of Kraft *et al.* [30]; both were obtained via electrochemical impedance spectroscopy (EIS).

`Line 258`, Section **Results**:

Including Li_6PS_5Br reveals a clear linear scaling of Li-ion diffusion barrier with anion radius (Fig. 3f), with an increase of 0.1 Å leading to an ∼80 meV higher barrier. A linear trend also holds for GB formation energy (Supplementary Fig. S18). By contrast, this trend is not apparent in anion-disordered argyrodites.

Our systematic study enables decoupling the effects of anion ordering, anion species, and GBs. In anion-ordered phases, GBs disrupt Li-ion cages in all Li_6PS_5X, leading to Li-ion displacements exceeding 5 Å. These effects are anion-dependent. Diffusion is most enhanced at Br GBs, which reduce the diffusion barrier by nearly 0.2 eV, compared with reductions of less than 0.1 eV in the Cl and I systems. For anion-ordered Li_6PS_5I, experimental values are more than 0.1 eV lower than the predicted GB value, likely due to additional defects that further enhance Li-ion transport [29, 32]. By contrast, in anion-disordered phases, GBs introduce a nearly uniform additional barrier of ∼0.02 eV.

`Line 381`, Section **Discussion and conclusion**:

Our results reveal a linear dependence between diffusion barrier and the anion radius in ordered argyrodites. A large radius mismatch between S^{2-} and X^- obstructs Li^+ pathways and increases the diffusion barrier, consistent with the observation for K_3OX [33]. This trend contrasts with LISICON-type solid electrolytes, where increasing the cation radius lowers the diffusion barrier to as low as 0.1 eV [34]. Our finding indicates that lattice polarizability, tunable through halide substitution, may not be the dominant factor governing Li-ion diffusivity in argyrodites, contrary to earlier suggestions [30]. ~~Achieving a more uniform distribution of ionic radii in the sublattice may thus advantageous for developing next-generation solid electrolytes, such as those incorporating high-entropy concepts.~~

R2Q4. Please add the corresponding analysis for Li6PS5Br in Fig. 3d and 3e (or, alternatively, in the Supporting Information).

Thank you for your suggestion. We have additionally performed radial distribution function and displacement distribution analyses for Li_6PS_5Br (Fig. 11). The results show that Li_6PS_5Br exhibits intermediate behavior between Li_6PS_5Cl and Li_6PS_5I. We have included these results in the main text, which provides a more complete picture for a broader readership.

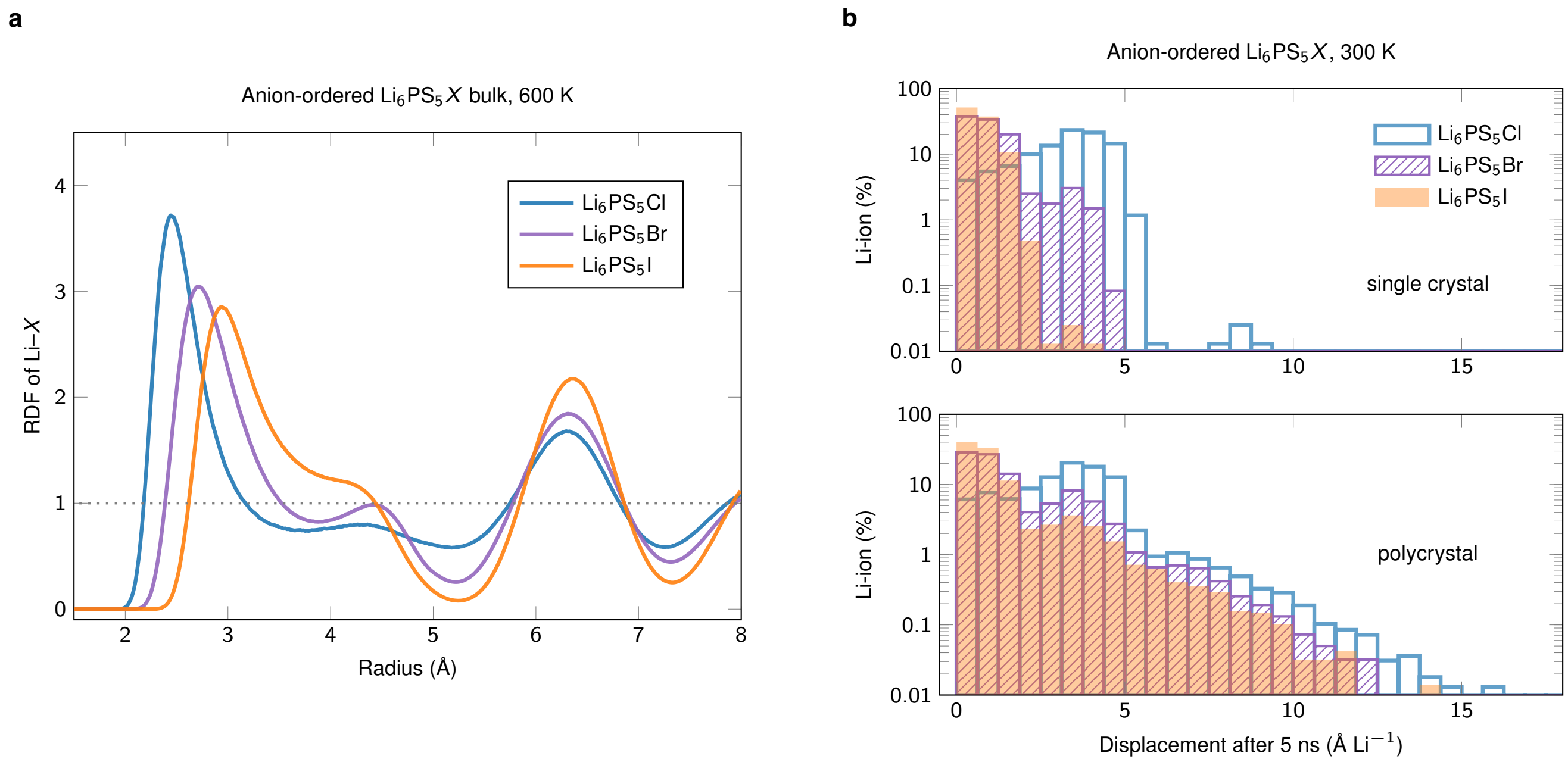

FIG. 11. **Comparison of Li-ion dynamics in anion-ordered argyrodites with different halogens. a**, Time-averaged radial distribution functions (RDFs) of Li^+–halide pairs. Molecular dynamics simulations at 600 K were used for structural sampling. **b**, Distribution of Li-ion displacements after molecular dynamics at 300 K over 5 ns. Results from bulk and polycrystal simulation models are compared. For both RDFs and displacement distributions, Li_6PS_5Br exhibits intermediate behavior between Li_6PS_5Cl and Li_6PS_5I.

`Line 207`, Section **Results**:

Fig. 3. **d**, Time-averaged Li^+–halide radial distribution functions (RDFs) of bulk Li_6PS_5X obtained from MD at 600 K. The first Li^+–halide coordination peak in Li_6PS_5I is attenuated and at a longer distance ($\sim$+0.5 Å) compared to that in Li_6PS_5Cl. Li_6PS_5Cl exhibits a attenuated Li–X RDF than Li_6PS_5I beyond 4 Å. The curve for Li_6PS_5Br lies in between those of the Cl and I argyrodites.**e**, Distribution of Li-ion displacements in anion-ordered Li_6PS_5X with $X \in \{Cl, Br, I\}$ after MD at 300 K over 5 ns. Li_6PS_5Cl and Li_6PS_5Br exhibit intra-cage diffusion, as indicated by the Li-cage size ($\sim$5 Å), whereas Li_6PS_5I shows primarily local hopping between adjacent Li-ion sites. GBs enhance Li-ion diffusion in all polycrystalline argyrodites, and the increase is more pronounced in Li_6PS_5Cl, where more Li ions show inter-cage diffusion.

R2Q5. The manuscript considers anion disorder only for Li6PS5Cl, while anions in Li6PS5Br and Li6PS5I are considered to be ordered. Table 1 shows that GB transport can differ significantly between ordered and disordered phases. In this context, comparing the experimentally measured activation energy of Li6PS5Br—typically associated with an anion-disordered phases—with a simulated GB activation energy derived from an anion-ordered model seems unreasonable.

We fully agree that, for comparisons with experiments, the anion-disorder effect should be considered for both Li_6PS_5Cl and Li_6PS_5Br. We have additionally performed calculations for bulk and polycrystalline Li_6PS_5Br with 50% anion disorder using our multiscale modeling framework.

Table I shows an overview of the predicted diffusion properties for argyrodites and comparisons to experiments. For bulk Li_6PS_5Br, the introduction of anion disorder drastically increase the room-temperature diffusivities from 3.9×10^{-11} to $1.9\,cm^2\,s^{-1}$, consistent with previous *ab initio* studies [16, 31] and experimental measurements [35]. Similar to Li_6PS_5Cl, grain boundaries in Li_6PS_5Br increase diffusivities for the anion-ordered phase while decrease

diffusivities for the anion-disordered phase, consistent with a previous *ab initio* study [16]. The predicted diffusivities of 50% anion-disordered Li_6PS_5Cl and Li_6PS_5Br are similar (2.2×10^{-7} vs. 1.5×10^{-7} cm^2s^{-1}), converted to jumping frequencies (1.8×10^9 vs. 1.3×10^9 Hz) consistent with experimental results, where similar jumping frequencies were observed (2.5×10^7 vs. 1.4×10^7 Hz).

TABLE I. **Predicted Li-ion self-diffusivities and derived quantities of argyrodite solid electrolytes at 300 K.** The "anion disorder" row indicates the degree of X/S-anion disorder. Diffusion coefficients D^{GB} are from MD simulations of polycrystals, representing averages over all GB structures within them. The GB-to-bulk ratio quantifies the impact of GBs on diffusion in the polycrystal with ↑ and ↓ for increase and decrease, respectively. The f rows show the predicted characteristic Li-ion hopping frequencies, which are consistent with experimental values from Shotwell *et al.* [29].

System	Li_6PS_5Cl				
Anion disorder (%)	0	25	50	75	100
D^{bulk} (10^{-7} cm^2s^{-1})	0.010	2.153	2.184	1.808	0.048
D^{GB} (10^{-7} cm^2s^{-1})	0.092	0.401	0.959	0.690	0.136
D^{GB}/D^{bulk}	8.79 ↑	0.19 ↓	0.44 ↓	0.38 ↓	2.85 ↑
f^{bulk} (Hz)	8.61×10^6	1.77×10^9	1.80×10^9	1.49×10^9	3.92×10^7
f^{GB} (Hz)	7.57×10^7	3.30×10^8	7.90×10^8	5.68×10^8	1.12×10^8
$f^{experiment}$ (Hz) [Shotwell]	2.50×10^7				

System	Li_6PS_5Br		
Anion disorder (%)	0	50	100
D^{bulk} (10^{-7} cm^2s^{-1})	3.9×10^{-4}	1.522	0.053
D^{GB} (10^{-7} cm^2s^{-1})	0.025	0.436	-
D^{GB}/D^{bulk}	64.06 ↑	0.29 ↓	-
f^{bulk} (Hz)	3.19×10^5	1.25×10^9	4.38×10^7
f^{GB} (Hz)	2.04×10^7	3.57×10^8	-
$f^{experiment}$ (Hz) [Shotwell]	1.37×10^7		

System	Li_6PS_5I		
Anion disorder (%)	0	50	100
D^{bulk} (10^{-7} cm^2s^{-1})	6.2×10^{-7}	1.817	4.1×10^{-3}
D^{GB} (10^{-7} cm^2s^{-1})	3.7×10^{-5}	0.724	-
D^{GB}/D^{bulk}	59.25 ↑	0.40 ↓	-
f^{bulk} (Hz)	5.05×10^2	1.48×10^9	3.31×10^6
f^{GB} (Hz)	2.99×10^4	5.88×10^8	-
$f^{experiment}$ (Hz) [Shotwell]	1.27×10^5		

We have also compared the newly calculated bulk and grain boundary diffusion barriers for anion-disordered Li_6PS_5Br to experimental values, as shown in Fig. 10. Additionally, the new data for anion-disordered Li_6PS_5Br are passed to finite element simulations. Diffusivities of polycrystalline Li_6PS_5Br with anion disordered were obtained, as shown in Fig. 4. These new results and comparisons for Li_6PS_5Br have been added in the revised manuscript.

`Line 208`, Section **Results**:

Table 1. **Predicted Li-ion self-diffusivities and derived quantities of argyrodite solid electrolytes at 300 K.**

System	Li_6PS_5Cl					Li_6PS_5Br	Li_6PS_5I
Anion disorder (%)	0	25	50	75	100	50	0
D^{bulk} (10^{-7} cm^2s^{-1})	0.010	2.153	2.184	1.808	0.048	1.522	6.2×10^{-7}
D^{GB} (10^{-7} cm^2s^{-1})	0.092	0.401	0.959	0.690	0.136	0.436	3.7×10^{-5}
D^{GB}/D^{bulk}	8.79 ↑	0.19 ↓	0.44 ↓	0.38 ↓	2.85 ↑	0.29 ↓	59.25 ↑
f^{bulk} (Hz)	8.61×10^6	1.77×10^9	1.80×10^9	1.49×10^9	3.92×10^7	1.25×10^9	5.05×10^2
f^{GB} (Hz)	7.57×10^7	3.30×10^8	7.90×10^8	5.68×10^8	1.12×10^8	3.57×10^8	2.99×10^4
$f^{experiment}$ (Hz) [Shotwell]			2.50×10^7			1.37×10^7	1.27×10^5

R2Q6. Please expand the discussion on the implications for the rational design and processing of argyrodite electrolytes. This work captures grain-size effects arising from bulk and GB diffusion, but does not include other microstructural factors that often vary with grain size and can strongly affect practical conductivities, such as porosity and interparticle contact.

We thank the reviewer for highlighting the importance of additional microstructural factors that are not explicitly included in the present study. To address this point, we have incorporated the effect of porosity into the evaluation of macroscopic ionic conductivity on top of our simulation results, as detailed in our response to Reviewer#1 Q2.2.

Briefly, accounting for porosity and potentially other microstructural factors helps reduce the discrepancy between simulation and experimental measurements Our results indicate that grain boundaries exert only a minor hindering effect on Li-ion diffusion in argyrodites with anion disorder, in line with experimental observations [9, 36]. Relevant discussions have been added to the revised manuscript, where we also outline how our multiscale framework can be straightforwardly extended to incorporate additional microstructural factors.

[1] Y. Ou, Y. Ikeda, L. Scholz, S. Divinski, F. Fritzen, and B. Grabowski, Atomistic modeling of bulk and grain boundary diffusion in solid electrolyte Li_6PS_5Cl using machine-learning interatomic potentials, Phys. Rev. Mater. **8**, 115407 (2024).
[2] J. Cheng, J. Luo, and K. Yang, Aimsgb: An algorithm and open-source python library to generate periodic grain boundary structures, Comput. Mater. Sci. **155**, 92 (2018).
[3] D. G. Brandon, The structure of high-angle grain boundaries, Acta Metall. **14**, 1479 (1966).
[4] A. Neumann, T. R. Hamann, T. Danner, S. Hein, K. Becker-Steinberger, E. Wachsman, and A. Latz, Effect of the 3D structure and grain boundaries on lithium transport in garnet solid electrolytes, ACS Appl. Energy Mater. **4**, 4786 (2021).
[5] T. Hamann, L. Zhang, Y. Gong, G. Godbey, J. Gritton, D. McOwen, G. Hitz, and E. Wachsman, The effects of constriction factor and geometric tortuosity on Li-ion transport in porous solid-state Li-ion electrolytes, Adv. Funct. Mater. **30**, 1910362 (2020).
[6] M. B. Dixit, M. Regala, F. Shen, X. Xiao, and K. B. Hatzell, Tortuosity effects in garnet-type $Li_7La_3Zr_2O_{12}$ solid electrolytes, ACS Appl. Mater. Interfaces. **11**, 2022 (2019).
[7] D. Hlushkou, A. E. Reising, N. Kaiser, S. Spannenberger, S. Schlabach, Y. Kato, B. Roling, and U. Tallarek, The influence of void space on ion transport in a composite cathode for all-solid-state batteries, J. Power Sources **396**, 363 (2018).
[8] T. W. Heo, A. Grieder, B. Wang, M. Wood, T. Hsu, S. A. Akhade, L. F. Wan, L.-Q. Chen, N. Adelstein, and B. C. Wood, Microstructural impacts on ionic conductivity of oxide solid electrolytes from a combined atomistic-mesoscale approach, npj Comput. Mater. **7**, 1 (2021).
[9] S. Ohno, T. Bernges, J. Buchheim, M. Duchardt, A.-K. Hatz, M. A. Kraft, H. Kwak, A. L. Santhosha, Z. Liu, N. Minafra, F. Tsuji, A. Sakuda, R. Schlem, S. Xiong, Z. Zhang, P. Adelhelm, H. Chen, A. Hayashi, Y. S. Jung, B. V. Lotsch, B. Roling, N. M. Vargas-Barbosa, and W. G. Zeier, How certain are the reported ionic conductivities of thiophosphate-based solid electrolytes? An interlaboratory study, ACS Energy Lett. **5**, 910 (2020).
[10] O. Cojocaru-Mirédin, J. Schmieg, M. Müller, A. Weber, E. Ivers-Tiffée, and D. Gerthsen, Quantifying lithium enrichment at grain boundaries in $Li_7La_3Zr_2O_{12}$ solid electrolyte by correlative microscopy, J. Power Sources **539**, 231417 (2022).
[11] Z. Huang, L. Chen, B. Huang, B. Xu, G. Shao, H. Wang, Y. Li, and C.-A. Wang, Enhanced performance of $Li_{6.4}La_3Zr_{1.4}Ta_{0.6}O_{12}$ solid electrolyte by the regulation of grain and grain boundary phases, ACS Appl. Mater. Interfaces **12**, 56118 (2020).
[12] C. Gao, X. Zhou, R. Yu, C. Li, X. Gao, W. Yang, D. Chao, and Y. Chen, Synergistic modulation of grain boundary and domain boundary enhances the ionic conductivity of $Li_{0.33}La_{0.56}TiO_3$ solid electrolyte, ACS Nano **19**, 10902 (2025).
[13] Y. Li, M. Lehmann, L. Cheng, T. A. Zawodzinski, J. Nanda, and G. Yang, Integrated electro- and chemical characterization of sulfide-based solid-state electrolytes, Mater. Adv. **5**, 9138 (2024).
[14] J. H. Kim, B. Jun, Y. J. Jang, S. H. Choi, S. H. Choi, S. M. Cho, Y.-G. Kim, B.-H. Kim, and S. U. Lee, Highly reliable and large-scale simulations of promising argyrodite solid-state electrolytes using a machine-learned moment tensor potential, Nano Energy **124**, 109436 (2024).
[15] R. Jalem, M. L. H. Chandrappa, J. Qi, Y. Tateyama, and S. P. Ong, Lithium dynamics at grain boundaries of β-Li_3PS_4 solid electrolyte, Energy Adv. **2**, 2029 (2023).
[16] M. Sadowski and K. Albe, Grain boundary transport in the argyrodite-type Li_6PS_5Br solid electrolyte: Influence of misorientation and anion disorder on Li ion mobility, Adv. Mater. Interfaces **11**, 2400423 (2024).
[17] M. Brinek, C. Hiebl, and H. M. R. Wilkening, Understanding the origin of enhanced Li-ion transport in nanocrystalline argyrodite-type Li_6PS_5I, Chem. Mater. **32**, 4754 (2020).
[18] J. A. Dawson, P. Canepa, M. J. Clarke, T. Famprikis, D. Ghosh, and M. S. Islam, Toward understanding the different influences of grain boundaries on ion transport in sulfide and oxide solid electrolytes, Chem. Mater. **31**, 5296 (2019).
[19] B. Gao, R. Jalem, H.-K. Tian, and Y. Tateyama, Revealing atomic-scale ionic stability and transport around grain boundaries of garnet $Li_7La_3Zr_2O_{12}$ solid electrolyte, Adv. Energy Mater. **12**, 2102151 (2022).
[20] K. Nakano, N. Tanibata, H. Takeda, R. Kobayashi, M. Nakayama, and N. Watanabe, Molecular dynamics simulation of Li-ion conduction at grain boundaries in NASICON-type $LiZr_2(PO_4)_3$ solid electrolytes, J. Phys. Chem. C **125**, 23604 (2021).
[21] J. A. Quirk and J. A. Dawson, Design principles for grain boundaries in solid-state lithium-ion conductors, Adv. Energy Mater. **13**, 2301114 (2023).
[22] I. Hanghofer, M. Brinek, S. L. Eisbacher, B. Bitschnau, M. Volck, V. Hennige, I. Hanzu, D. Rettenwander, and H. M. R. Wilkening, Substitutional disorder: Structure and ion dynamics of the argyrodites Li_6PS_5Cl, Li_6PS_5Br and Li_6PS_5I, Phys. Chem. Chem. Phys. **21**, 8489 (2019).
[23] P. Adeli, J. D. Bazak, A. Huq, G. R. Goward, and L. F. Nazar, Influence of aliovalent cation substitution and mechanical compression on Li-ion conductivity and diffusivity in argyrodite solid electrolytes, Chem. Mater. **33**, 146 (2021).
[24] B. D. Lee, J.-W. Lee, J. Park, M. Y. Cho, W. B. Park, and K.-S. Sohn, Argyrodite configuration determination for DFT and AIMD calculations using an integrated optimization strategy, RSC Adv. **12**, 31156 (2022).
[25] M. Jiang, Z.-W. Chen, A. Rao, L.-X. Chen, X.-T. Zu, and C. V. Singh, Se-doped Li_6PS_5Cl and $Li_{5.5}PS_{4.5}Cl_{1.5}$ with improved ionic conductivity and interfacial compatibility: a high-throughput DFT study, J. Mater. Chem. C **10**, 18294 (2022).
[26] T. Jeon, G. H. Cha, and S. C. Jung, Understanding the anion disorder governing lithium distribution and diffusion in an argyrodite Li_6PS_5Cl solid electrolyte, J. Mater. Chem. A **12**, 993 (2024).
[27] O. Korjus, S. Mitra, Q. Berrod, V. Vanpeene, M. Appel, L. Broche, S. Lyonnard, and C. Villevieille, Investigating the densification of Li_6PS_5Cl solid electrolyte through multi-scale characterization techniques, Energy Storage Mater. **82**, 104589 (2025).
[28] J. Ding, M. K. Gupta, C. Rosenbach, H.-M. Lin, N. C. Osti, D. L. Abernathy, W. G. Zeier, and O. Delaire, Liquid-like dynamics in a solid-state lithium electrolyte, Nat. Phys. **21**, 118 (2025).
[29] A. M. Shotwell, M. C. Schulze, P. Yox, C. Alaniz, and A. E. Maughan, Tetrahedral tilting and lithium-ion transport in

halide argyrodites prepared by rapid, microwave-assisted synthesis, Adv. Funct. Mater. **35**, 2500237 (2025).

[30] M. A. Kraft, S. P. Culver, M. Calderon, F. Böcher, T. Krauskopf, A. Senyshyn, C. Dietrich, A. Zevalkink, J. Janek, and W. G. Zeier, Influence of lattice polarizability on the ionic conductivity in the lithium superionic argyrodites Li_6PS_5X (X = Cl, Br, I), J. Am. Chem. Soc. **139**, 10909 (2017).

[31] B. J. Morgan, Mechanistic origin of superionic lithium diffusion in anion-disordered Li_6PS_5X argyrodites, Chem. Mater. **33**, 2004 (2021).

[32] V. Faka, M. T. Agne, M. A. Lange, D. Daisenberger, B. Wankmiller, S. Schwarzmüller, H. Huppertz, O. Maus, B. Helm, T. Böger, J. Hartel, J. M. Gerdes, J. J. Molaison, G. Kieslich, M. R. Hansen, and W. G. Zeier, Pressure-induced dislocations and their influence on ionic transport in Li^+-conducting argyrodites, J. Am. Chem. Soc. **146**, 1710 (2024).

[33] J. Zheng, J. Elgin, D. J. White, and Y. Wu, Ionic conductivities of potassium antiperovskites K_3OX (X = Cl, Br, and I): Bottleneck tuning vs anion polarizability, ACS Appl. Energy Mater. **8**, 12217 (2025).

[34] J. C. Bachman, S. Muy, A. Grimaud, H.-H. Chang, N. Pour, S. F. Lux, O. Paschos, F. Maglia, S. Lupart, P. Lamp, L. Giordano, and Y. Shao-Horn, Inorganic solid-state electrolytes for lithium batteries: Mechanisms and properties governing ion conduction, Chem. Rev. **116**, 140 (2016).

[35] A. Gautam, M. Sadowski, N. Prinz, H. Eickhoff, N. Minafra, M. Ghidiu, S. P. Culver, K. Albe, T. F. Fässler, M. Zobel, and W. G. Zeier, Rapid crystallization and kinetic freezing of site-disorder in the lithium superionic argyrodite Li_6PS_5Br, Chem. Mater. **31**, 10178 (2019).

[36] Y. Wang, H. Hao, K. G. Naik, B. S. Vishnugopi, C. D. Fincher, Q. Yan, V. Raj, H. Celio, G. Yang, H. Fang, Y.-M. Chiang, F. A. Perras, P. Jena, J. Watt, P. P. Mukherjee, and D. Mitlin, Mechanical milling-induced microstructure changes in argyrodite LPSCl solid-state electrolyte critically affect electrochemical stability, Adv. Energy Mater. **14**, 2304530 (2024).
